# Adhesives and Sealants in Packaging: Advanced Materials, Performance, and Emerging Technologies (Part II)

**DOI:** 10.3390/ma19153320

**Published:** 2026-08-04

**Authors:** Calogero Volpe, Leonardo Pagnotta

**Affiliations:** Department of Mechanical, Energy and Management Engineering, University of Calabria, Arcavacata, 87036 Rende, Italy; calogero.volpe@unical.it

**Keywords:** adhesives, sealants, multilayer packaging, hot-melt adhesives, bio-based adhesives, compostable sealants, debond-on-demand adhesives, circular packaging, food packaging, recyclability

## Abstract

This second part extends the system-level framework established in Part I by examining advanced adhesive and sealant technologies through a performance-, circularity-, and application-oriented perspective relevant to contemporary packaging systems. While Part I focused on material classification, bonding and sealing mechanisms, regulatory aspects, and interfacial design principles, the present review analyses how advanced adhesive and sealant systems behave under realistic converting, sealing, service, recycling, and end-of-life conditions. Particular attention is devoted to bio-based and compostable adhesives, recyclable mono-material architectures, advanced multilayer sealants, debond-on-demand systems, and smart or reversible interfaces designed to support circular packaging strategies. The review critically discusses the principal adhesive and sealant performance metrics—including bond strength, seal strength, seal initiation temperature (SIT), hot-tack behaviour, cohesive durability, processing robustness, and hydrothermal resistance—in relation to packaging reliability, barrier preservation, processability, and compatibility with industrial converting operations. The analysis additionally addresses interfacial failure mechanisms, recyclability constraints associated with multilayer structures, food-contact compliance, migration and non-intentionally added substances (NIAS), and the growing role of design-for-disassembly and circularity-oriented interfacial engineering. Emerging transition strategies involving waterborne systems, low-migration formulations, recyclable sealants, dynamic covalent networks, and controlled debonding technologies are evaluated in terms of their potential to reconcile packaging performance with sustainable material management. By integrating material-specific developments with system-level packaging considerations, this review highlights how adhesive and sealant interfaces increasingly represent critical design variables governing the balance between mechanical performance, sealing reliability, processability, recyclability, compostability, and circularity in next-generation packaging systems.

## 1. Introduction

**Figure 1:**
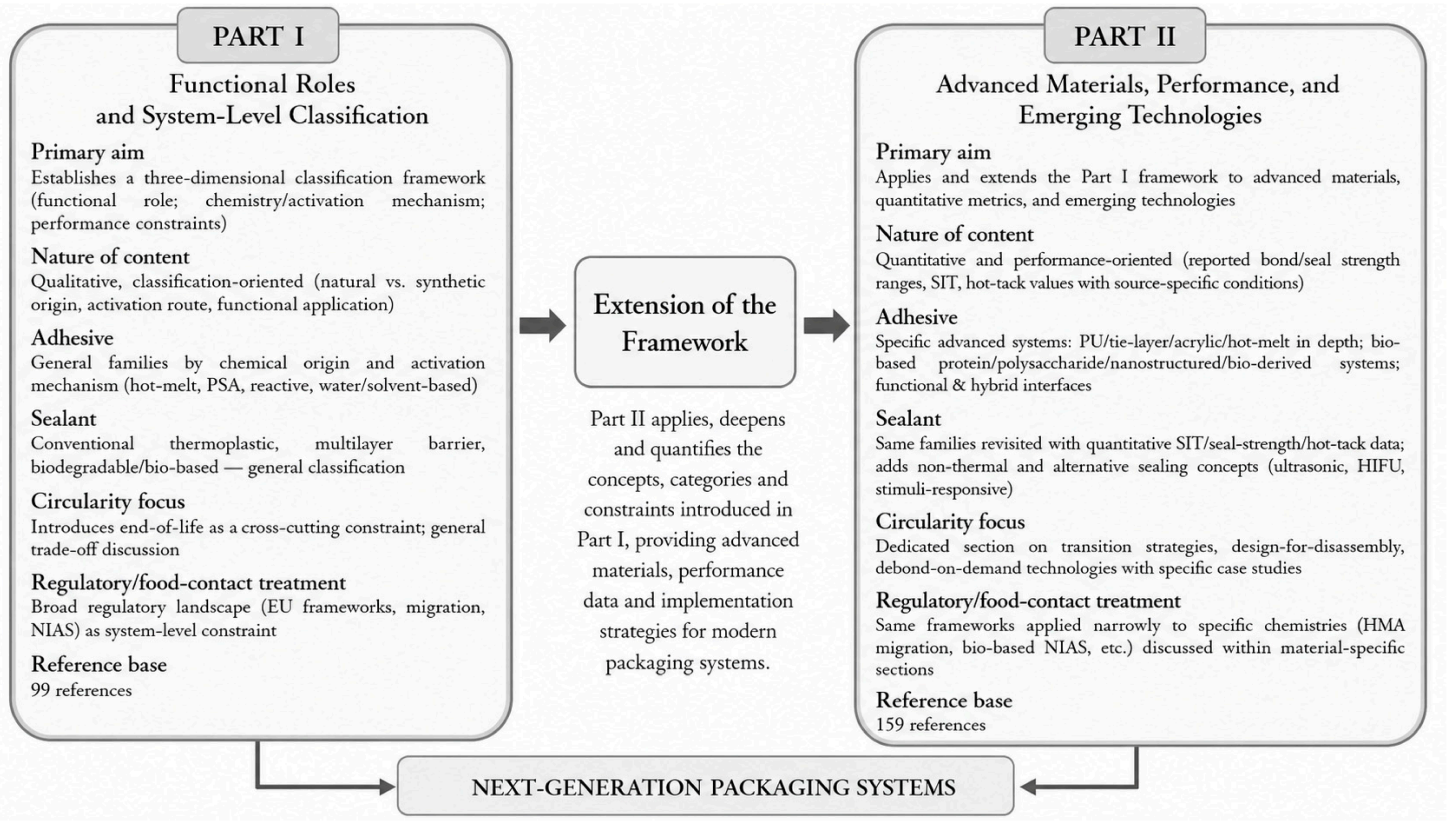
From Part I to Part II: extension of a system-level framework to advanced materials, performance and emerging technologies.

Adhesives and sealants have become critical interfacial technologies in modern packaging systems, directly influencing multilayer integrity, barrier continuity, processability, packaging reliability, recyclability, and end-of-life compatibility. Building upon the system-level framework introduced in Part I [1], the present review focuses on advanced adhesive and sealant materials, quantitative performance metrics, and emerging interfacial technologies relevant to next-generation packaging applications. Figure 1 summarises the main differences in scope, content, and depth between Part I and Part II.

The progressive transition from monolithic structures toward multilayer and hybrid packaging architectures has increased dependence on engineered interfacial systems capable of accommodating substrate incompatibilities, thermal stresses, mechanical loading, and severe processing conditions such as retort and pasteurisation [2,3]. Simultaneously, growing regulatory and environmental pressures associated with food-contact safety, chemical migration, volatile emissions, recyclability, and circular packaging strategies have transformed adhesives and sealants into both enabling technologies and potential limitations for sustainable packaging development [4,5,6].

Consequently, recent research has increasingly focused on advanced interfacial systems capable of balancing performance and sustainability requirements. These include bio-based and compostable adhesives, compostable hot-melt systems, recyclable tie-layer technologies, advanced multilayer sealants, and reversible or triggerable interfaces designed to facilitate controlled delamination and material recovery. Parallel advances in quantitative characterisation and modelling approaches have enabled systematic evaluation of bond strength, seal strength, seal initiation temperature, hot-tack behaviour, curing kinetics, sealing windows, and interfacial failure mechanisms under industrially relevant conditions [7]. Increasing attention has also been directed toward recyclability–performance trade-offs and the development of interfacial systems compatible with mono-material and circular packaging strategies.

Despite the growing body of literature, comparative analyses integrating material composition, processing behaviour, quantitative performance metrics, and end-of-life implications remain fragmented across chemistries and application sectors, particularly for emerging bio-based systems, debondable adhesives, recyclable multilayer interfaces, and advanced sealant technologies for recyclable or compostable packaging architectures [8].

Before proceeding, it is important to clarify that the sustainability-related terms used throughout this review are not equivalent or automatically interdependent. Bio-based origin describes only the renewable sourcing of a material’s feedstock and does not by itself imply biodegradability. Biodegradability refers to the intrinsic capacity of a material to degrade under specified environmental conditions, whereas compostability is a certified performance criterion—evaluated against standards such as EN 13432 [9] or ASTM D6400 [10]—requiring defined disintegration, biodegradation, and ecotoxicity thresholds to be met under industrial or home-composting conditions. Recyclability, in turn, concerns compatibility with existing mechanical or chemical recycling streams and is largely independent of bio-based content or biodegradability. Mono-material design is a structural strategy that facilitates recyclability but does not guarantee it, since compatibilizers, coatings, tie layers, or additives may still hinder effective separation and reprocessing. Unless explicitly stated as certified or experimentally validated at the level of the complete packaging article, statements regarding compostability, recyclability, or biodegradability in this review should therefore be understood as describing theoretical or component-level compatibility rather than demonstrated end-of-life performance of the finished packaging system (see also Section 3.3.4 and Section 6).

**Figure 2:**
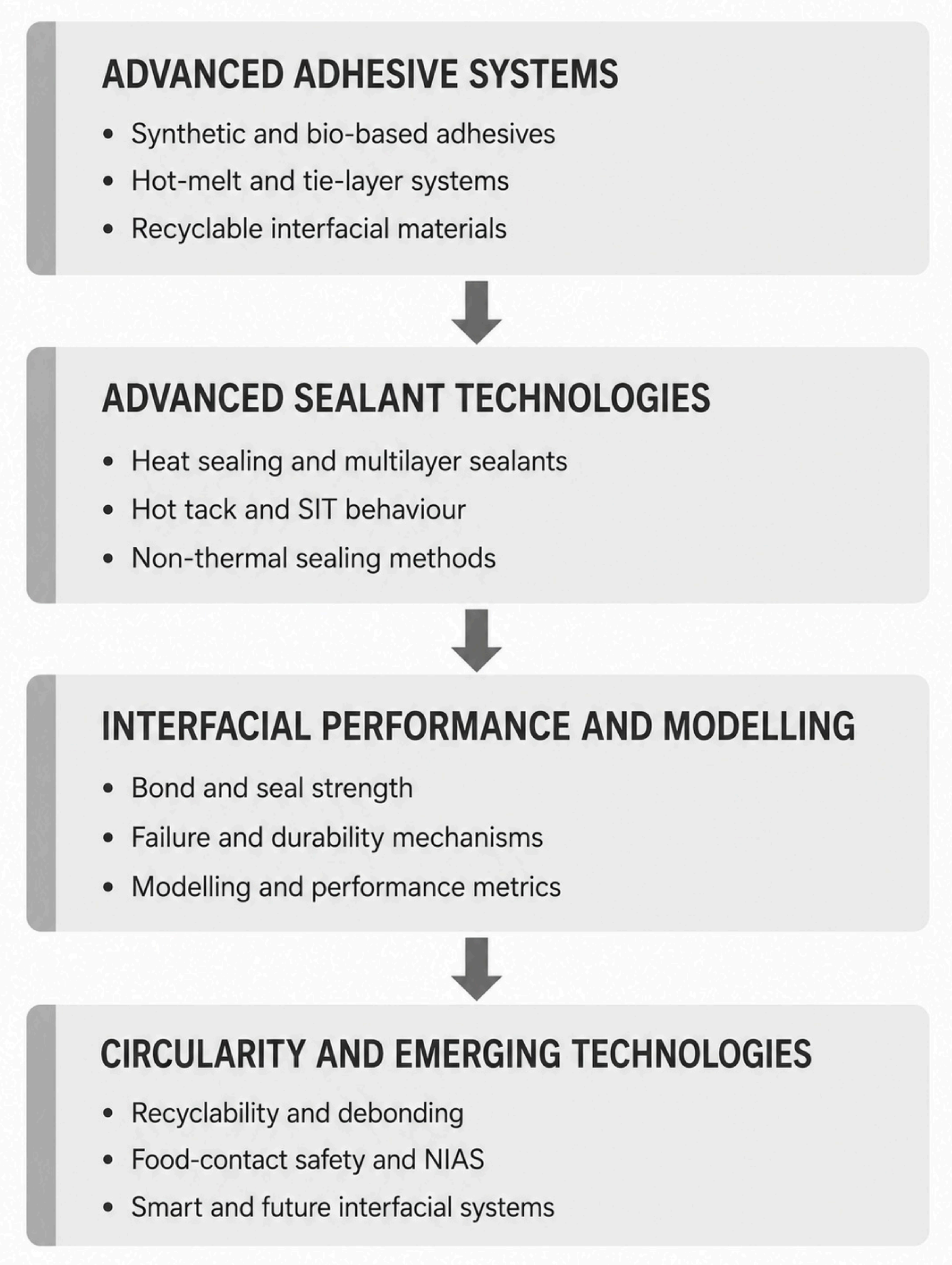
Logical roadmap of Part II of the review.

Figure 2 summarises the logical structure of Part II, highlighting the interplay between interfacial engineering, converting compatibility, packaging reliability, and circularity that underpins the following sections.

### Methodological Note

The reference base for this second part was assembled through a targeted literature search focused on recent advances in adhesive and sealant technologies for packaging applications. In continuity with Part I, the survey drew on Scopus, Web of Science, ScienceDirect, Google Scholar, publisher platforms, and selected technical or regulatory sources. However, the search strategy was specifically oriented toward advanced material systems, quantitative performance data, interfacial behaviour, processability, circularity, and emerging technologies.

Representative search terms combined adhesive- and sealant-related keywords with packaging-specific descriptors, including bio-based systems, compostable hot-melts, recyclable multilayers, smart or reversible interfaces, seal initiation temperature, hot-tack behaviour, bond and seal strength, NIAS, migration, recyclability, and compostability. Additional sources were identified through citation tracking from key references already considered in Part I and from recent reviews dealing with advanced packaging materials and interfacial technologies.

Representative examples of the Boolean search strings used include: (“bio-based adhesive*” OR “compostable adhesive*” OR “compostable hot-melt*”) AND (“packaging” OR “food packaging”); (“seal initiation temperature” OR “hot-tack” OR “heat sealing”) AND (“biodegradable” OR “PLA” OR “PBAT” OR “PBS”) AND “packaging”; and (“debond-on-demand” OR “debondable adhesive*” OR “reversible adhesive*” OR “dynamic covalent”) AND (“recycling” OR “packaging”).

Records were screened by title and abstract for relevance to packaging adhesive and sealant technologies, and studies were included when they addressed material composition, processing behaviour, quantitative performance metrics, interfacial or sealing mechanisms, food-contact and regulatory aspects, or end-of-life and circularity considerations relevant to packaging. Studies were excluded when they addressed adhesive or sealant systems exclusively developed for non-packaging sectors (e.g., structural, automotive, or biomedical applications) without transferable concepts or methods relevant to packaging, or when they were not accessible in full text. Given the breadth of the topic and the intent to provide an integrative, application-oriented synthesis rather than an exhaustive quantitative synthesis, this review follows a structured narrative approach rather than a formal systematic-review protocol; consequently, a PRISMA-type flow diagram reporting the exact number of initially retrieved records was not maintained, and source selection instead relied on the iterative screening and citation-tracking procedure described above.

The literature survey mainly covered publications from 1995 to 2026, with earlier sources retained only where necessary for continuity with foundational concepts discussed in Part I.

Overall, 159 scientific and technical references were considered in this second part. The three European regulatory frameworks introduced in Part I—the Packaging and Packaging Waste Regulation (PPWR) [11], Regulation (EC) No. 1935/2004 [12], and Regulation (EC) No. 2023/2006 [13]—are recalled only where relevant to contextualise material compliance, food-contact safety, and end-of-life constraints.

**Figure 3:**
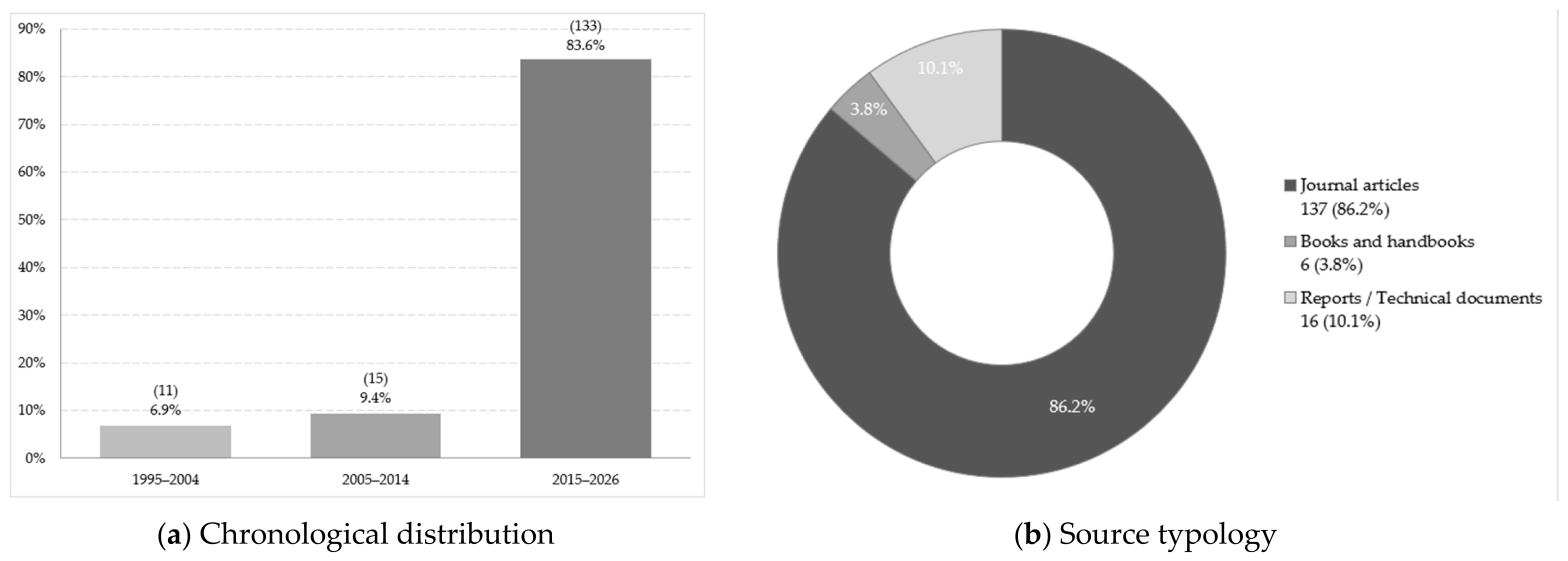
Documentary basis of the review. (**a**) Chronological distribution of the 159 scientific and technical references considered in Part II, showing the predominance of recent publications. (**b**) Source typology, highlighting the prevalence of journal articles, with books, handbooks, conference papers, and technical or regulatory documents used selectively to support methodological, industrial, and regulatory discussion.

The documentary profile is summarised in Figure 3, which reports the chronological distribution and source typology of the references considered. The prevalence of recent journal articles reflects the specific focus of Part II on advanced materials, quantitative performance assessment, and emerging technologies, while books, handbooks, and technical documents were used selectively to support methodological, industrial, or regulatory discussion.

**Figure 4:**
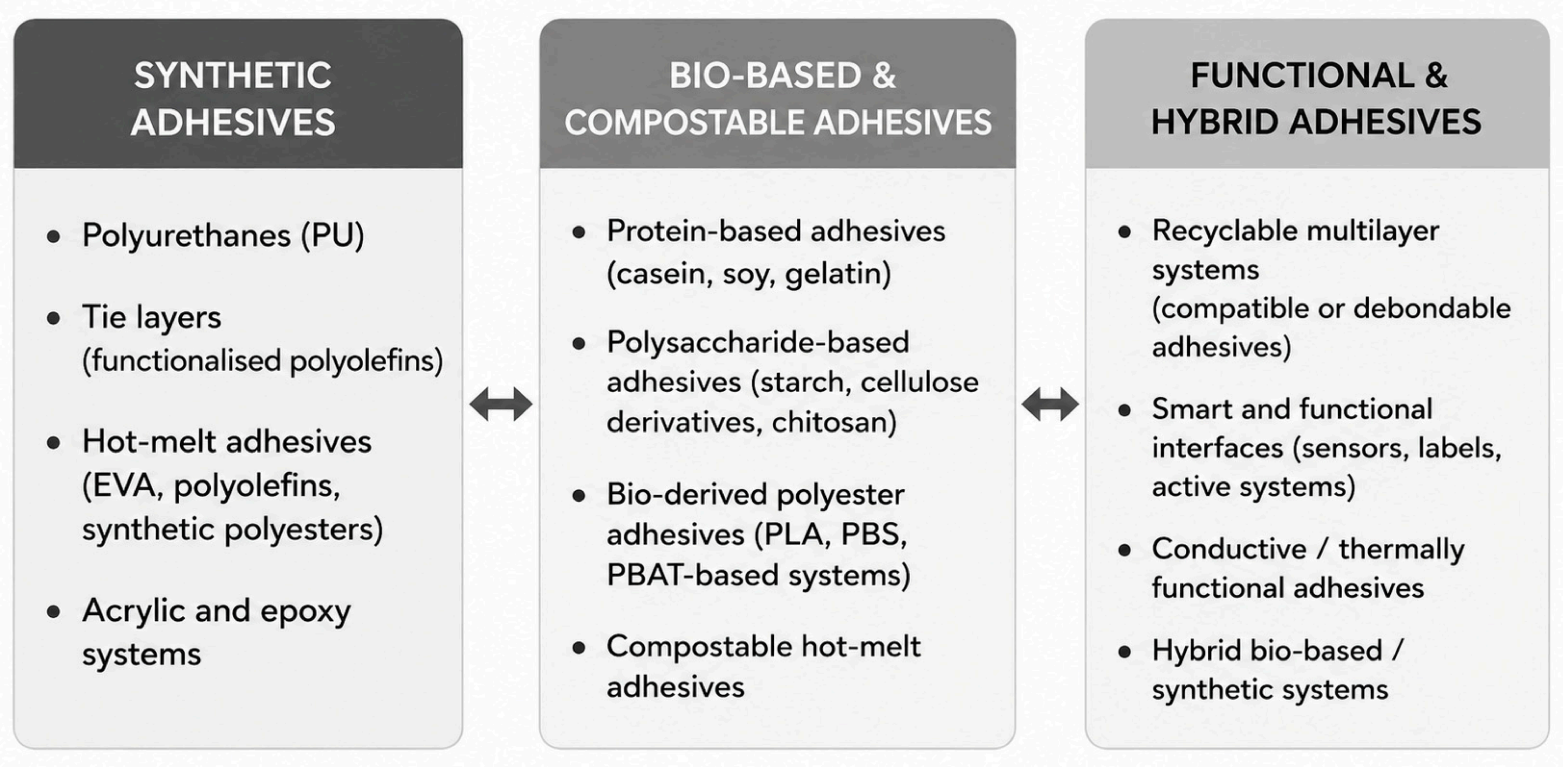
Classification of the principal adhesive material families discussed in this section, including synthetic, bio-based and compostable, and functional or hybrid adhesive systems for packaging applications.

**Figure 5:**
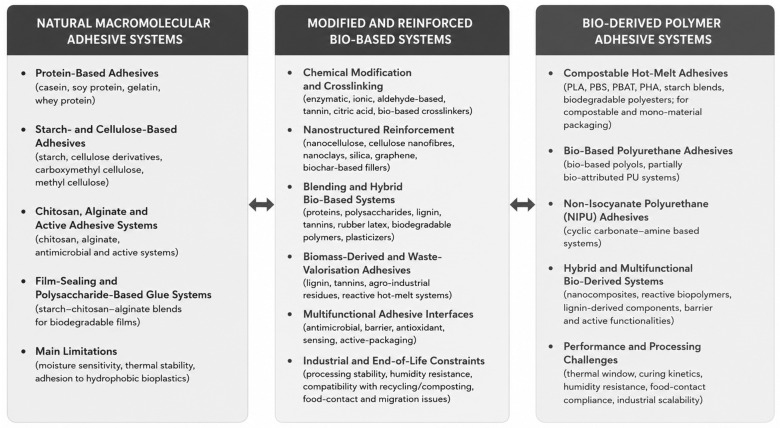
Classification of bio-based and compostable adhesives for packaging applications. Schematic overview of natural macromolecular systems, modified and reinforced bio-based systems, and bio-derived polymer systems, highlighting their main subcategories, representative formulation strategies, transition toward engineered, multifunctional, and compostable adhesive architectures for sustainable packaging.

**Figure 6:**
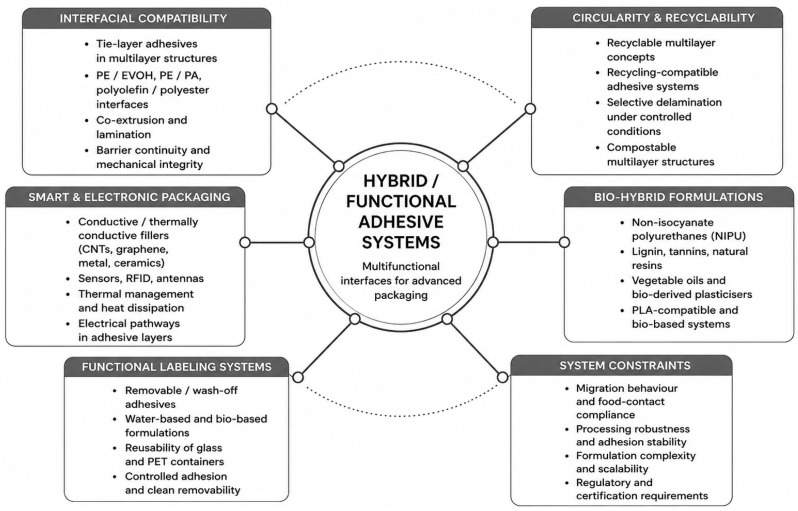
Functional roles and integration pathways of hybrid adhesive systems in advanced packaging. Schematic overview of the main functional domains of hybrid and multifunctional adhesive systems, including interfacial compatibility, smart packaging integration, bio-hybrid formulations, functional labeling systems, circularity-oriented strategies, and system-level constraints in sustainable packaging applications.

**Figure 7:**
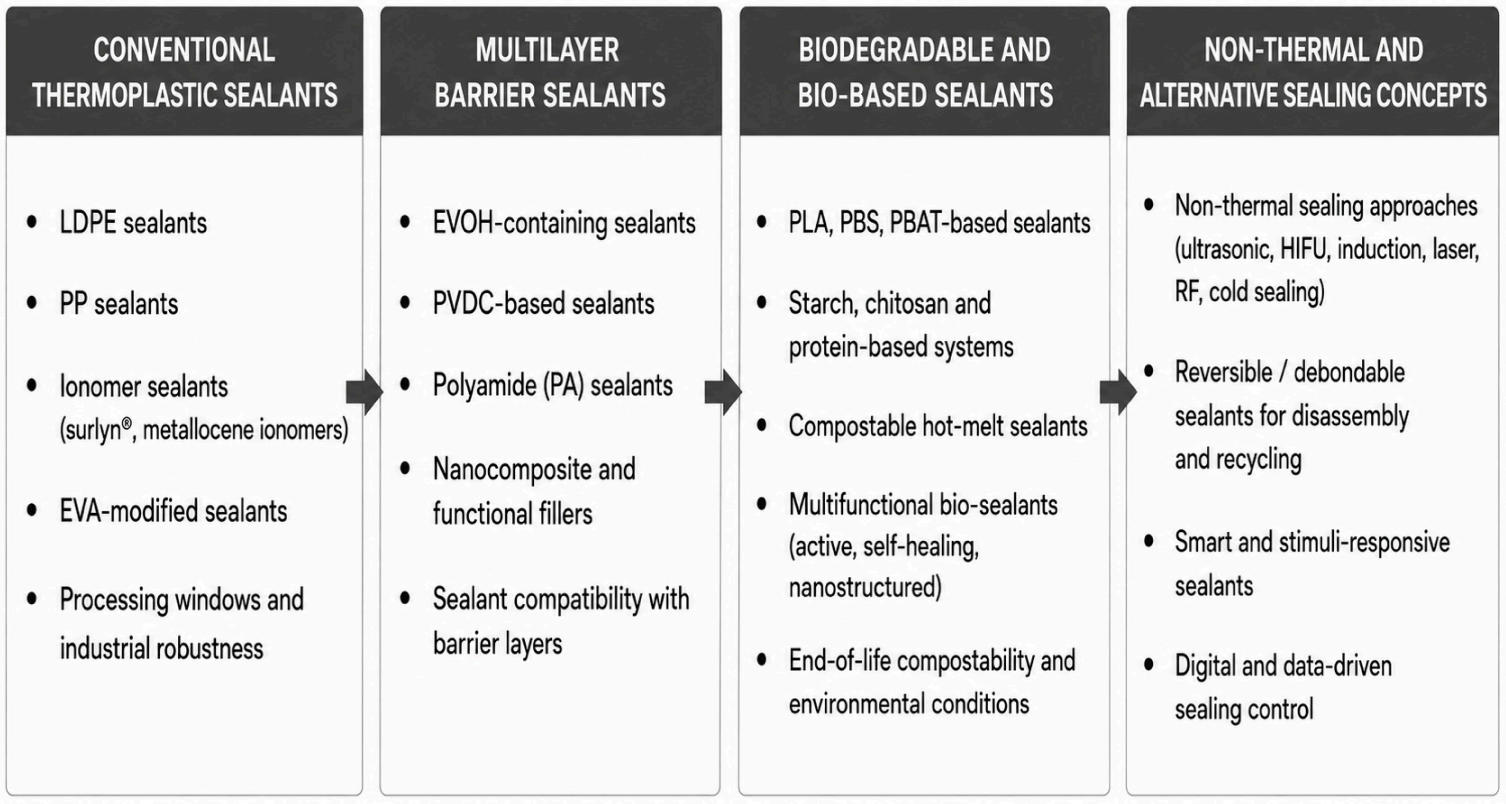
Classification framework for sealant materials in packaging applications. The figure summarises the main sealant categories discussed in Section 3, from conventional thermoplastic and multilayer barrier sealants to bio-based, biodegradable, and alternative sealing concepts, highlighting their role in seal performance, barrier continuity, recyclability, and converting compatibility.

**Figure 8:**
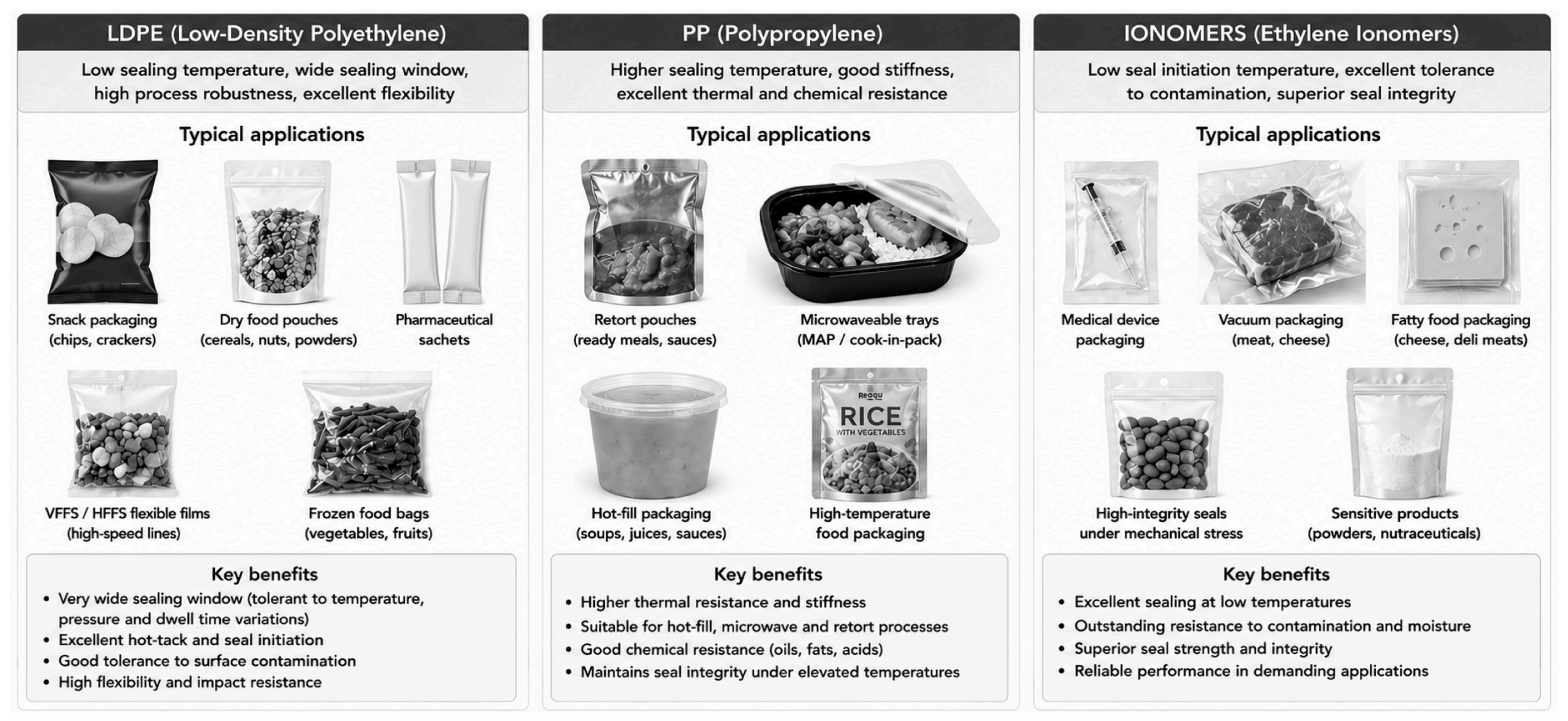
Typical industrial applications of conventional thermoplastic sealants used in heat-sealed packaging. The figure illustrates representative packaging applications and typical use scenarios associated with LDPE-, PP-, and ionomer-based sealants. In addition to common packaging formats, the comparison highlights the main functional advantages and industrial performance characteristics of each material family, including sealing-window width, thermal resistance, contamination tolerance, hot-tack behaviour, and suitability for high-speed converting operations.

**Figure 9:**
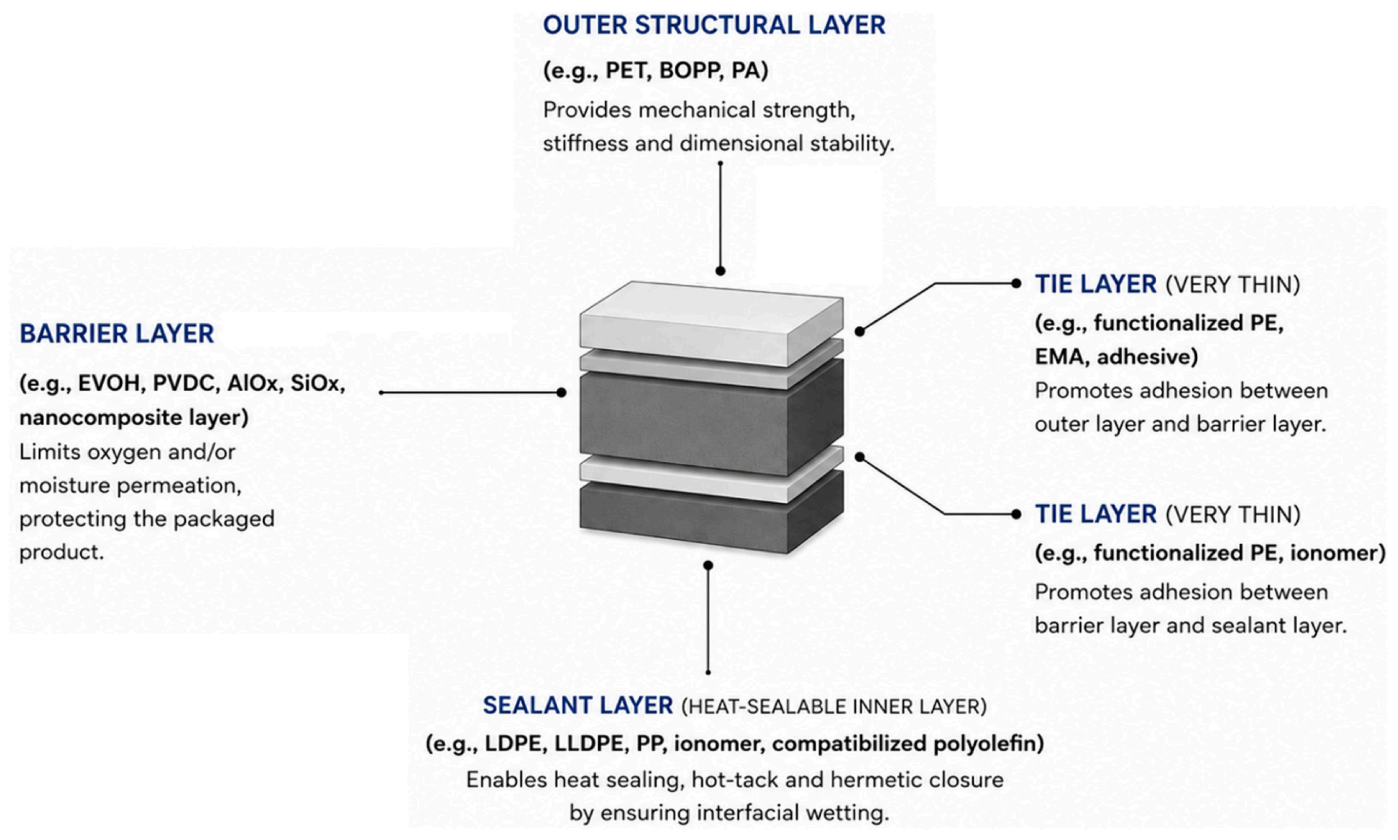
Role of the sealant layer within a multilayer barrier structure. Schematic representation of a typical multilayer barrier packaging architecture highlighting the functional role of the heat-sealable inner layer within heterogeneous multilayer systems. The figure illustrates how structural layers, barrier components, tie layers, and sealant layers cooperate to combine mechanical support, oxygen and moisture protection, interfacial adhesion, and hermetic seal formation. Particular emphasis is placed on the sealant layer as the functional interfacial element responsible for seal integrity, hot-tack performance, and barrier continuity across the sealing area.

**Figure 10:**
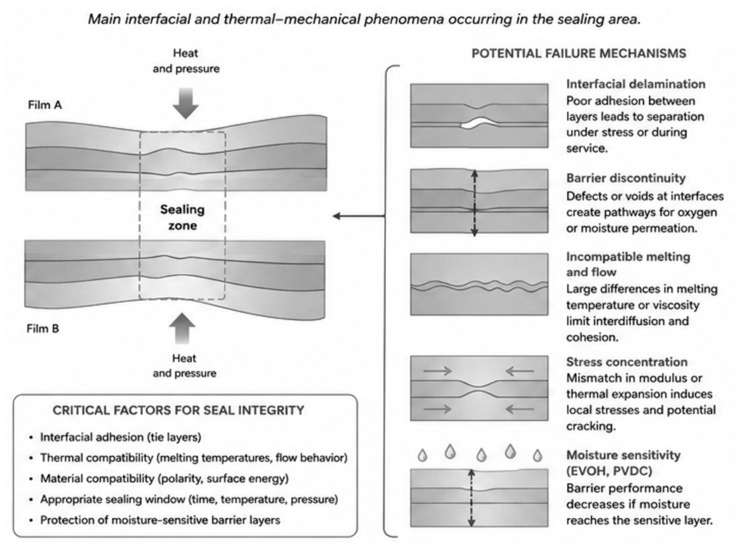
Barrier continuity and seal integrity in multilayer sealants. Schematic overview of the main interfacial and thermomechanical mechanisms affecting multilayer sealant performance, including adhesion, incompatibility, discontinuities, delamination, thermal mismatch, melting behaviour, surface polarity, and degradation under demanding service conditions.

**Figure 11:**
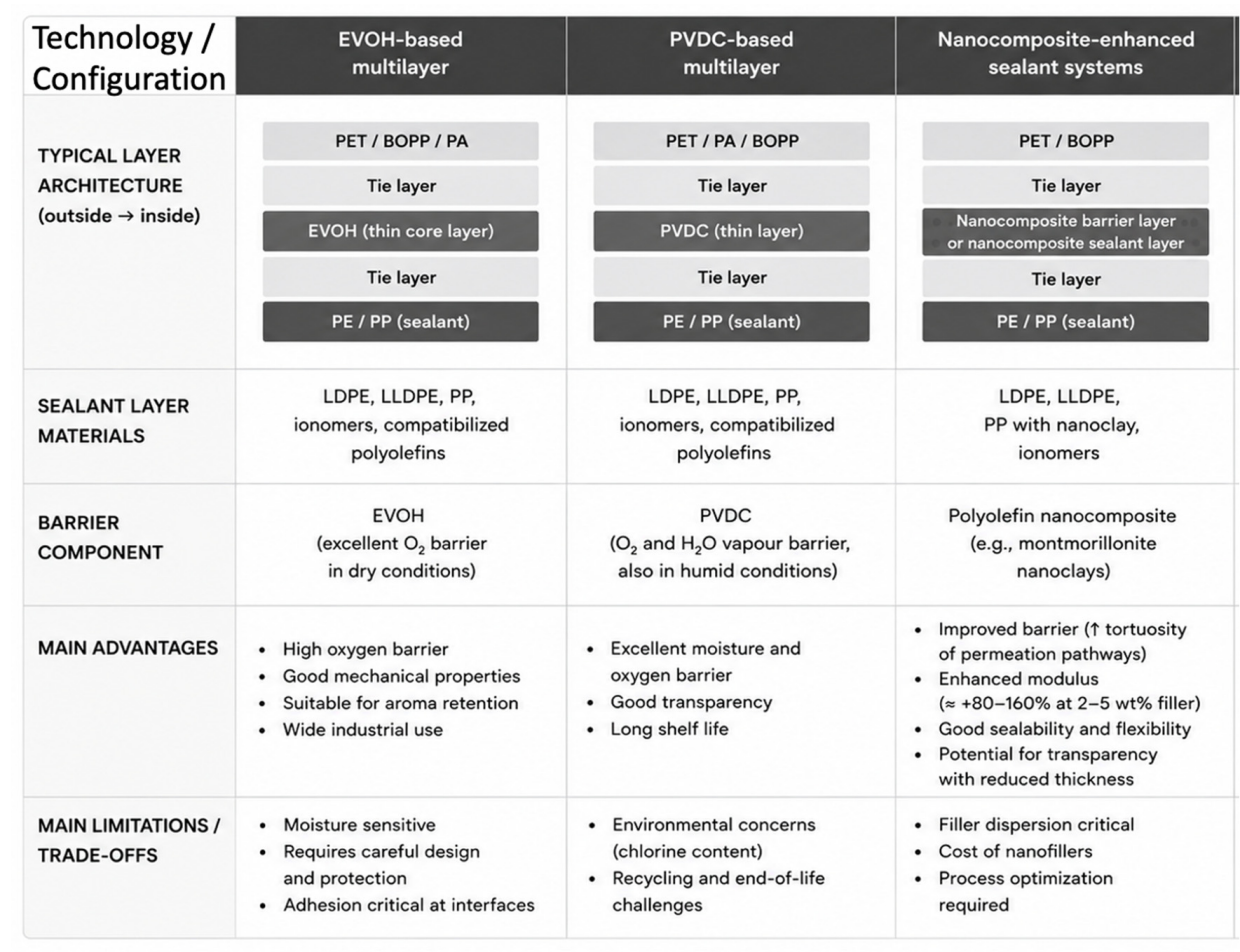
Main multilayer barrier sealant technologies. Comparative overview of representative multilayer sealant systems, summarising their architectures, sealant materials, barrier components, advantages, limitations, and main trade-offs in barrier performance, sealability, processability, and recyclability. Note: Layer sequence and materials may vary depending on product requirements, processing technology and end-use conditions.

**Figure 12:**
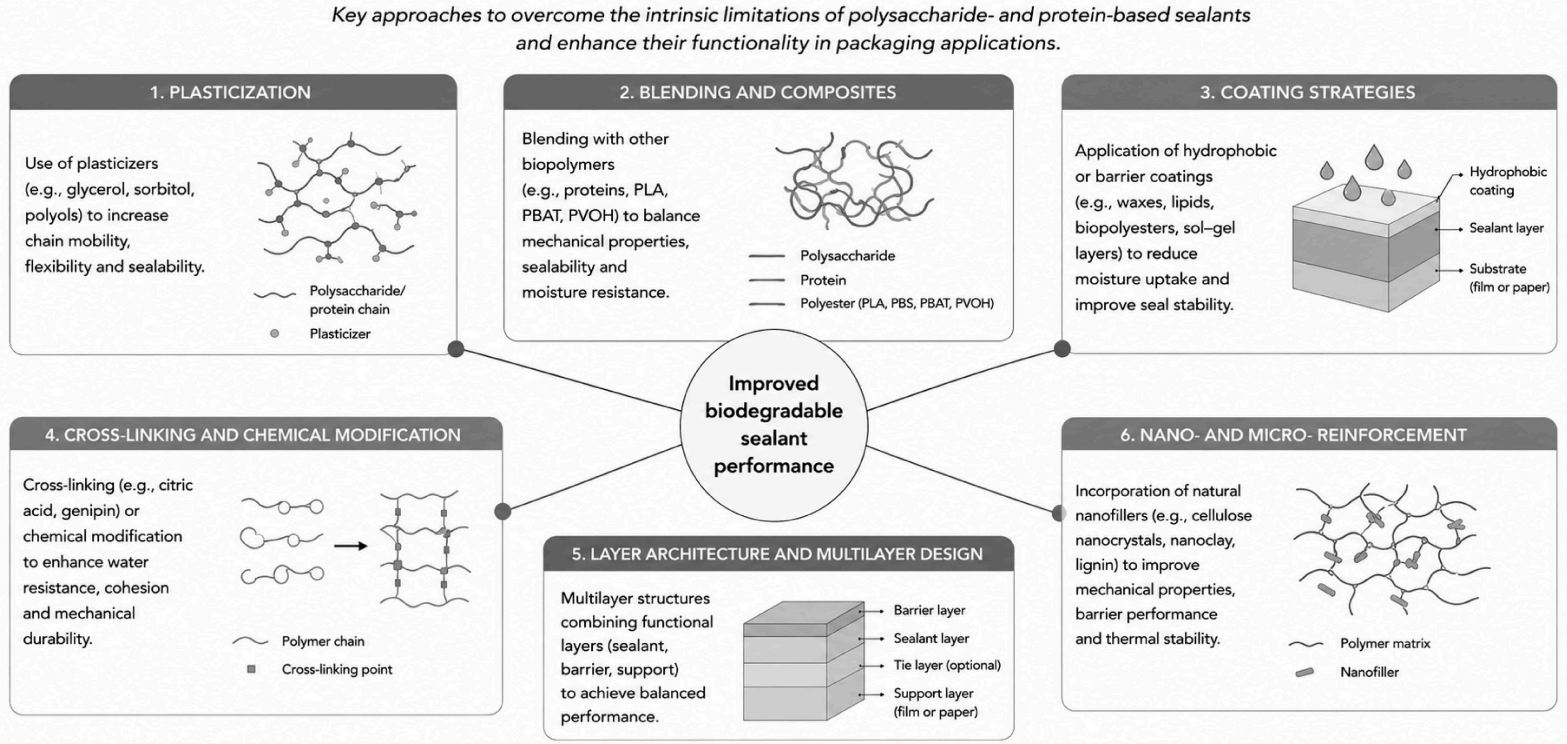
Strategies to improve biodegradable sealant performance. Schematic overview of the principal strategies currently adopted to overcome the intrinsic limitations of biodegradable and bio-based sealants in packaging applications.

**Figure 13:**
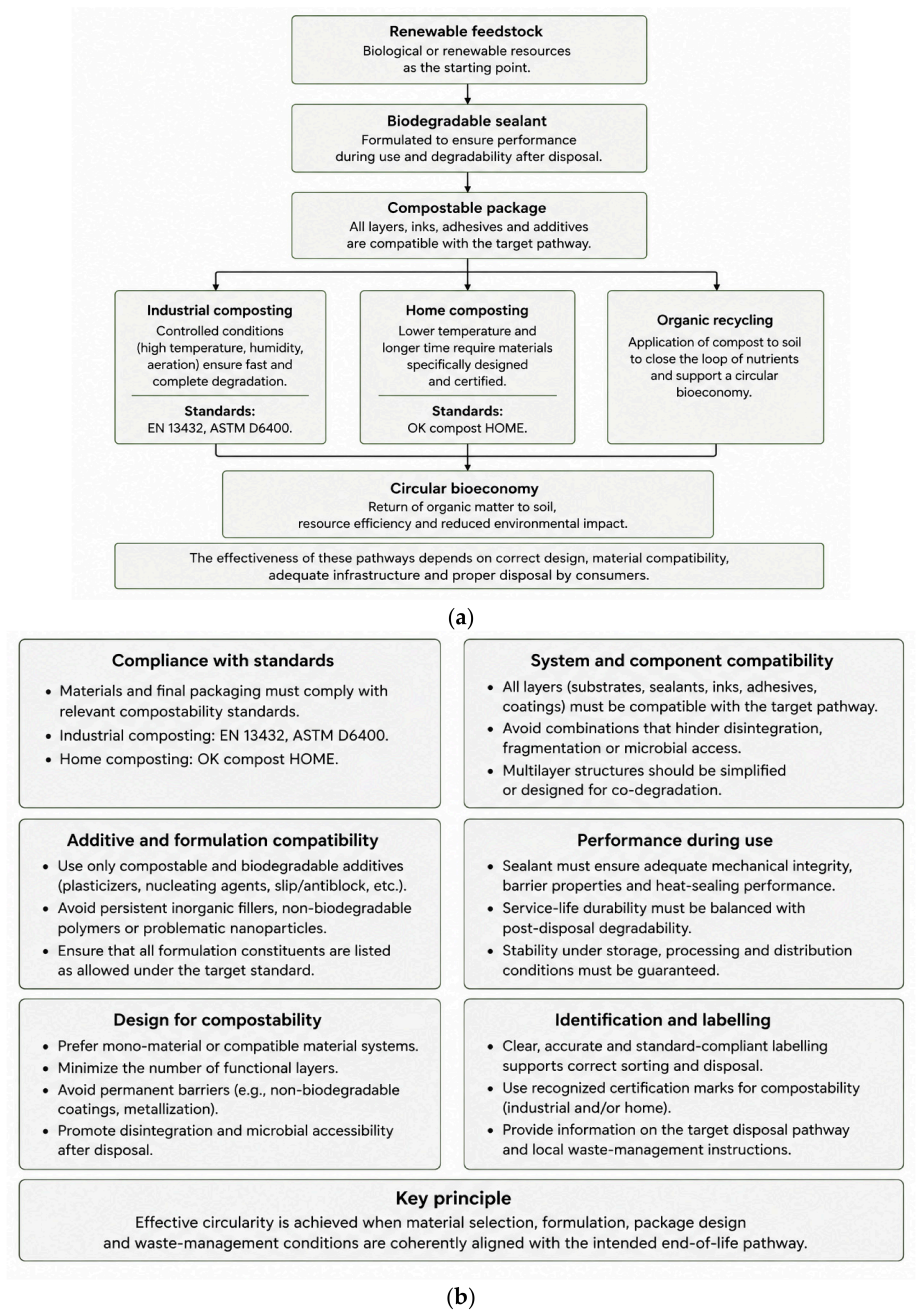

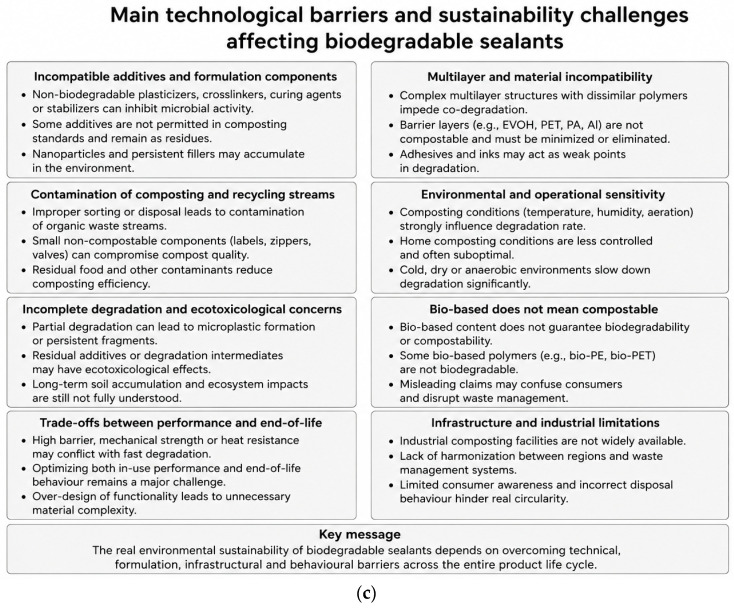
(**a**) Main end-of-life pathways for biodegradable sealants and compostable packaging systems. Conceptual overview of the principal end-of-life routes for biodegradable sealants, including industrial composting, home composting, and organic recycling pathways [9,10]. (**b**) System-level requirements for effective circularity and compostability of biodegradable sealants. Schematic representation of the main technical and regulatory requirements for the circularity of biodegradable sealants and packaging systems, including compostability compliance, multilayer compatibility, additive selection, design-for-compostability strategies, and proper waste-management labelling. (**c**) Main technological barriers and sustainability challenges affecting biodegradable sealants. Overview of the principal limitations and trade-offs affecting the real environmental sustainability of biodegradable sealing systems, including multilayer incompatibility, non-biodegradable additives, contamination of recycling streams, environmental sensitivity, incomplete degradation, and conflicts between service-life durability and end-of-life compostability or recyclability.

**Figure 14:**
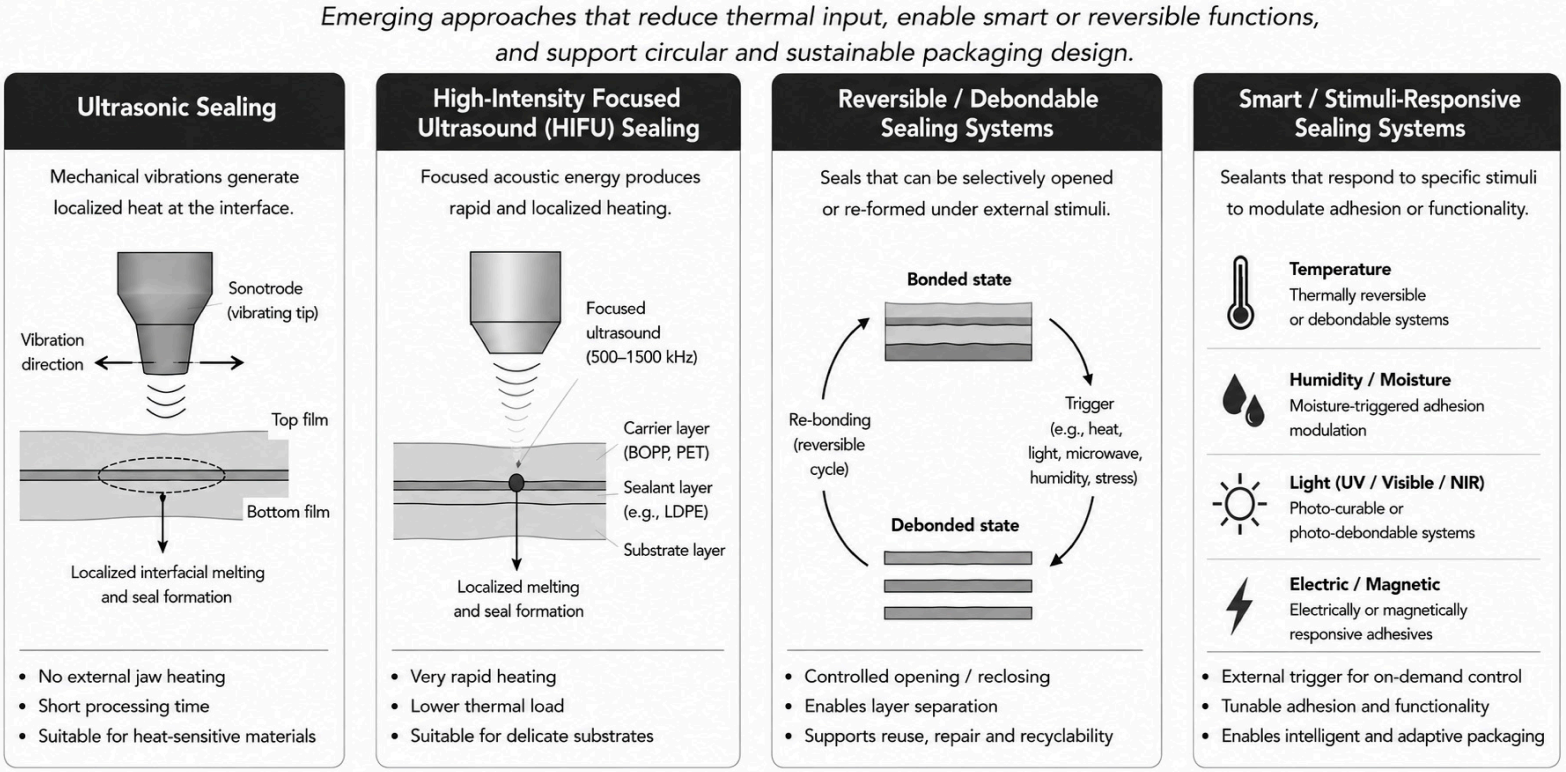
Main categories of non-thermal and alternative sealing concepts for packaging applications. Schematic overview of advanced non-thermal and alternative sealing approaches for packaging systems, including ultrasonic sealing, HIFU, reversible sealing systems, and smart stimuli-responsive sealants, highlighting their operating principles and potential benefits in terms of process flexibility, recyclability, and circular packaging.

**Figure 15:**
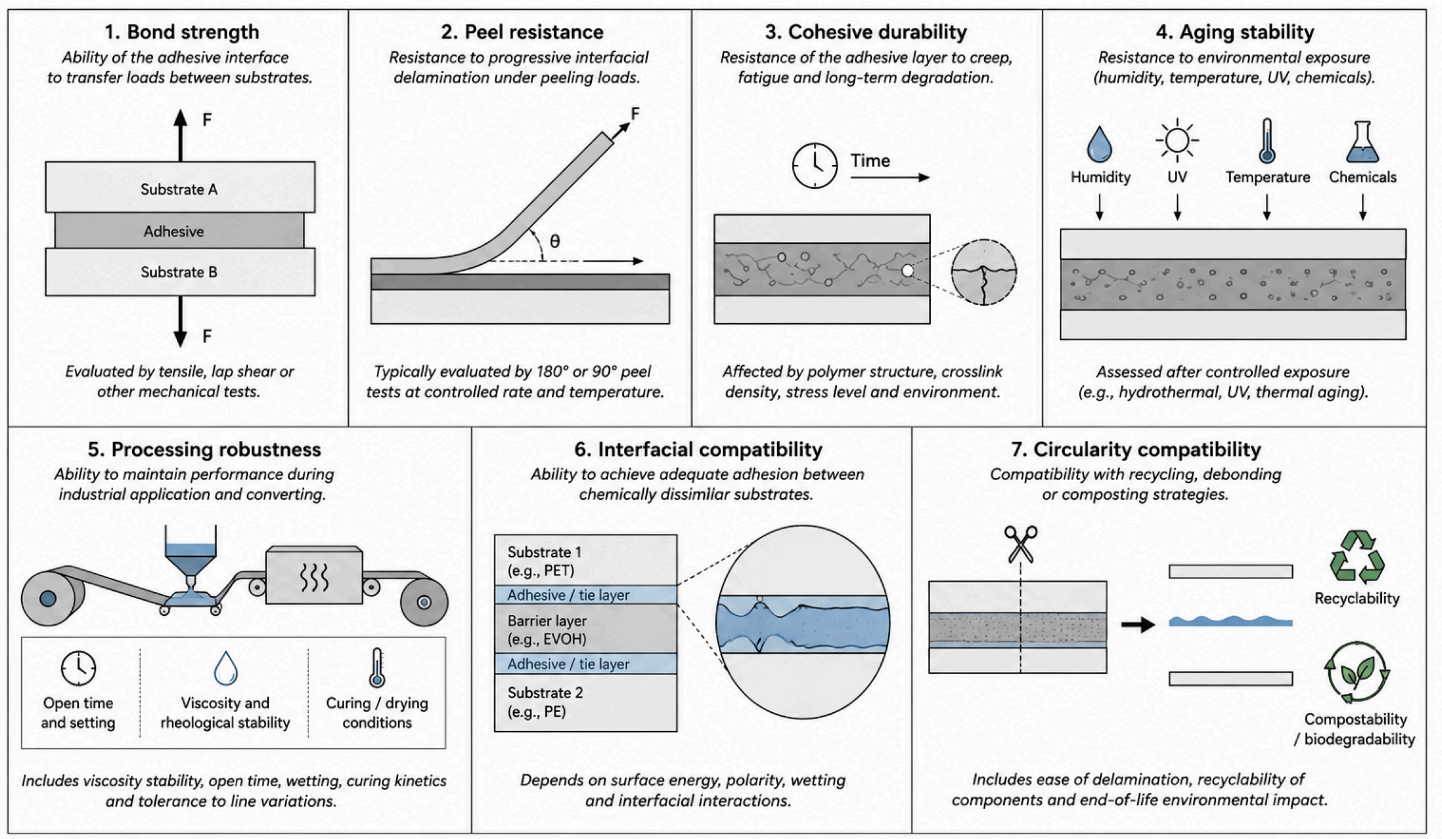
Schematic representation of the principal performance metrics used for packaging adhesives. Conceptual overview of the principal performance metrics commonly used to evaluate adhesive systems in packaging applications, including bond strength, peel resistance, cohesive durability, aging stability, processing robustness, interfacial compatibility, and circularity-related behaviour. The schematic highlights the combined influence of mechanical, environmental, and processing-related factors on adhesive reliability in multilayer, flexible, and paper-based packaging structures. Note: The metrics illustrated above are interdependent and must be evaluated in the context of the specific substrate combination, application method, service conditions and end-of-life scenario.

**Figure 16:**
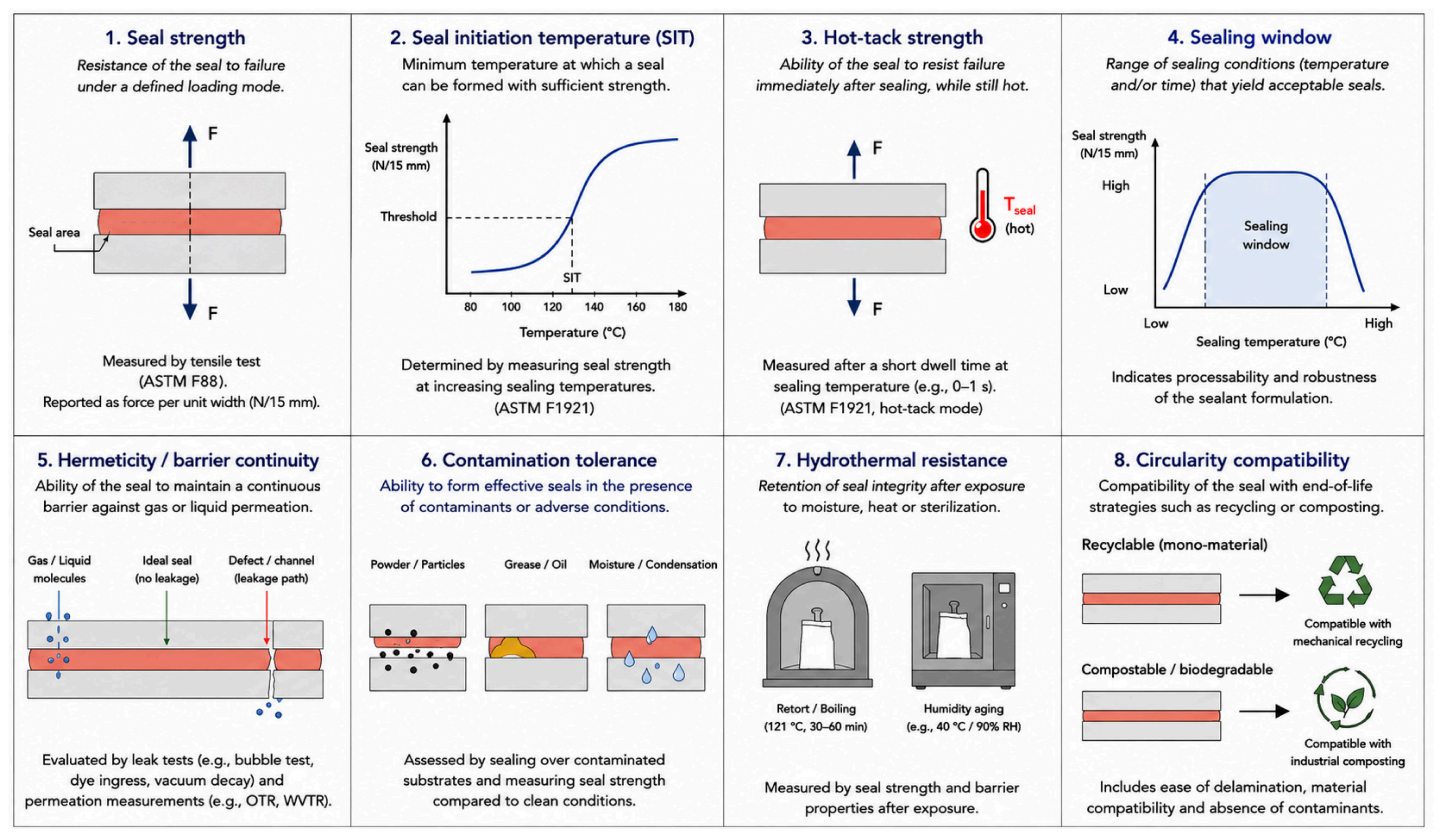
Schematic representation of the principal performance metrics used for packaging sealants. Conceptual overview of the principal performance metrics used to evaluate packaging sealants, including seal strength, SIT, hot-tack behaviour, sealing window, hermeticity, hydrothermal resistance, and circularity compatibility, highlighting their influence on sealing reliability in flexible and multilayer packaging systems. Note: Sealant performance depends on polymer chemistry, coextrusion structure, layer thickness, additives, processing conditions and packaging architecture. The metrics above should be interpreted in the context of the intended application and relevant standards [121,127].

**Figure 17:**
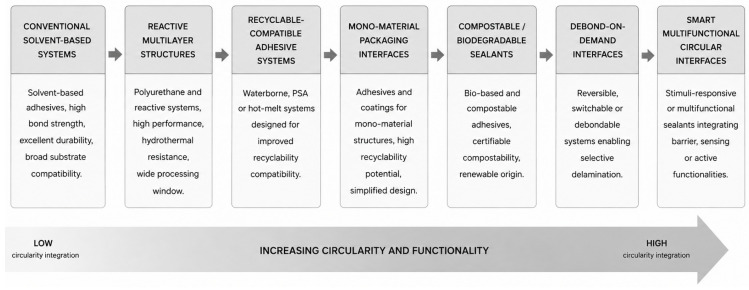
Transition pathways from conventional adhesive and sealant systems toward circular packaging interfaces. Conceptual overview of the progressive evolution of packaging adhesive and sealant technologies from conventional solvent-based and permanent multilayer systems toward recyclable mono-material architectures, compostable interfaces, debond-on-demand technologies, and smart multifunctional systems designed to support circular packaging strategies.

**Figure 18:**
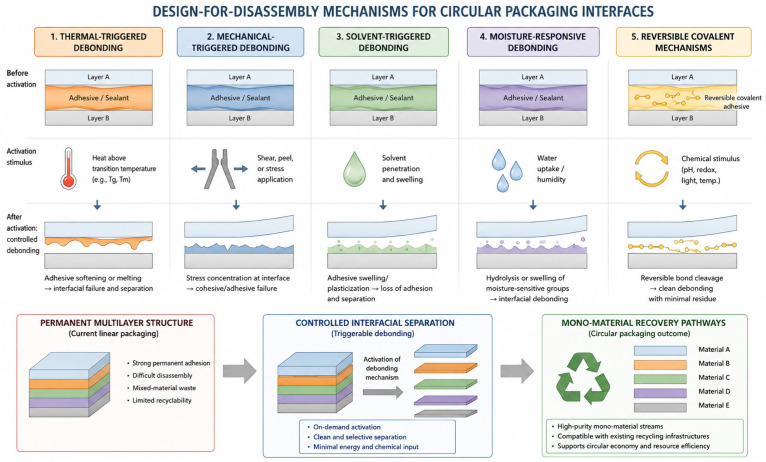
Design-for-disassembly mechanisms for circular packaging interfaces. Conceptual representation of the principal debonding strategies investigated for packaging adhesive and sealant systems, including thermal, mechanical, solvent-triggered, moisture-responsive, and reversible covalent mechanisms. The schematic illustrates the transition from permanent multilayer structures toward controlled interfacial separation and mono-material recovery pathways compatible with circular packaging systems.

**Figure 19:**
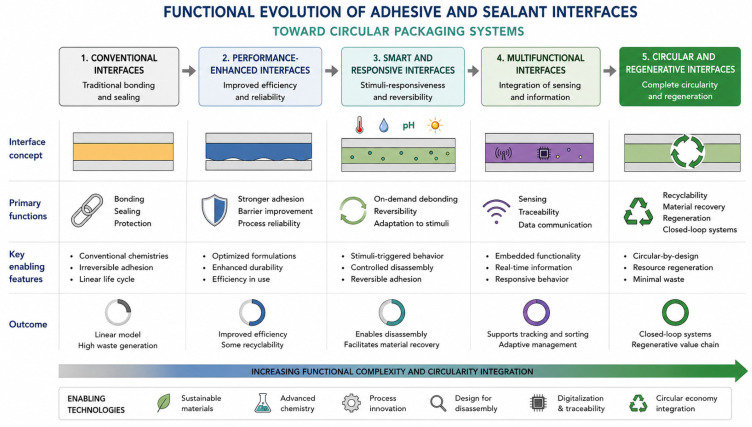
Functional evolution of adhesive and sealant interfaces toward circular packaging systems. Conceptual hierarchy illustrating the progressive evolution of packaging interfaces from conventional bonding and sealing functions toward advanced circular and multifunctional systems integrating recyclability, controlled disassembly, traceability, sensing, and adaptive interfacial behaviour. The schematic highlights the increasing functional complexity and circularity integration associated with next-generation packaging interfaces.

During the preparation of this manuscript, the authors used an AI tool (ChatGPT, GPT-4) to assist in drafting selected portions of the text. In addition, AI assistance was used in the preparation of the figures as follows: Figure 1, Figure 2, Figure 4 and Figure 5 were originally created by the authors using Microsoft PowerPoint, and the AI tool was subsequently used only to enhance their visual/aesthetic appearance. Figures 6–19 were generated with the assistance of the AI tool, based on scientific content, data, and specifications entirely defined by the authors. In all cases, the AI-assisted outputs were reviewed, revised, and validated by the authors to ensure scientific accuracy and consistency with the analytical framework of the review. The use of this tool did not involve the processing or analysis of primary research data, and full editorial responsibility for all content remains with the authors.

## 2. Adhesive Materials for Packaging Applications

Adhesive materials used in packaging encompass a broad range of synthetic, bio-based, compostable, and multifunctional systems designed to satisfy increasingly complex requirements related to bonding performance, converting compatibility, barrier integrity, regulatory compliance, and end-of-life management. As packaging architectures evolve toward multilayer, lightweight, recyclable, and compostable configurations, adhesive materials can no longer be considered simple joining agents, but rather interfacial technologies that directly influence the mechanical, thermal, and environmental behaviour of the entire packaging system [14,15].

From a technological perspective, adhesive systems for packaging can be broadly classified into three main groups: conventional synthetic adhesives, bio-based and compostable systems, and functional or hybrid adhesives incorporating additional interfacial functionalities. Synthetic systems remain dominant in high-performance flexible and multilayer packaging due to their broad processing windows, strong adhesion, and durability under demanding industrial conditions. In parallel, increasing sustainability requirements have accelerated the development of bio-based and compostable adhesives designed to improve renewable content, compostability, and compatibility with circular packaging strategies. More recently, hybrid and multifunctional systems have emerged to address advanced requirements such as recyclable multilayers, controlled delamination, thermal management, active packaging, and smart interfaces [4,8].

The following sections analyse these adhesive families through a performance- and application-oriented perspective, with particular emphasis on interfacial behaviour, processing constraints, quantitative performance metrics, and compatibility with emerging sustainability and recycling requirements. Rather than treating adhesive materials as isolated chemistries, the discussion considers their role within complete packaging architectures, where bonding performance, converting behaviour, barrier continuity, and end-of-life compatibility are strongly interconnected.

Figure 4 summarises the main adhesive material families discussed in this section and highlights the cross-cutting aspects linking interfacial behaviour, processing compatibility, performance, and end-of-life management within packaging systems.

### 2.1. Synthetic Adhesives

Synthetic adhesives remain the technological backbone of contemporary packaging systems, particularly in multilayer structures and demanding applications in the food, pharmaceutical, and technical sectors. Their continued industrial relevance derives from the combination of high bonding reliability, broad processing compatibility, mechanical durability, and the ability to maintain laminate integrity under severe converting and service conditions.

From a technological perspective, the main synthetic adhesive families considered in this section include polyurethane-based laminating adhesives, modified polyolefin tie layers, acrylic and waterborne systems, and thermoplastic hot-melt adhesives. These materials differ in chemistry, activation mechanism, rheology, curing or solidification behaviour, thermal resistance, and substrate compatibility. Their selection is therefore governed not only by nominal bond strength, but also by processing route, curing or setting kinetics, application temperature, food-contact requirements, resistance to humidity or thermal stress, and compatibility with the specific multilayer architecture in which they are incorporated [14,16].

**Polyurethane-based adhesives**

Among synthetic systems, polyurethane (PU)-based adhesives represent the most widely adopted class for flexible and multilayer packaging, especially in the form of two-component reactive laminating systems. Their industrial importance derives from the possibility of tailoring molecular architecture, curing kinetics, flexibility, cohesive strength, and interfacial polarity to bond chemically dissimilar substrates such as polyolefins, polyesters, polyamides, aluminum foils, paper, and high-barrier polymers [16,17]. PU-based laminating adhesives are particularly important in retortable and high-performance flexible packaging because they combine strong interfacial adhesion with hydrothermal stability, wettability, and mechanical durability under demanding service conditions.

Polyurethane adhesives also play a structural role within multilayer laminates. The overall performance of multilayer packaging systems depends not only on the intrinsic properties of individual films, but also on the stability of the adhesive interlayer and its influence on interfacial interactions between adjacent layers. Their elastomeric character helps accommodate differential thermal expansion, local stress concentrations, and mechanical deformation during converting, sealing, transport, and service, thereby reducing the risk of interfacial cracking or delamination. In multilayer flexible packaging, laminate durability is therefore strongly influenced by the cohesive strength, stress-relaxation capability, aging resistance, and hydrothermal stability of the adhesive layer itself.

Recent research has also shown that polyurethane adhesive performance is closely associated with crosslink density, phase morphology, and microphase separation phenomena within the polymer network. Crosslinked polyurethane systems generally exhibit improved cooking resistance, peel strength, and hydrothermal stability because the interconnected polymer structure limits moisture penetration and enhances cohesive integrity under high-temperature and high-humidity conditions [17]. In addition, hybrid polyurethane/acrylate systems and waterborne polyurethane dispersions have attracted increasing attention because they combine improved flexibility, surface wetting capability, and reduced volatile organic compound (VOC) emissions with acceptable adhesion performance in flexible packaging applications [18,19].

Despite their high performance, polyurethane adhesives raise environmental and occupational concerns associated with isocyanate-containing formulations. The 2020 update to Annex XVII of the REACH Regulation introduced mandatory training requirements for professional and industrial users handling diisocyanate-containing products above 0.1%, with enforcement from August 2023 [20]. These restrictions have accelerated research into low-monomer systems, solvent-free technologies, bio-attributed polyurethane formulations, and non-isocyanate polyurethanes (NIPUs). NIPU systems are particularly attractive because they avoid isocyanates, but their industrial implementation remains limited by slower polymerization kinetics, long curing times, and performance gaps compared with conventional PU laminating systems [21,22].

Long-term durability and environmental aging also remain important considerations for polyurethane-based systems. Hydrothermal exposure, oxygen diffusion, and thermo-oxidative degradation may progressively alter urethane linkages and reduce interfacial stability, particularly under severe environmental conditions. Consequently, current research increasingly focuses on improving aging resistance, hydrothermal durability, and recyclability while maintaining the high adhesion performance required for multilayer packaging applications.

**Modified polyolefin tie layers**

Modified polyolefin adhesives are extensively used as tie layers in co-extruded multilayer packaging systems, where they promote adhesion between otherwise incompatible polymers. Conventional polyethylene and polypropylene exhibit low polarity and therefore show poor interfacial adhesion toward polar substrates such as ethylene vinyl alcohol (EVOH), polyamide (PA), or polyethylene terephthalate (PET). Functionalization through maleic anhydride grafting, graft copolymer formation, or related reactive compatibilization strategies is therefore widely employed to improve interfacial bonding and stabilize multilayer structures [23].

Tie layers play a critical role in multilayer barrier packaging because they enable the integration of high-barrier polymers within polyolefin-rich architectures while maintaining interlayer cohesion, thermoformability, puncture resistance, and mechanical integrity during converting and service conditions. In many flexible food packaging structures, modified polyolefins based on maleated polyethylene or maleated linear low-density polyethylene (LLDPE-g-MA) are positioned between EVOH barrier layers and external polyolefin layers to ensure adequate stress transfer and adhesion stability under thermal and mechanical loading [23]. Multilayer systems containing EVOH core layers and maleic-anhydride-modified tie layers are widely used in food packaging because they combine the moisture resistance and processability of polyolefins with the superior oxygen barrier properties of EVOH [23,24].

Ref. [25] further showed that anhydride- and acid-modified polyolefins strongly influence the interfacial adhesion performance of multilayer barrier films through chemical interactions at polymer interfaces. The effectiveness of these tie layers is therefore closely related to interfacial chemistry, layer architecture, and co-extrusion processing conditions.

At the same time, tie layers illustrate one of the main performance–circularity trade-offs associated with advanced multilayer packaging. Although they enable the production of high-performance barrier structures with improved shelf-life and mechanical durability, strongly bonded multilayer architectures can complicate mechanical recycling because the separation of incompatible polymer phases becomes difficult during reprocessing [4]. Recent design-for-recycling strategies have therefore focused on reducing material complexity, developing recyclable polyolefin-rich multilayer systems, and optimizing EVOH and tie-layer content to improve compatibility with existing recycling streams [24].

**Acrylic and waterborne adhesives**

Acrylic and waterborne adhesive systems are widely used in packaging applications requiring transparency, coating compatibility, low VOC emissions, or controlled adhesion behaviour. Acrylic adhesives are especially relevant for pressure-sensitive labels, tapes, resealable structures, and coated flexible substrates because of their transparency, viscoelastic tunability, formulation versatility, and compatibility with coating and printing technologies [2,18]. Waterborne acrylic emulsions, vinyl-acetate-based systems, acrylic dispersions, polyurethane dispersions (PUDs), and hybrid polyurethane/acrylic systems have been extensively investigated for flexible packaging, lamination, coatings, and paper-based packaging applications, particularly in response to increasing environmental restrictions associated with solvent-based technologies and VOC emissions [16,19,26].

In pressure-sensitive packaging applications, acrylic-based systems are particularly valued for their rapid adhesion under low applied pressure, optical clarity, resistance to oxidation and ultraviolet exposure, and stability during aging [18]. These characteristics make them suitable for labels, protective films, tapes, and resealable packaging structures, where balanced tack, peel strength, and shear resistance are required. Hybrid polyurethane–acrylate systems have also attracted increasing attention because they combine the flexibility, transparency, and surface wetting capability of acrylics with the toughness, film-forming ability, and chemical resistance of polyurethane phases [18].

Waterborne systems are additionally important in paper and paperboard packaging, where compatibility with fibrous substrates, coating integration, and reduction in solvent emissions represent important design requirements. In these systems, water acts as the primary dispersion medium, reducing environmental impact and improving handling safety compared with conventional solvent borne adhesives [19,26]. Several studies have therefore focused on waterborne polyurethane/acrylic dispersions and emulsion-polymerized hybrid systems designed for food packaging laminates and flexible multilayer structures [19].

Compared with reactive polyurethane adhesives, acrylic and waterborne systems generally exhibit lower thermal resistance, lower moisture resistance, and reduced durability under severe sterilization or retort conditions, which may limit their use in demanding multilayer food packaging applications [26]. Waterborne polyurethane systems may also show insufficient adhesion on low-surface-energy polymer films and lower mechanical resistance if not properly modified through crosslinking, acrylic hybridization, or nanoparticle incorporation [18,26]. Nevertheless, these adhesive systems remain highly relevant for lower-temperature packaging operations, pressure-sensitive applications, recyclable paper-based structures, and packaging concepts aimed at reducing adhesive contamination during recycling processes or improving compatibility with more sustainable packaging strategies [2,19].

**Hot-melt adhesives**

Hot-melt adhesives (HMAs) constitute one of the most important adhesive technologies in industrial packaging because they combine rapid processing, solvent-free application, and compatibility with high-speed converting operations. Their industrial relevance derives not only from fast solidification kinetics, but also from the possibility of tailoring rheological behaviour, wetting characteristics, and cohesive performance through complex multicomponent formulations [27,28].

Commercial HMAs are typically based on ethylene-vinyl acetate (EVA), polyolefins, polyesters, polyamides, polyurethane-based systems, or synthetic rubbers combined with tackifying resins, waxes, plasticizers, antioxidants, and stabilizing additives. In these formulations, the polymer matrix primarily governs cohesive strength and viscoelastic behaviour, tackifiers promote substrate wetting and interfacial adhesion, while waxes strongly influence melt viscosity, crystallization kinetics, setting speed, and processability [28]. The balance between these constituents determines not only adhesion strength, but also open time, flow behaviour, solidification rate, and mechanical stability during packaging operations.

From a mechanical perspective, HMA performance in packaging applications is strongly associated with viscoelastic dissipation and interfacial fracture behaviour. Recent studies on adhesive joints for flexible packaging have shown that failure mechanisms frequently involve nonlinear elastic-plastic deformation within the adhesive layer, progressive interfacial damage, and localized plasticity phenomena during peel-induced delamination [29]. Consequently, package reliability depends not only on nominal adhesion strength, but also on cohesive integrity, stress-relaxation capability, crack-propagation resistance, and energy dissipation during opening and handling operations [29].

Because bonding is primarily governed by thermoplastic solidification rather than permanent chemical crosslinking, conventional HMAs remain intrinsically sensitive to temperature. Exposure to elevated temperatures may reduce cohesive integrity through softening, creep, or accelerated interfacial deformation, limiting the applicability of standard formulations in retortable, sterilizable, or high-temperature packaging systems [27]. For this reason, current developments increasingly focus on modified or hybrid formulations capable of improving thermal resistance while preserving rapid processing capability.

In parallel, food-contact packaging applications have raised increasing attention toward migration phenomena associated with tackifiers, waxes, mineral-oil-derived fractions, residual monomers, and non-intentionally added substances (NIAS) originating from adhesive formulations [30]. The compositional complexity of HMAs therefore creates additional challenges related to toxicological assessment, migration control, and regulatory compliance in multilayer food packaging systems.

Recent developments increasingly focus on sustainability-oriented HMA technologies, including formulations incorporating recycled or upcycled wax fractions derived from mixed polyolefin waste streams [28], as well as supramolecular and reusable adhesive architectures based on reversible hydrogen-bonding interactions and microphase-separated structures [31]. These approaches aim to improve circularity, reusability, and low-temperature adhesion performance while maintaining compatibility with industrial packaging processes.

### 2.2. Bio-Based and Compostable Adhesives

Bio-based and compostable adhesives have emerged as a major research and development area in response to increasing sustainability requirements, circular-economy targets, and growing demand for packaging systems with improved end-of-life compatibility [32]. Although conventional synthetic adhesives remain essential in many high-performance packaging applications because of their processing versatility, durability, and interfacial reliability, growing efforts are directed toward the development of adhesive systems capable of integrating renewable feedstocks, reduced environmental impact, compostability, or improved compatibility with circular packaging strategies [8,14,16,32].

From a materials perspective, bio-based packaging adhesives can be organised into three main groups: natural macromolecular systems, modified or reinforced bio-based formulations, and bio-derived or bio-attributed polymer systems, including compostable hot-melts, bio-based polyurethanes, and non-isocyanate polyurethanes (NIPUs). These families differ in adhesion mechanism, moisture sensitivity, thermal stability, rheological behaviour, and processing compatibility. For this reason, current research increasingly focuses on improving their performance through targeted modification, crosslinking, reinforcement, and hybridisation strategies, while preserving renewable content, compostability where required, and compatibility with industrial packaging-converting operations [32,33,34].

As schematically illustrated in Figure 5, the development of bio-based adhesive technologies increasingly follows a transition from conventional natural macromolecules toward more engineered and multifunctional systems capable of balancing adhesion performance, processability, moisture resistance, thermal stability, and end-of-life requirements. The following subsections therefore discuss the main categories of bio-based and compostable adhesives currently investigated for packaging applications, with particular attention to formulation strategies, structure–property relationships, industrial applicability, and current performance limitations.

#### 2.2.1. Protein- and Polysaccharide-Based Adhesives

Protein- and polysaccharide-based adhesives represent one of the most established groups of bio-based adhesive systems investigated for packaging applications. These materials are derived from naturally occurring macromolecules containing polar functional groups capable of promoting adhesion through hydrogen bonding, electrostatic interactions, and mechanical interlocking with fibrous or polar substrates. Their renewable origin, biodegradability, and compatibility with paper-based materials have stimulated renewed interest in sustainable packaging applications, particularly labels, corrugated board, paper laminates, coatings, and biodegradable packaging systems [14,15,32,33].

**Protein-based systems**

Protein-based adhesives, including casein, soy protein, gelatin, and whey-protein-derived systems, have historically played an important role in packaging and labeling applications because of their ability to adhere effectively to cellulose-rich substrates and polar surfaces. Casein adhesives remain particularly relevant in labeling systems for reusable glass and PET containers, where controlled removability during industrial washing operations is required. Ref. [35] reported water-based casein formulations capable of maintaining adhesion while facilitating label removal during recycling and reuse operations. These systems are generally applied at room temperature or under mild heating conditions and can avoid solvent-related VOC emissions, making them attractive for food-related and recyclable packaging applications.

Soy-protein-based systems have also attracted increasing attention as partially renewable alternatives for paper- and fibre-based packaging applications, especially when combined with crosslinking or reinforcing strategies aimed at improving water resistance and mechanical stability [32,33,36,37]. However, their direct use remains limited by moisture sensitivity, biological variability, and lower durability compared with synthetic systems, which explains the growing interest in modified protein networks and hybrid formulations.

**Starch- and cellulose-based systems**

Polysaccharide-based adhesives mainly include starch, cellulose derivatives, chitosan, alginate, and related systems. Among them, starch-based adhesives retain a central role in corrugated board and paper packaging because of their low cost, abundant availability, environmental compatibility, and strong affinity for cellulosic substrates [32,38]. Native starches adhere primarily through hydrogen bonding and mechanical interlocking, but their adhesive strength and water resistance can be improved through chemical grafting, crosslinking with organic acids, or formation of polyelectrolyte complexes. Ref. [39] demonstrated that starch-based polyelectrolyte complex adhesives markedly improved water resistance and bonding strength on paper substrates while maintaining adequate adhesion performance for fibre-based substrates.

Cellulose derivatives such as carboxymethyl cellulose and methyl cellulose are frequently employed as rheology modifiers, coating agents, or film-forming components in paper-based packaging systems because of their compatibility with fibrous substrates and their ability to generate continuous hydrogen-bonded networks [34,38]. In adhesive formulations, cellulose-based components can contribute to viscosity control, cohesive strength, and film formation, although excessive hydrophilicity may increase humidity sensitivity and limit performance in wet environments.

**Chitosan, alginate, and active adhesive systems**

Chitosan- and alginate-based systems have received growing attention because of their film-forming capability, intrinsic antimicrobial activity, and compatibility with active-packaging concepts. Chitosan-based materials are increasingly investigated for coatings, biodegradable films, and multifunctional adhesive formulations because of their ability to combine adhesion, antimicrobial behaviour, and barrier functionality [34,40]. In particular, chitosan can interact with fibre-based substrates through hydrogen bonding and electrostatic interactions, while its cationic character supports antimicrobial activity and compatibility with polyelectrolyte complexation strategies.

Ref. [34] reported that blending chitosan with tannic acid or shellac improved adhesive performance on fibre-based substrates: chitosan–tannin and shellac–chitosan formulations showed improved tensile performance compared with commercial adhesive references, together with favourable penetration and cohesive behaviour on rough paper-based materials. These results are relevant because they show that chitosan-based systems can move beyond simple bio-based adhesion toward multifunctional interfacial layers combining bonding, coating, and active-packaging functions.

Alginate-containing systems are also relevant for biodegradable film sealing and waterborne adhesive formulations. Recent work shows increasing interest in adhesive systems specifically developed for sealing biodegradable and polysaccharide-based films, which often exhibit limited heat-sealing capability because of their non-thermoplastic behaviour. Ref. [41] developed a sodium alginate–chitosan–starch-based adhesive formulation capable of sealing biopolymer films while maintaining measurable adhesion strength even under high-humidity conditions, highlighting the growing relevance of bio-based glue systems for biodegradable packaging structures.

**Main limitations of natural macromolecular adhesives**

Despite these advantages, protein- and polysaccharide-based adhesives generally exhibit important limitations associated with moisture sensitivity, limited water resistance, reduced thermal stability, microbial susceptibility, and lower long-term durability compared with conventional synthetic systems [32,42,43]. Their performance may vary significantly depending on humidity, pH, drying conditions, substrate morphology, and storage conditions, which can complicate industrial processing and long-term reliability.

Further limitation concerns adhesion to hydrophobic polymeric substrates, including PLA, PBAT, and other biodegradable polyesters used in flexible and multilayer compostable packaging. Many natural macromolecular adhesives are highly polar and therefore interact more effectively with paper, board, and cellulose-rich materials than with hydrophobic bioplastic films [44]. This mismatch between adhesive polarity and substrate surface chemistry remains one of the main barriers to the direct use of native bio-based adhesives in flexible multilayer packaging systems. Consequently, current research increasingly focuses on chemical modification, crosslinking, blending, and reinforcement strategies aimed at enhancing cohesive strength, water resistance, thermal stability, and compatibility with modern packaging-converting operations.

#### 2.2.2. Modified and Reinforced Bio-Based Adhesive Systems

To overcome the intrinsic limitations of natural bio-based adhesives, recent research increasingly focuses on modification and reinforcement strategies capable of improving water resistance, thermal stability, cohesive strength, rheological behaviour, and long-term durability while preserving renewable content and, where required, biodegradability. These approaches include chemical functionalisation, crosslinking, polymer blending, nanostructuring, and incorporation of reinforcing fillers or multifunctional additives [32,33,34].

Crosslinking represents one of the most widely adopted approaches for improving the performance of protein- and polysaccharide-based adhesives. By increasing intermolecular interactions and reducing polymer-chain mobility, crosslinking can enhance water resistance, cohesive strength, and dimensional stability [33]. Various crosslinking approaches have been investigated, including enzymatic treatments, ionic crosslinking, aldehyde-based reactions, tannin chemistry, citric acid modification, and bio-based multifunctional crosslinkers [33,45]. In chitosan- and alginate-containing systems, ionic interactions and hydrogen-bonded network formation have been shown to improve structural stability and reduce humidity sensitivity. Ref. [41], for example, reported that crosslinked starch–chitosan–alginate adhesive systems maintained measurable adhesion performance even after exposure to high-humidity conditions.

Aromatic biopolymers such as lignin and tannins also represent important platforms for modified bio-based adhesives because of their polyphenolic structure, crosslinking reactivity, and availability from lignocellulosic biomass. Green crosslinkers such as sucrose and citric acid, as well as hydrophobizing agents such as ricinoleic acid, have been explored to improve cohesive strength, water resistance, and compatibility with less polar substrates [38,46]. These systems are particularly relevant because they connect adhesive development with biomass valorisation and the use of agricultural or forestry by-products.

**Nanostructured reinforcement**

Nanostructured reinforcement strategies have also attracted growing attention. Nanocellulose, cellulose nanofibres, nanoclays, graphene derivatives, silica nanoparticles, and biochar-based fillers are increasingly incorporated into adhesive formulations to improve stiffness, barrier behaviour, thermal resistance, and crack resistance while maintaining relatively low density and renewable content [42,47]. In many cases, these nanostructures also influence rheology, wetting behaviour, and stress transfer at the adhesive interface, contributing to improved adhesion performance on paper, biopolymer films, and coated substrates [45,47].

The reinforcing role of cellulose nanofibrils is particularly relevant in this context. Ref. [42] described the use of cellulose nanofibrils as reinforcement in polyvinyl acetate and starch-based adhesive formulations, reporting improved lap-joint strength, increased viscosity, and enhanced moisture resistance at optimized nanofibril contents. This type of result is important because it shows that nanoscale reinforcement can simultaneously modify cohesive strength, interfacial stress transfer, and process-relevant rheology, although excessive filler loading may increase viscosity and complicate industrial application.

Similar strategies have been explored in reinforced starch, soy-protein, and chitosan systems intended for paper-based packaging and biodegradable film applications [33,38]. In reinforced chitosan and alginate systems, nanostructures may also contribute to oxygen-barrier behaviour and active functionality, thereby shifting the role of the adhesive from a simple bonding layer to a multifunctional interfacial material.

**Blends, hybrid bio-based systems, and biomass-derived additives**

Blending strategies constitute another important route for tuning adhesive performance. Combining proteins, polysaccharides, plasticizers, lignin derivatives, tannins, natural rubber latex, cellulose-based modifiers, or synthetic biodegradable polymers allows the simultaneous optimisation of viscosity, flexibility, tack, drying behaviour, and moisture resistance [37,42]. Ref. [41], for example, demonstrated that blending starch, chitosan, and sodium alginate within a crosslinked adhesive network significantly improved sealing performance for biodegradable films compared with unmodified starch-based systems.

The use of lignocellulosic residues is another relevant direction. Ref. [48] investigated lignin isolated from sugarcane bagasse as a bio-based polyol for reactive hot-melt polyurethane adhesives, with silica nanoparticles added as reinforcing agents. The resulting systems showed improvements in bonding strength, thermal stability, viscosity control, and antibacterial activity with increasing lignin content, although the adhesive strength of the bio-based formulations remained below that of the commercial reference. This case is useful because it illustrates both the potential and the current limitations of agro-industrial waste-derived adhesive systems: they can improve renewable content and introduce additional functionality, but they may still require formulation optimisation to achieve the performance of established commercial products.

**Multifunctional adhesive interfaces**

Recent developments increasingly emphasise multifunctional behaviour in addition to simple adhesion. Reinforced bio-based adhesives are now being investigated not only for bonding performance, but also for antimicrobial activity, oxygen-barrier enhancement, antioxidant functionality, moisture regulation, sensing capability, and compatibility with active-packaging architectures. In this context, the boundary between adhesive layer, coating, and functional interfacial material becomes progressively less distinct, particularly in paper-based and biodegradable packaging systems [34,45].

This multifunctional evolution is especially relevant for paper-based packaging, where the same interfacial layer may contribute to adhesion, fibre consolidation, barrier enhancement, surface protection, and active functionality. However, the integration of active or reinforcing additives must be evaluated carefully because non-degradable fillers, toxic crosslinkers, or poorly compatible additives may compromise recycling, composting, or food-contact safety [6,8].

**Industrial and end-of-life constraints**

Despite these advances, modified bio-based adhesive systems still face important industrial challenges associated with processing stability, batch variability, storage resistance, humidity sensitivity, large-scale manufacturing, and long-term durability under demanding converting conditions [32,42]. In addition, the environmental benefit of a bio-based or compostable adhesive depends strongly on the compatibility between adhesive formulation, substrate type, and end-of-life route. Adhesives that are compostable but applied to non-compostable substrates may provide limited environmental benefit, while bio-based formulations containing non-degradable additives or persistent crosslinkers can hinder compostability or recycling [8].

Food-contact compliance also remains a critical issue for these modified systems, particularly regarding reactive modifiers, nanofillers, and antimicrobial additives, as summarised for bio-based/compostable adhesives in Table 6 [6]. Consequently, current research increasingly focuses on balancing renewable content and biodegradability with the processing robustness, thermal resistance, interfacial reliability, and regulatory compatibility required in industrial packaging applications.

#### 2.2.3. Bio-Derived Polymer Systems

In addition to natural macromolecular adhesives and modified bio-based systems, increasing attention is directed toward engineered bio-derived polymer adhesives designed to combine renewable content, industrial processability, and performance levels closer to those of conventional synthetic packaging adhesives. Unlike traditional protein- or polysaccharide-based systems, these materials are generally formulated through polymer-engineering approaches involving bio-derived monomers, renewable polyols, biodegradable thermoplastics, or partially bio-attributed synthetic polymers. Their development is strongly driven by the need to improve compatibility with modern converting operations while reducing dependence on fossil-derived feedstocks and improving end-of-life options in sustainable packaging systems [32,33,34].

**Compostable hot-melt adhesives**

Among these materials, compostable hot-melt adhesives represent one of the most active and industrially relevant development areas. Conventional hot-melt adhesives used in packaging are typically based on EVA, polyolefins, or synthetic rubbers and are valued for their rapid setting, solvent-free processing, and compatibility with high-speed converting lines [28]. However, their fossil-derived origin and limited compostability have stimulated the development of biodegradable and bio-based alternatives based on PLA, PBS, PBAT, PHA, starch blends, and other biodegradable polyester systems. These materials are increasingly investigated for carton sealing, paper packaging, biodegradable laminates, compostable flexible packaging structures, and mono-material or fully compostable multilayer designs in which non-degradable interlayers must be avoided [49,50,51].

Compared with protein- and polysaccharide-based systems, compostable hot-melts generally provide broader processing windows, improved thermal stability, lower moisture sensitivity, and better compatibility with hydrophobic biodegradable substrates. Among biodegradable polyesters, PHAs have attracted attention because of their biodegradability and compostability, although their brittleness and relatively narrow processing window often require formulation optimisation. Ref. [49] developed a biodegradable hot-melt adhesive based on PHA and silanized cellulose nanofibres, showing that surface modification of nanofibres can improve dispersion within the hydrophobic polymer matrix and contribute to improved mechanical and rheological behaviour.

PBAT-based systems also represent a promising route for compostable hot-melt adhesives. Ref. [52] developed PBAT/rosin maleic resin (RMR) hot-melt adhesive system in which the addition of RMR as a tackifier increased shear strength above that of a conventional EVA-based reference (3.9 MPa), reaching approximately 7.3 MPa at 30 wt% RMR content. This example is particularly relevant because it shows that biodegradable polyester adhesives can approach or exceed conventional hot-melt performance when crystallinity, tackifier content, and amorphous-phase formation are properly controlled.

Recent studies show that compostable hot-melt systems can achieve promising adhesion performance while maintaining industrially relevant rheological behaviour and thermal processability. Nevertheless, their practical implementation remains influenced by softening temperature, viscosity control, thermal ageing, crystallisation behaviour, tackifier compatibility, and adhesion to paper or biodegradable polymer substrates. In many cases, the optimisation of tackifiers, plasticizers, biodegradable polyesters, and reinforcing additives is necessary to balance adhesion strength, flexibility, and processing stability [42,50].

From a packaging-system perspective, compostable hot-melt adhesives are particularly relevant when the whole packaging structure is designed for compostability according to standards such as EN 13432. Their contribution is limited if the adhesive is compostable but the substrate, coating, or laminate architecture is not compatible with the same end-of-life route. Therefore, compostable hot-melt adhesives should be evaluated not only as isolated materials, but as components of complete packaging systems, including substrates, coatings, inks, additives, and processing conditions.

**Bio-based polyurethane adhesives**

Bio-based polyurethane adhesives constitute another important category of emerging bio-derived systems. Polyurethane adhesives remain among the most important industrial materials for multilayer flexible packaging because of their excellent adhesion versatility, flexibility, and compatibility with chemically dissimilar substrates. Current sustainability strategies therefore increasingly focus on partially replacing fossil-derived components with renewable polyols obtained from vegetable oils, lignin, castor oil, carbohydrates, or other biomass-derived feedstocks while preserving the processing and mechanical advantages of conventional polyurethane systems [21,22].

These partially bio-attributed systems are particularly attractive because they allow for gradual industrial transition without requiring radical modifications of existing converting infrastructure. Bio-based polyurethane adhesives can be designed for solvent-based, solventless, waterborne, or hot-melt processing routes, depending on the intended application. For flexible food packaging, their main advantage lies in the possibility of combining adhesion to dissimilar substrates, mechanical flexibility, resistance to converting stresses, and reduced fossil-derived content. However, their sustainability profile depends on the proportion of renewable components, the nature of the isocyanate chemistry, residual monomers, migration behaviour, and compatibility with recycling or composting routes.

Recent studies on vegetable-oil-derived and rosin-derived polyurethane adhesives show that bio-based polyols can provide competitive adhesion and thermal resistance, especially when formulation design preserves appropriate crosslink density and interfacial polarity [21,22]. Lignin-derived polyols and agro-industrial residues are also increasingly investigated as renewable components for polyurethane adhesives, although viscosity control, batch variability, colour, odour, and long-term stability remain relevant technological constraints [48].

**Non-isocyanate polyurethanes**

At the same time, increasing regulatory pressure associated with diisocyanates has stimulated growing interest in non-isocyanate polyurethane systems. These materials are generally synthesised through cyclic carbonate–amine reactions and are investigated as safer and potentially more sustainable alternatives to conventional polyurethane chemistry. NIPU adhesives can provide good thermal stability, chemical resistance, and adhesion performance, while avoiding the direct use of isocyanates during synthesis [21,22].

However, their industrial implementation in packaging applications remains limited by relatively slow polymerisation kinetics, long curing times, viscosity management challenges, and performance gaps compared with highly optimised commercial polyurethane systems. Their potential is therefore significant, but their current role is still mainly developmental, particularly for high-speed packaging-converting operations where curing rate, pot life, coating weight, and lamination speed are critical.

**Hybrid and multifunctional bio-derived systems**

Recent developments increasingly explore multifunctional and hybrid bio-derived adhesive systems capable of combining adhesion with additional barrier, antimicrobial, antioxidant, or active-packaging functionalities. In many cases, these systems integrate biodegradable polyesters, nanostructured fillers, reactive biopolymers, lignin-derived components, or bio-derived crosslinkers in order to improve interfacial stability, thermal resistance, and compatibility with recyclable or compostable packaging architectures [33,34]. As a result, bio-derived polymer systems are progressively evolving from simple “green alternatives” toward engineered interfacial materials specifically tailored for advanced sustainable packaging applications.

As with the modified bio-based systems discussed in Section 2.2.2, important challenges remain for bio-derived polymer adhesives regarding large-scale production, food-contact compliance, and economic competitiveness relative to established synthetic systems. Narrow thermal processing windows may limit line speed or increase sensitivity to process fluctuations, whereas excessively low softening temperatures can compromise bond strength under service conditions. Consequently, these systems are currently more suitable for packaging applications with moderate thermal and mechanical demands than for retortable, sterilisation-resistant, or high-temperature environments.

Overall, bio-derived polymer adhesives represent a transitional but strategically important class of materials. Their future development will depend on the ability to combine renewable or bio-attributed chemistry with industrially robust processing, reliable adhesion to both paper-based and bioplastic substrates, controlled migration behaviour, and end-of-life compatibility at the level of the complete packaging system rather than the adhesive layer alone.

### 2.3. Functional and Hybrid Adhesives

Functional and hybrid adhesives represent an emerging class of interfacial materials designed not only to ensure bonding performance, but also to integrate additional functions related to compatibility, circularity, sensing, thermal management, and advanced packaging-system integration. Unlike conventional structural adhesive systems, these materials operate at the interface between adhesion, functionality, and end-of-life engineering, contributing to increasingly complex and multifunctional packaging architectures [33].

As schematically illustrated in Figure 6, the main development pathways of functional and hybrid adhesive systems can be grouped into interfacial compatibility strategies, smart and electronic packaging applications, bio-hybrid formulations, functional labeling systems, and system-level constraints associated with recyclability, food-contact compliance, and future circular packaging requirements.

The following subsections therefore discuss the principal categories of functional and hybrid adhesive systems currently investigated for advanced packaging applications, with particular attention to interfacial performance, multifunctional behaviour, system integration, and emerging sustainability-oriented design approaches.

**Interfacial Compatibility and Tie-Layer Adhesives**

As introduced in Section 2.1, tie-layer and compatibilizing adhesives—typically maleic-anhydride-grafted or otherwise functionalized polyolefins—are essential for promoting adhesion between chemically dissimilar substrates such as polyethylene, polypropylene, polyamide, EVOH, and PET in co-extruded multilayer structures. Within the broader context of functional and hybrid adhesive systems, these materials are increasingly required not only to ensure barrier continuity and structural integrity during converting and service, but also to support compatibilization strategies that balance adhesion performance with recyclability and reduced material complexity, particularly in emerging mono-material packaging concepts [53,54].

Recent research also explores selectively debondable and stimuli-responsive adhesive interfaces capable of maintaining interfacial stability during service while enabling controlled delamination during recycling or dismantling operations. These systems may respond to thermal, chemical, ultraviolet, or electrically induced stimuli, allowing selective weakening of either cohesive adhesive integrity or adhesive–substrate interactions [55]. Such approaches are increasingly considered promising strategies for design-for-disassembly packaging and improved recovery of multilayer polymer streams [54,56].

The progressive transition toward recyclable and circular packaging systems has therefore stimulated increasing interest in adhesive interfaces capable of combining strong interfacial bonding during service with improved compatibility with downstream recycling operations. Although fully debondable and triggerable interfaces remain largely developmental and are discussed further in Section 5, current multilayer packaging design already increasingly considers the role of adhesive layers as critical determinants of material separation efficiency, polymer purity, and end-of-life performance.

**Functional Adhesives for Smart and Electronic Packaging**

Recent developments increasingly focus on functional adhesive systems capable of providing additional thermal, electrical, sensing, or signal-transmission functions within packaging architectures. Unlike conventional structural adhesives, these systems are designed not only to ensure bonding performance, but also to support advanced functionalities associated with smart packaging, flexible electronics, RFID integration, printed sensors, and thermal-management applications.

Thermally conductive adhesive systems have attracted growing attention in this context. These materials are commonly based on epoxy, polyurethane, silicone, or hybrid polymer matrices filled with ceramic, metallic, or carbonaceous particles capable of improving heat dissipation while maintaining adequate mechanical integrity. As discussed by [57], thermally conductive fillers including boron nitride, aluminum nitride, alumina, silicon carbide, silver, copper, graphene, and carbon nanotubes can significantly enhance thermal transport within adhesive matrices, thereby improving thermal management and structural stability in miniaturized electronic systems.

Although these technologies are not yet dominant in conventional food packaging, their design principles are increasingly relevant for advanced smart-packaging architectures integrating sensing and monitoring functionalities. Adhesive layers may therefore contribute simultaneously to bonding, thermal regulation, electrical conductivity, and signal transmission, particularly in packaging systems incorporating RFID tags, temperature indicators, humidity sensors, printed circuits, or flexible electronic devices [58].

Recent work on sustainable microelectronic packaging further extends this perspective by showing that bio-derived adhesive concepts, including plant-based epoxy matrices and bacterial bioadhesives, may combine interfacial adhesion with thermal, electrical, or coupling-agent functions in advanced electronic assemblies [59].

Conductive and thermally active adhesive systems also illustrate the broader evolution of packaging interfaces from passive joining layers toward multifunctional interfacial materials capable of integrating structural and functional roles [57,59]. In many cases, these systems combine conductive fillers with hybrid polymer matrices in order to balance adhesion performance, flexibility, processability, and functional response. However, increasing formulation complexity may also influence viscosity control, coating uniformity, migration behaviour, recyclability, and food-contact compatibility, thereby requiring careful optimisation for packaging applications [60].

Despite their current use being largely restricted to specialized or high-value packaging sectors, smart and electronically functional adhesives are expected to become increasingly important as packaging systems progressively integrate digital technologies, traceability functions, active monitoring, and intelligent supply-chain management strategies [58,59].

**Bio-Hybrid and Renewable Functional Adhesives**

Another important development pathway concerns hybrid adhesive systems combining synthetic polymer backbones with renewable or bio-derived components. These materials aim to retain the processing stability, adhesion reliability, and mechanical robustness of conventional synthetic adhesives while partially reducing fossil-derived content and improving sustainability-related performance.

As discussed in Section 2.2.2 and Section 2.2.3, these renewable components—including starch derivatives, lignin, tannins, vegetable oils, and biodegradable polyesters—can be incorporated within synthetic or semi-synthetic matrices to obtain bio-attributed polyurethanes, starch-modified hot-melt systems, or waterborne polyurethane dispersions for recyclable or compostable packaging [16,19,61].

Hybridisation strategies are particularly relevant because purely bio-based adhesive systems often exhibit limitations associated with humidity sensitivity, reduced thermal stability, or insufficient adhesion to hydrophobic substrates. The incorporation of synthetic segments, amphiphilic modifiers, or biodegradable polyester phases can therefore improve processing robustness and interfacial reliability while maintaining partially renewable character [16,61]. Similarly, lignin, tannins, and agricultural by-products are increasingly investigated not only as renewable feedstocks, but also as functional components capable of modifying cohesive strength, hydrophobicity, antioxidant behaviour, or thermal response [19,59].

Recent research also explores hybrid systems integrating biodegradable polymers with non-isocyanate polyurethanes, reactive bio-based crosslinkers, or multifunctional nanostructures in order to improve compatibility with modern packaging-converting operations [59]. These approaches increasingly blur the distinction between structural adhesive layers, coatings, compatibilizers, and multifunctional interfacial materials, particularly in sustainable multilayer and paper-based packaging systems [8].

Nevertheless, the introduction of renewable fillers or bio-derived modifiers may increase formulation variability, influence curing behaviour, alter rheological stability, or affect migration characteristics. Consequently, bio-hybrid systems must be evaluated not only according to renewable content, but also with respect to processing stability, substrate compatibility, food-contact compliance, and end-of-life behaviour within complete packaging architectures [60,62,63]. These systems are therefore often viewed as transitional solutions capable of improving sustainability performance while preserving industrial processability and interfacial reliability.

**Functional Labeling and Wash-Off Adhesive Systems**

Labeling adhesives represent a particularly important class of functional packaging adhesives, as they must simultaneously ensure reliable adhesion under refrigerated, humid, or mechanically demanding service conditions and controlled removability during industrial washing processes for recycling or reuse. Most systems are water-based formulations designed to balance adhesion strength, moisture resistance, removability, and processability on glass and PET containers. Casein-based and modified natural-polymer systems remain particularly relevant in this context because they provide good adhesion to polar substrates together with controlled debonding behaviour during alkaline washing operations. Ref. [35], for example, developed a casein-based adhesive modified with glycerin ester of gum rosin capable of adhering effectively to both glass and PET while maintaining removability under industrial washing conditions at 70–80 °C. The formulation also exhibited resistance to condensed water and refrigerated storage conditions while still allowing efficient label detachment during recycling and reuse operations.

These systems are especially important from a circular-packaging perspective because adhesive residues may strongly affect the quality of recycled polymer streams or interfere with bottle reuse processes. Recent studies on PET circularity and recycling efficiency increasingly identify labels, adhesive residues, and interfacial contaminants among the major factors influencing recyclate purity and recovery efficiency. Recent developments also explore removable pressure-sensitive systems, water-dispersible adhesives, alkali-removable acrylic formulations, and selectively washable label technologies compatible with automated sorting and recycling systems [64,65].

**System-Level Constraints and Future Transition Pathways**

Despite the rapid development of functional and hybrid adhesive systems, several important challenges remain regarding industrial implementation, regulatory compliance, and system-level integration within packaging architectures. Increasing formulation complexity may influence rheological stability, coating uniformity, curing behaviour, converting compatibility, long-term durability, and substrate interactions, particularly in high-speed industrial packaging operations.

Food-contact compliance, summarised for functional/hybrid systems in Table 6, represents one of the most critical constraints for advanced adhesive systems incorporating conductive fillers, reactive modifiers, or multifunctional additives [60,62,63]. Consequently, the development of multifunctional adhesive systems increasingly requires simultaneous optimisation of adhesion performance, functional behaviour, migration control, and processability.

Another important challenge concerns compatibility with recycling and circular packaging strategies. Adhesive interfaces that ensure excellent bonding during service may hinder material separation and reduce the quality of recovered polymer streams at end of life [54,65]. For this reason, current packaging design increasingly considers adhesive systems not as isolated materials, but as integral components of complete packaging architectures involving substrates, coatings, barrier layers, inks, and recycling pathways [19].

These considerations are progressively driving the development of advanced interfacial concepts focused on selective adhesion, controlled delamination, recyclable multilayer architectures, and circularity-oriented packaging engineering [54,56]. Although many of these approaches remain at an early technological stage, they are expected to play an increasingly important role in future packaging systems designed according to recyclability, material recovery, and design-for-disassembly principles.

Overall, functional and hybrid adhesives increasingly transform packaging interfaces from passive joining layers into multifunctional interfacial systems capable of supporting recyclability, smart functionalities, system integration, and future circular packaging strategies.

Table 1 summarises the main performance trade-offs among the adhesive families discussed in Section 2. Table 1 provides a family-level comparison of adhesive chemistries in terms of application area, processing compatibility, performance-related strengths and limitations, circularity profile, and regulatory maturity, while Table 6 details food-contact and migration considerations for the same families. The comparison is qualitative and intended as a design-oriented synthesis, since performance depends strongly on formulation, substrate type, processing conditions, and test method.

## 3. Sealant Materials for Packaging Applications

Sealant materials represent a critical functional layer in modern packaging systems since they determine not only package closure but also the continuity of barrier properties, mechanical integrity, and product protection during storage, distribution, and use. Unlike adhesives, whose primary role is to join dissimilar substrates within multilayer architectures, sealants are directly involved in the formation of hermetic seams and must therefore operate within well-defined thermal, mechanical, and processing windows. Their performance is governed by the combined effects of seal initiation temperature, seal strength, hot-tack behaviour, dwell time, pressure, substrate compatibility, and resistance to thermal or environmental stresses.

In advanced packaging, sealants can no longer be treated as simple inner layers selected only for heat-sealability. They must be evaluated as system-level materials that interact with barrier layers, structural films, coatings, tie layers, and end-of-life requirements. Conventional thermoplastic sealants such as LDPE, PP, and ionomers still provide the industrial benchmark for sealing reliability and process robustness, while multilayer barrier sealants, biodegradable systems, and emerging non-thermal or active sealing technologies are increasingly investigated to address shelf-life extension, circularity, compostability, and functional packaging needs. Accordingly, this section analyses the main classes of sealant materials by considering their composition, sealing mechanism, quantitative performance metrics, processing compatibility, and sustainability-related constraints.

The structure of Section 3 is summarised in Figure 7, which illustrates the technological progression from conventional thermoplastic sealants to advanced, sustainable, and non-thermal sealing solutions, together with the main cross-cutting performance and system-level considerations governing modern packaging applications.

### 3.1. Conventional Thermoplastic Sealants

Conventional thermoplastic sealants constitute the industrial reference for heat-sealed packaging, providing the baseline against which advanced and bio-based sealing solutions must be evaluated. Materials such as low-density polyethylene (LDPE), polypropylene (PP), and ionomers dominate flexible and semi-rigid packaging due to their broad sealing windows, robustness under high-speed processing, and compatibility with a wide range of substrates and converting technologies.

LDPE-based sealants are the most widely used owing to their low melting temperature (typically 100–160 °C) and wide seal initiation range, which allow reliable sealing under variable pressure, dwell time, and temperature. These characteristics make LDPE particularly tolerant to process fluctuations and surface contamination, enabling consistent seal strength in industrial environments. As a result, LDPE often serves as the inner sealing layer in multilayer films for food and pharmaceutical packaging [7,66].

Polypropylene sealants, while requiring higher sealing temperatures than LDPE (110–170 °C), offer improved stiffness, chemical resistance, and thermal stability. These properties make PP-based sealants suitable for applications involving hot filling, microwave heating, or retort processing. However, their narrower sealing window and higher seal initiation temperature demand tighter process control, particularly in high-speed lines [67,68].

Ionomers, typically based on ethylene copolymers neutralized with metal ions, represent a high-performance subset of thermoplastic sealants. They provide excellent sealing at lower temperatures (90–150 °C) and maintain superior seal integrity even under contamination. These properties make ionomers particularly valuable in packaging for fatty foods, medical devices, and applications requiring high seal integrity under mechanical stress [7,69,70]. Ionomers occupy a niche characterized by high functional performance but higher material cost.

Figure 8 summarises representative industrial applications and typical use scenarios of LDPE-, PP-, and ionomer-based sealants, highlighting how differences in sealing behaviour, thermal resistance, and process robustness influence packaging selection.

From a performance standpoint, conventional thermoplastic sealants are typically evaluated using quantitative metrics such as seal strength, seal initiation temperature (SIT), and hot-tack behaviour. These parameters define not only the ultimate mechanical integrity of the seal, but also its formation during dynamic packaging operations. Thermoplastics generally exhibit favourable hot-tack performance, enabling seals to withstand tensile stresses immediately after formation, a critical requirement in vertical form–fill–seal and high-speed horizontal packaging lines [66,71].

Despite their proven reliability, conventional thermoplastic sealants present limitations in the context of sustainability and circular design. Their petrochemical origin and limited compatibility with composting streams constrain their use in fully bio-based packaging concepts. In addition, when incorporated into complex multilayer structures, these sealants can hinder recyclability by introducing material heterogeneity that complicates separation and reprocessing. Modifications with additives or blend formulations (e.g., with EVA) further extend their sealing capabilities and adaptability across packaging formats [7,72]. While sustainability concerns are prompting a shift toward biodegradable alternatives, LDPE, PP, and ionomers continue to serve as standard references for heat sealing performance and industrial scalability. Consequently, in the context of this review, conventional thermoplastic sealants are presented as performance benchmarks rather than end-point solutions. Their well-established processing windows, mechanical reliability, and industrial maturity provide a necessary reference for assessing the feasibility, advantages, and limitations of advanced barrier sealants, biodegradable systems, and non-thermal sealing concepts discussed in subsequent sections.

### 3.2. Multilayer Barrier Sealants

Multilayer barrier sealants are used in packaging applications where conventional single-material sealing layers cannot simultaneously provide hermetic closure, high barrier performance, mechanical stability, aroma retention, and extended shelf life. In these systems, the sealant is no longer an isolated heat-sealable material, but a functional interfacial layer operating within heterogeneous multilayer architectures designed to combine sealing performance with oxygen and moisture protection [7,73].

Unlike conventional thermoplastic sealants, which mainly provide seal formation and hot-tack behaviour, multilayer barrier sealants must preserve barrier continuity across the sealing area while maintaining adhesion between chemically and thermally dissimilar layers. Consequently, the performance of these systems depends not only on seal strength, but also on interfacial compatibility, thermal matching, resistance to delamination, and stability under demanding service conditions such as sterilisation, vacuum packaging, retort processing, or modified-atmosphere packaging (MAP) [66,74].

In multilayer barrier systems, the term “barrier sealant” does not generally indicate that the barrier polymer itself acts as the sealing layer. Rather, it refers to multilayer sealing architectures in which a heat-sealable inner layer is integrated with high-barrier components and intermediate adhesion-promoting layers. The actual sealing layer is typically based on LDPE, LLDPE, PP, ionomers, or compatibilised polyolefin systems, selected for their ability to provide hermetic closure, hot-tack performance, and process robustness. Barrier materials such as EVOH, PVDC, inorganic coatings, or nanocomposite-enhanced layers instead provide protection against oxygen or moisture permeation, while tie layers or functionalised polymers ensure adhesion between otherwise incompatible materials.

To achieve these combined functions, multilayer structures typically distribute distinct roles among different layers. Structural outer layers provide stiffness, printability, and dimensional stability; barrier layers limit gas or vapour permeation; tie layers maintain interfacial adhesion; and the sealant layer ensures cohesive sealing without compromising barrier continuity. Therefore, the effectiveness of multilayer barrier sealants depends strongly on the integrity of the sealing interface and on the compatibility between the sealant layer and adjacent materials. A representative multilayer barrier sealant architecture and the role of the sealant layer within the multilayer structure are illustrated in Figure 9.

Within this framework, EVOH-based multilayer systems are among the most widely adopted because of the excellent oxygen barrier provided by EVOH under dry conditions. EVOH is commonly incorporated as a thin core layer within laminated or co-extruded structures positioned between polyolefins or polyesters. These systems are extensively used in vacuum-packed meats, dairy products, thermoformed trays, and sensitive dry foods requiring prolonged shelf life and aroma retention. However, the moisture sensitivity of EVOH imposes strict requirements on the surrounding layers and on the sealing interface, since local discontinuities or interfacial defects may compromise barrier performance under humid conditions. Consequently, adjacent layers such as PET, PA, or polyolefins must simultaneously provide mechanical support and protect the EVOH barrier from moisture exposure [73,75].

PVDC-containing systems provide combined resistance to oxygen and water vapour, particularly under humid conditions, and have historically been used in high-value food and pharmaceutical packaging where transparency and long shelf life are critical. Nevertheless, increasing environmental and recyclability concerns related to chlorine-containing structures and end-of-life management have progressively limited their adoption [76].

More recently, nanocomposite-enhanced sealants have emerged as an alternative strategy to improve barrier and mechanical performance while maintaining flexibility and sealability. By incorporating platelet-like fillers such as montmorillonite nanoclays into polyolefin matrices, these systems increase the tortuosity of permeation pathways for gases and improve thermomechanical resistance through filler–polymer interfacial interactions. Significant increases in Young’s modulus, typically ranging from approximately 80% to 160% at nanofiller contents between 2 and 5 wt%, have been reported while preserving compatibility with conventional heat-sealing processes [77,78,79,80]. In addition to enhanced barrier performance, these systems may also maintain good optical transparency and reduced material thickness compared with conventional multilayer solutions.

The complexity of multilayer barrier systems introduces additional challenges at the sealing interface. During heat sealing, the sealant layer must accommodate differences in melting temperature, modulus, thermal expansion, and surface polarity between adjacent layers. Inadequate compatibility may lead to interfacial failure, local barrier discontinuity, stress concentration, or delamination phenomena that compromise package integrity and shelf life. Consequently, the sealing interface becomes a critical functional region rather than a simple joining area. The principal mechanisms governing barrier continuity and seal integrity in multilayer systems are schematically summarised in Figure 10 [74].

Despite their functional advantages, multilayer barrier sealants remain associated with significant recyclability and circularity challenges because the combination of chemically incompatible materials complicates mechanical recycling and material separation [54,81]. Nevertheless, these systems remain essential for high-performance packaging applications requiring extended shelf life and advanced barrier protection, including retort pouches, thermoformed trays, vacuum packaging, and MAP structures [82,83]. Current mitigation strategies include down-gauging of barrier layers, development of polyolefin-compatible barrier systems, mono-material multilayer concepts, and design-for-recycling approaches aimed at maintaining dominant material streams while preserving sealing and barrier performance [54,81].

Within the context of this review, multilayer barrier sealants therefore represent not only a class of high-performance sealing systems, but also a key example of the growing tension between packaging functionality and circularity requirements. Figure 11 comparatively summarises the main multilayer barrier sealant technologies, their functional roles, and the associated trade-offs between barrier performance, processability, seal integrity, and recyclability.

### 3.3. Biodegradable and Bio-Based Sealants

Biodegradable and bio-based sealants have emerged as a strategic response to the growing demand for sustainable packaging systems compatible with compostability, renewable feedstocks, and reduced environmental impact. Unlike conventional thermoplastic sealants, which are primarily optimised for processing robustness and long-term durability, biodegradable sealants aim to combine heat-sealability with renewable origin and compatibility with organic recycling or composting routes [7,84].

These materials include a broad range of polymer families characterised by markedly different sealing behaviours, thermal stability, moisture sensitivity, and mechanical performance. The most industrially relevant systems are currently based on aliphatic polyesters such as polylactic acid (PLA), polybutylene succinate (PBS), and polybutylene adipate terephthalate (PBAT), which are frequently blended or compatibilised to improve flexibility, hot-tack behaviour, and processability [7,85]. Alongside these materials, polysaccharide- and protein-based systems derived from starch, cellulose, chitosan, gelatin, whey, or soy proteins have attracted increasing attention for edible films, paper coatings, and compostable packaging applications [86,87,88].

Despite their sustainability advantages, biodegradable sealants generally exhibit narrower processing windows, greater sensitivity to moisture and thermal ageing, and lower tolerance to industrial process fluctuations compared with conventional polyolefin-based sealants. Moisture uptake, creep under load, and hydrolytic degradation may progressively compromise seal integrity and long-term package performance, particularly under demanding environmental conditions [32,89]. Consequently, significant research efforts have focused on polymer blending, compatibilisation, nanostructuring, multilayer integration, surface functionalisation, and self-healing approaches aimed at improving seal integrity and industrial applicability [90,91,92].

The principal families of biodegradable and bio-based sealants discussed in this section are schematically summarised in Table 2.

#### 3.3.1. Aliphatic Polyester-Based Sealants

Aliphatic polyesters currently represent the most industrially relevant class of biodegradable sealants for compostable packaging applications. Their importance derives from the combination of thermoplastic processability, heat-sealability, transparency, and compatibility with industrial composting routes, making them suitable for flexible films, multilayer laminates, pouches, sachets, and thermoformable packaging systems [7,84].

Among these materials, PLA, PBS, and PBAT are the most extensively investigated. Although all three materials are used in biodegradable sealing systems, they exhibit markedly different thermal behaviour, flexibility, sealing response, and resistance to process fluctuations. Consequently, industrial formulations frequently rely on blending and compatibilisation strategies aimed at balancing seal initiation temperature, hot-tack behaviour, ductility, and mechanical stability.

PLA is among the most investigated bio-sealants, attracting considerable attention due to its industrial compostability, transparency, and renewable origin. PLA homopolymer exhibits a glass transition temperature of 55–60 °C and a melting temperature of approximately 170 °C, with crystalline forms reaching melting points around 186 °C. PLA-based compositions are typically applied at temperatures ranging from 110 °C to 150 °C. However, performance limitations arise from the material’s relatively high glass transition temperature, narrow processing window, and brittle nature. PLA-based polymers are also susceptible to rapid hydrolytic and environmental degradation, particularly when residual monomers are present or when low initial molecular weight compromises stability. These characteristics impose strict control over temperature, pressure, and dwell time during application, increasing sensitivity to process variability and limiting robustness in high-speed packaging lines [84,89].

PBS and PBAT offer improved flexibility and lower sealing temperatures compared to PLA, making them attractive as sealant layers in compostable multilayer films. PBS is a rather soft semicrystalline material with a glass transition temperature around −32 °C and melting temperature around 115 °C. PBS-based sealants typically exhibit broader sealing windows and better hot-tack behaviour, while PBAT contributes ductility and resistance to brittle failure. Blends of PLA with PBS or PBAT are frequently employed to tailor seal initiation temperature and mechanical compliance, improving industrial processability. PLA/PBAT blends have demonstrated seal strengths at interfacial temperatures between 76 °C and 105 °C, representing a significantly broader processing window than pure PLA. The addition of PBAT to PLA results in approximately 20 °C decrease in seal initiation temperature, attributed to changes in the crystalline structure. Hot tack initiation occurs at approximately 75 °C for both PLA and PLA/PBAT blends [7]. Complementary evidence has been provided by blown film studies on PBAT/PLA blends compatibilised with a chain extender, which demonstrated hot-tack strength comparable to conventional PE films at 85 °C, with morphological analysis confirming that the reactive compatibilisation improves interfacial adhesion between the two phases [85]. PBAT, in particular, is widely used as a soft component in multilayer compostable films.

The general characteristics, advantages, and limitations of the principal aliphatic polyester-based sealants used in biodegradable packaging systems are comparatively summarised in Table 3.

#### 3.3.2. Polysaccharide- and Protein-Based Sealants

Beyond aliphatic polyesters, bio-based packaging solutions have gained attention across multiple application areas.

Protein-based edible films derived from whey, casein, gelatin, and soy proteins have been extensively developed for food packaging applications, offering biodegradability, barrier properties against oxygen and oils, and the ability to incorporate antimicrobial and antioxidant agents [86,93]. Polysaccharide-based edible packaging materials, including chitosan, starch, and cellulose, dominate the sustainable packaging market due to their excellent film-forming abilities, renewability, and low processing costs [87,88]. For non-edible applications, polysaccharide-based adhesives using chitosan, carboxymethyl cellulose, and starch have been developed for paper and wood substrates, with polyelectrolyte complex formulations showing improved water resistance and comparable strength to synthetic adhesives [39].

Gelatin-, starch-, and chitosan-derived systems can form heat-sealable layers when appropriately plasticised or blended, but their high sensitivity to moisture and limited mechanical durability restrict their use to controlled environments. Starch-based sealants, often combined with polyesters or natural waxes, present good biodegradability and cost-efficiency. Thermoplastic starch blends can form heat-sealable coatings on paper or biofilms, though they require careful control of moisture content and adhesion modifiers [32,94,95]. Such systems are generally unsuitable for high-humidity or long-shelf-life applications without additional coatings or barrier layers.

As already noted in Section 3.3, these moisture- and creep-related limitations underscore the importance of integrating biodegradable sealants within system-level designs encompassing substrate selection, coating strategies, package geometry, and formulation optimization. Several strategies have been investigated to overcome the intrinsic limitations of biodegradable sealants. Plasticisation (1) is commonly used to improve flexibility and reduce seal initiation temperature, although excessive plasticiser content may compromise mechanical stability and moisture resistance. Polymer blending (2), particularly with aliphatic polyesters such as PBS or PBAT, is widely adopted to enhance ductility, hot-tack behaviour, and processability. Coating and multilayer approaches (3) enable the combination of biodegradable sealants with protective barrier layers or fibrous substrates in order to improve moisture resistance and structural stability. Additional improvements may also be achieved through chemical modification (4) and nano-/micro-scale reinforcement strategies (5), aimed at enhancing interfacial adhesion, barrier continuity, and mechanical durability. The principal approaches currently adopted to improve biodegradable sealant performance are schematically summarised in Figure 12.

These materials are essential for the development of certified compostable packaging formats under EN 13432 [9] or ASTM D6400 [10] standards and are increasingly found in films labelled as “home-compostable” or suitable for organic waste streams.

#### 3.3.3. Advanced Functional and Nanostructured Bio-Sealants

Recent developments have further expanded the landscape of biodegradable sealing materials through both empirical studies and conceptual innovations.

Current research efforts are primarily focused on three main directions: nanostructured and active bio-sealants, coating-based and recyclable sealing systems, and multifunctional self-healing materials designed to improve durability, barrier stability, and processability under industrial packaging conditions.

**Nanostructured and Active Systems**

Recent research increasingly explores nanostructured sealants designed not only to improve heat-sealing performance, but also to integrate enhanced barrier properties, mechanical reinforcement, antimicrobial activity, and multifunctional packaging capabilities within sustainable systems. In this context, nanofillers such as cellulose nanocrystals, nanoclays, chitosan nanostructures, and inorganic nanoparticles are increasingly incorporated into biodegradable matrices to improve sealability, thermal stability, moisture resistance, and interfacial performance [96,97,98].

Early studies demonstrated that nanoparticles can significantly improve the sealing behaviour of starch-based biodegradable films. Ref. [96] showed that the incorporation of nanoclay and nanosilica into starch films enhanced mechanical strength, barrier performance, thermal stability, and heat sealability compared with unfilled systems. The study highlighted that nanostructured fillers can improve seal strength while maintaining the biodegradability of starch-based packaging materials.

Subsequent developments increasingly focused on nanocellulose-reinforced multilayer systems and multifunctional coatings. Ref. [99] developed cassia-gum edible films reinforced with carboxylated cellulose nanocrystal whiskers (C-CNCW), obtaining substantial improvements in mechanical resistance, oil barrier properties, and heat-seal strength. The incorporation of nanocellulose increased seal strength from 1295 to over 2200 N/m while simultaneously improving barrier performance, demonstrating the potential of nanocellulose-reinforced biofilms for edible and oil-packaging applications.

Similarly, Ref. [100] developed multilayer heat-sealable paper-based packaging systems using chitosan, hydrophobically modified cellulose nanofibrils, and zein coatings. Their multilayer architecture combined oxygen, moisture, and grease barrier enhancement with binder-free heat-sealing capability and antibacterial activity, illustrating how nanostructured coatings can simultaneously address sealability, barrier continuity, and food preservation requirements in biodegradable paper-based packaging.

More advanced multifunctional nanocomposite systems were investigated by [90], who developed PVA/cellulose nanocrystal-based heat-sealable nanocomposites functionalized with titanium dioxide nanoparticles and apple peel extract. The resulting materials combined thermoplastic processability and adjustable seal strength with UV-barrier, antioxidant, and antimicrobial functionalities. Importantly, the sealing behaviour could be tuned to generate either peelable or lock-seal configurations depending on sealing temperature, demonstrating the growing transition from passive biodegradable sealants toward multifunctional active packaging systems.

Beyond biodegradable nanostructured systems, similar nanocomposite strategies have also been explored in polyolefin-based sealants to control peelability and sealing behaviour through interfacial engineering. Refs. [77,101] demonstrated that polyethylene/ethylene-vinyl-acetate nanocomposites reinforced with montmorillonite nanoclays can generate hermetic but peelable heat seals over unusually broad sealing temperature windows. Their studies showed that controlled nanofiller dispersion and weak filler/polymer interfacial interactions promote cohesive peel mechanisms while preserving mechanical robustness and seal integrity.

Further advances were reported by [102], who demonstrated that the dispersion state and distribution of nanoclays strongly influence the peel performance of clay/polyethylene nanocomposite sealants. Compatibilized nanocomposite structures achieved ultra-wide peelable heat-sealing temperature windows exceeding 100 °C, confirming that nanostructural control can directly govern sealing behaviour and peel mechanics in advanced packaging sealants.

**Coating-Based and Recyclable Systems**

Recent studies increasingly focus on the development of heat-sealable systems capable of combining sealing performance with recyclability or compostability, thereby reducing the dependence on conventional multilayer architectures based on incompatible polymers.

In this context, Ref. [82] investigated the sealing behaviour of mono-polyolefin and paper-based laminates under industrially relevant sealing conditions, demonstrating how sealing temperature, pressure, moisture content, and jaw configuration strongly influence seam integrity in recyclable structures. The study showed that mono-polyolefin laminates can achieve hot-tack and cold-tack performances comparable to conventional PET/Al/PE systems, while paper-based coated structures remain more sensitive to delamination and moisture effects because of the limited cohesion of thin sealing coatings. These results highlighted the feasibility of replacing conventional heterogeneous multilayers with more recyclable sealing architectures, although careful optimization of sealing conditions remains necessary for industrial implementation.

Alongside recyclable mono-material approaches, increasing attention has been directed toward fully biodegradable sealing systems based on starch and protein matrices. Ref. [103] investigated edible films based on tapioca starch blended with whey protein concentrate or gelatin, demonstrating that protein incorporation significantly affects tensile behaviour, flexibility, and thermosealing performance. Their results confirmed that multi-component starch–protein systems can generate mechanically stable and heat-sealable biodegradable films suitable for food packaging applications requiring controlled sealing performance.

Ref. [104] further expanded this approach through the development of biodegradable heat-sealable films based on tapioca and potato starch blends plasticised with soy lecithin and glycerol. The films exhibited rapid solubility, complete biodegradation in soil within eight days, and adequate sealing behaviour for edible sachet applications, reinforcing the potential of starch-based systems as sustainable alternatives to synthetic flexible packaging.

A parallel strategy focused on simplifying packaging architecture itself was proposed by [105], who developed PET-based heat-sealable films without conventional foreign sealing layers. Their approach relied on a thin water-based organic–inorganic hybrid sol–gel coating composed of silanes and aluminium oxide nanoparticles, enabling direct heat welding of PET films while simultaneously improving oxygen and water-vapour barrier properties. Unlike traditional multilayer systems requiring incompatible polyolefin sealing layers, the proposed architecture maintained the mono-material character of PET packaging, thereby improving recyclability while preserving transparency, mechanical integrity, and industrial heat-sealing compatibility.

More recently, Ref. [106] focused on improving the industrial applicability of compostable paper-based packaging through optimized heat-sealable starch coatings. Using sodium starch octenyl succinate and maltodextrin plasticised with sorbitol and glycerol, the authors achieved significantly reduced seal initiation temperatures while maintaining full home compostability. The study demonstrated that careful optimization of coating composition and plasticizer content enables paper-based flexible packaging systems to achieve reliable sealing performance without relying on conventional plastic coatings, reinforcing the feasibility of fully compostable paper-based packaging architectures.

**Self-Healing and Multifunctional Systems**

Recent research is increasingly extending biodegradable sealant concepts beyond conventional heat-sealing performance toward adaptive and multifunctional packaging systems capable of preserving barrier integrity, extending service life, and improving durability under mechanical or environmental stress conditions.

In this context, Refs. [91,92] reviewed the emerging field of self-healing packaging films and coatings for food applications, analysing both intrinsic and extrinsic healing mechanisms in biopolymeric materials. Intrinsic systems rely on reversible covalent bonds and dynamic non-covalent interactions, including hydrogen bonding, metal coordination, and host–guest interactions, whereas extrinsic systems employ encapsulated healing agents dispersed in microcapsules or vascular-like networks. The authors highlighted several advanced systems capable of restoring mechanical and barrier functionality after damage, including PLA/tetraethyl citrate films showing complete healing within 120 s and cellulose nanocrystal-reinforced nanocomposites exhibiting healing efficiencies approaching 99% through dynamic hydrogen-bonding and metal–ligand interactions [91,92,107]. Layer-by-layer chitosan/carboxymethyl cellulose multilayer coatings were also reported to recover up to 97% of tensile strength and 95% of oxygen-barrier performance within minutes after moisture exposure. These studies indicate that self-healing behaviour may represent an important future strategy for maintaining seal integrity and barrier continuity in sustainable packaging systems.

Parallel developments have also focused on multifunctional starch-based composite systems designed to improve the limitations of single biopolymers through blending and interfacial optimization. Ref. [108] demonstrated that blends involving starch, PLA, PBAT, PVOH, and proteins can significantly improve the mechanical performance, flexibility, and degradability of biodegradable packaging materials compared with native starch matrices alone.

More recently, Ref. [109] investigated biodegradable films based on heat-moisture-treated potato starch blended with ultrasonicated casein at different starch/protein ratios. The optimized multi-component systems exhibited improved inter-polymer interactions, balanced mechanical resistance, reduced water-vapour permeability, and adequate heat-sealing behaviour while maintaining complete biodegradability under soil conditions. The study further demonstrated how starch–protein hybrid systems can overcome the poor moisture resistance and limited structural durability typically associated with single-component biodegradable films, reinforcing the growing importance of multifunctional bio-based formulations for advanced food-packaging applications.

#### 3.3.4. End-of-Life

End-of-life considerations are central to the development of biodegradable sealants. To provide genuine environmental benefits, sealant layers must remain compatible with the compostability or recyclability of the overall packaging structure rather than being merely bio-derived. Sealants containing non-degradable additives, incompatible blends, persistent inorganic components, or complex multilayer architectures may compromise certification under industrial composting standards and complicate waste-management pathways [7,32,106].

The compatibility between biodegradable sealants, packaging architecture, and the intended disposal route is schematically summarized in Figure 13a, which illustrates the principal end-of-life pathways associated with compostable packaging systems. Figure 13b complements this overview by highlighting the main system-level requirements necessary to ensure effective circularity and compostability, including material compatibility, formulation design, multilayer simplification, and compliance with certification standards.

In addition to intrinsic material biodegradability, the end-of-life performance of biodegradable sealants strongly depends on the compatibility of the entire packaging architecture with the intended disposal route. Industrial composting and home-composting environments differ substantially in temperature, humidity, residence time, aeration, and microbial activity, resulting in major differences in degradation kinetics and certification requirements [110]. Consequently, materials that degrade effectively under controlled industrial composting conditions may not necessarily exhibit equivalent behaviour under home-composting or unmanaged environmental conditions. This issue is particularly relevant for multilayer packaging systems and compatibilised blends, where non-biodegradable additives, barrier coatings, nanofillers, or incompatible sealing layers may hinder complete degradation and compromise certification under standards such as EN 13432 or ASTM D6400 [9,10,32,106].

As anticipated in Section 1, the following discussion further substantiates the distinction between bio-based origin, biodegradability, compostability, and recyclability.

Moreover, and consistently with the distinction introduced in Section 1, certification under industrial composting standards does not guarantee effective degradation under soil, marine, freshwater, or unmanaged environmental conditions [111]. Improper disposal or incompatibility with existing recycling infrastructures may also introduce contamination into conventional recycling streams, while incomplete degradation of compatibilised systems or coated multilayer structures may generate persistent fragments or secondary microplastic-like residues [4,7,97,112].

Figure 13c summarizes the main technological barriers, formulation limitations, environmental sensitivities, and infrastructural challenges that may compromise the effective circularity and end-of-life performance of biodegradable sealing systems.

At the same time, strategies aimed at improving moisture resistance, barrier durability, thermal stability, or service-life performance may also slow degradation kinetics, creating a fundamental trade-off between in-use functionality and end-of-life degradability [89]. Strong interfacial adhesion and complex multilayer architectures, although beneficial during service life, may further hinder material separation, disassembly, and effective composting or recycling operations. The environmental effectiveness of biodegradable sealants therefore depends not only on intrinsic material properties, but also on adequate collection systems, sorting infrastructures, industrial composting facilities, and realistic disposal practices [7,82,105].

As a result, current research increasingly emphasizes system-level approaches integrating material selection, sealant compatibility, multilayer simplification, recyclable or compostable mono-material concepts, and design-for-compostability strategies in order to ensure that sustainable sealing solutions remain compatible with realistic end-of-life scenarios [82,105,106].

In summary, biodegradable and bio-based sealants represent a rapidly evolving class of materials capable of enabling compostable and more sustainable packaging solutions, although their effective implementation still requires careful optimization of sealing performance, multilayer compatibility, processing stability, and end-of-life management. Ongoing advances in polymer blending, nanostructuring, surface modification, and system-level packaging design are expected to progressively expand their industrial applicability while improving compatibility with realistic circularity and compostability pathways.

### 3.4. Non-Thermal and Alternative Sealing Concepts

Non-thermal and alternative sealing concepts have attracted growing interest as potential solutions to reduce energy consumption, expand processing flexibility, and improve recyclability in advanced packaging systems. Unlike conventional heat conductive sealing, which relies on external heat transfer from sealing jaws to the film interface, these approaches exploit localized energy conversion mechanisms to achieve seal formation with reduced thermal input and shorter processing times.

One major class within this category is ultrasonic-based sealing, which converts mechanical vibrations into localized heat generation within the polymer film. Conventional ultrasonic sealing has been established as a viable alternative to conductive sealing for flexible packaging films, exploiting viscoelastic dissipation at the film interface to generate localised heat without external jaw heating, with seam strengths on PA/PE films shown to be comparable to those of thermally sealed references [113]. An innovative alternative is high-intensity focused ultrasound (HIFU) sealing, operating at 500–1500 kHz with smaller vibration amplitudes (1–4 μm). Ref. [114] demonstrated rapid local heating of LDPE films with BOPP and PET carrier layers, achieving temperature rise rates up to 680 K/s and seam strengths comparable to traditional ultrasonic sealing. The process offers reduced equipment complexity, lower noise generation, and minimal film damage, though sealing velocity remains limited to approximately 3 m/min.

Reversible and stimulus-responsive sealing systems represent another important direction [115]. These materials are designed to form seals that can be selectively weakened or released under controlled stimuli such as temperature, humidity, light, or mechanical stress. Ref. [116] developed a fully recyclable, solvent-free photocurable adhesive based on α-lipoic acid derivatives that cures rapidly (30 s) under a wide range of visible wavelengths (400–650 nm), eliminating the need for narrow-spectrum UV sources. The adhesive relies on dynamic disulfide bonds within a covalent adaptable network (CAN) architecture, enabling reversible bonding–debonding cycles without the typical requirement for adding solvents or high-temperature processing. Debonding is achieved using a simple household microwave oven (30 s at low power), which induces molecular vibrations that trigger depolymerization back to the liquid monomeric form. The system demonstrates consistent adhesion strength (>4 MPa) across diverse substrates—including glass, metals, polycarbonate—and retains its mechanical and adhesive performance through at least four recycling cycles, after which photoinitiator replenishment is required due to photobleaching. The adhesive’s transparency, high refractive index, and robustness in wet conditions further expand its potential for optical applications and underwater systems, illustrating its suitability for circular packaging and repairable electronics. A complementary approach specifically targeting multilayer food packaging has been demonstrated by [117], who developed a reusable polyurethane adhesive incorporating thermoreversible Diels-Alder dynamic covalent bonds, enabling up to 20 bonding–debonding cycles triggered remotely within seconds via NIR-absorbing nanoparticles.

Similarly, Ref. [118] reviewed recent advances in smart polymeric adhesives with on-demand reversible switchability based on stimuli-responsive materials, including those utilizing dynamic covalent bonds and supramolecular interactions. These systems can be triggered by various external stimuli such as temperature, light, electricity, magnetism, or chemical agents, with multi-stimuli-responsive adhesives showing particular promise for achieving enhanced versatility and addressing the switchability conflict between high adhesion strength and ease of detachment. While not yet implemented in real packaging systems, these smart adhesives may enable next-generation circular packaging, with full control over adhesion and detachment cycles.

These developments align with broader trends in sustainable packaging design, where adhesives are no longer seen as permanent bonds but as functional interfaces enabling reuse, repair, and separation [119]. From a packaging perspective, such systems are particularly attractive for applications requiring reclosability, controlled opening, or facilitated separation of layers at end of life.

The main categories of non-thermal and alternative sealing technologies discussed in this section are schematically summarised in Figure 14.

Energy efficiency is a central motivation for non-thermal sealing approaches. By reducing or eliminating the need for high sealing temperatures, these systems can lower energy demand during converting and filling operations, while also reducing thermal stress on heat-sensitive products and substrates. This is particularly relevant for bio-based films and paper-based laminates, which tend to degrade or deform under conventional heat-sealing conditions [82]. In addition, there is an economic benefit: the energy savings resulting from the reduced use of high temperatures allow resources previously allocated to energy consumption to be redirected toward the implementation of innovative technological solutions.

Despite their conceptual advantages, non-thermal sealing systems face significant challenges in terms of robustness and scalability. Seal strength, resistance to mechanical loading, and long-term stability under variable environmental conditions often remain inferior to those achieved by conventional thermoplastic sealants. Moreover, sensitivity to surface cleanliness, humidity, and substrate chemistry can introduce variability that is difficult to manage in high-speed industrial lines [120].

From a system-level perspective, alternative sealing concepts must be evaluated not only on the basis of sealing performance, but also in terms of integration within existing packaging architectures and regulatory frameworks. The introduction of functional coatings or responsive chemistries may affect food-contact compliance, migration behaviour, or compatibility with recycling and composting streams.

In the context of this review, non-thermal and alternative sealing concepts represent emerging and largely exploratory technologies rather than immediate replacements for conventional heat sealing. Their primary value lies in highlighting new directions for reducing energy consumption, enabling smart or reversible packaging functions, and supporting circular design principles. Continued development and validation under industrially relevant conditions will be required to assess their long-term feasibility and to define their role alongside established thermoplastic and biodegradable sealant systems.

## 4. Quantitative Performance Metrics and Comparative Assessment

Quantitative performance metrics provide the essential link between material selection, processing conditions, and functional reliability in packaging adhesives and sealants. While Parts I [1] and the preceding sections of Part II established material classes and technological strategies, this section consolidates measurable performance parameters that enable objective comparison across conventional, bio-based, and advanced systems. Emphasis is placed on metrics directly relevant to industrial converting and package integrity rather than on isolated laboratory indicators. The standardized test methods used to quantify these metrics are summarized in Table 4.

### 4.1. Adhesive Performance Metrics

The performance of packaging adhesives cannot be evaluated through a single parameter, since adhesive reliability depends on the combined interaction between interfacial adhesion, cohesive integrity, processing behaviour, environmental stability, and compatibility with packaging substrates and converting operations [14,132]. In multilayer and flexible packaging systems, adhesive layers are required not only to ensure bonding between materials, but also to preserve laminate integrity under thermal, mechanical, and environmental stresses encountered during converting, storage, transport, and service conditions [2,15].

For this reason, adhesive performance is generally assessed through a combination of mechanical, interfacial, rheological, processing, and environmental metrics. The most relevant parameters include bond strength, peel resistance, cohesive durability, aging stability, processing robustness, and interfacial compatibility. Increasing attention is also directed toward environmental and regulatory aspects such as VOC emissions, migration behaviour, NIAS, food-contact compliance, and compatibility with recycling and circular packaging strategies [4,5,6]. The principal adhesive performance metrics discussed in this section are summarized in Table 5 and schematically illustrated in Figure 14, while representative industrial ranges of selected adhesive properties commonly reported in packaging applications are provided in Table 7.

Bond strength represents one of the most widely used performance indicators and describes the ability of the adhesive interface to transfer mechanical loads between adjacent substrates [14,132]. Depending on the application and testing configuration, bond strength may be evaluated through tensile, lap shear, or peel-based methods. In packaging systems, however, measured values are strongly influenced by substrate chemistry, surface treatment, roughness, wettability, adhesive thickness, curing conditions, and environmental exposure. Consequently, nominal strength values alone are often insufficient to describe the real reliability of adhesive joints within multilayer structures [16].

Peel resistance is particularly important in flexible packaging and laminated systems because it reflects resistance to progressive interfacial delamination during opening, bending, flexural deformation, or repeated handling operations. Unlike simple tensile measurements, peel behaviour is strongly affected by viscoelastic dissipation, interfacial fracture mechanisms, and strain localization within the adhesive layer [7,29]. In many packaging laminates, peel performance therefore provides a more representative indication of practical durability than bulk tensile strength alone.

Another critical parameter is cohesive durability, which describes the ability of the adhesive layer to maintain mechanical integrity under long-term loading and environmental exposure. Cohesive degradation may occur through creep, stress relaxation, fatigue, hydrolysis, thermo-oxidative degradation, or moisture-induced plasticization [17,19]. In multilayer packaging, long-term durability is particularly important because laminate failure frequently originates from progressive interfacial weakening rather than from catastrophic fracture of the substrates themselves.

Aging stability is closely related to cohesive durability and includes resistance to humidity, oxygen exposure, ultraviolet radiation, sterilization treatments, thermal cycling, and aggressive food-contact environments. Hydrothermal exposure may progressively alter polymer morphology, interfacial interactions, and adhesive crosslink density, leading to reduced bonding stability and increased risk of delamination [17]. These effects are especially relevant for retortable, refrigerated, or high-barrier food packaging systems subjected to severe service conditions.

Beyond mechanical and environmental resistance, processing robustness represents a key requirement for industrial packaging applications. Adhesive systems must remain compatible with high-speed converting operations while maintaining stable rheological behaviour and reproducible interfacial performance. Important processing-related parameters therefore include viscosity, open time, curing kinetics, setting behaviour, coating uniformity, wetting capability, and sensitivity to temperature or humidity fluctuations during application [2,16]. Adhesives characterized by broad processing windows generally offer improved industrial robustness and reduced sensitivity to line variability.

Interfacial compatibility additionally plays a decisive role in multilayer packaging structures involving chemically dissimilar substrates. Many packaging laminates combine materials with substantially different polarity, crystallinity, thermal expansion behaviour, or surface energy. Adhesive formulations and tie-layer systems must therefore ensure sufficient wetting and stress transfer across heterogeneous interfaces while preserving barrier continuity and mechanical stability during converting and service [23,24].

As summarised in Table 6, migration and NIAS concerns differ substantially across adhesive chemistries: while general regulatory frameworks (e.g., Regulation (EC) No. 1935/2004) apply uniformly across all food-contact materials, the specific migrants of concern, analytical detection strategies, and mitigation approaches are strongly dependent on the chemistry and formulation complexity of each adhesive family.

**Table 6:** Food-contact safety and migration considerations across the main adhesive families discussed in this review. The table links each adhesive family to representative potential migrants and NIAS sources, associated regulatory or safety concerns, typical analytical detection approaches, and mitigation strategies discussed in the literature, distinguishing general regulatory issues from chemistry-specific risks.

Adhesive Family	Main Potential Migrants/NIAS Sources	Representative Regulatory/Safety Concern	Typical Analytical Detection Approach	Mitigation Strategies	Reference(s)
Polyurethane (reactive/laminating)	Residual isocyanate monomers, urethane by-products, catalysts	Isocyanate hazard (REACH Annex XVII training threshold), migration into food simulants	GC-MS/LC-MS for residual monomers; simulant migration testing	Low-monomer/solvent-free formulations, NIPU alternatives, curing optimisation	[20,21,22]
Conventional hot-melt (EVA/wax/tackifier-based)	Tackifiers, waxes, mineral-oil-derived fractions (MOAH/MOSH), antioxidants	NIAS from multicomponent formulations; sensory/toxicological concern	GC-MS for MOAH/MOSH fractions	Recycled/bio-based wax fractions, reduced mineral-oil content, reformulation	[30]
Acrylic and waterborne systems	Residual monomers, surfactants, coalescing agents	Generally lower-risk profile; VOC-related concerns	Chromatographic screening for residual monomer content	Low-VOC/waterborne reformulation, crosslinking optimisation	[18,19]
Modified polyolefin tie layers	Grafting agents (e.g., maleic anhydride residues)	Low migration risk given low polarity/food-contact history	Standard polyolefin migration testing	Optimised grafting degree, minimised tie-layer thickness	[23]
Bio-based/compostable (protein, polysaccharide, bio-derived polyester)	Plasticisers, residual monomers, biomass-derived additives, nanofillers	Bio-based origin does not guarantee low migration; case-by-case assessment needed	Chromatographic/mass-spectrometric screening of oligomers and additives	Careful additive selection, compostability certification (EN 13432 [9]/ASTM D6400 [10]) at package level	[6,32,33]
Functional/hybrid (conductive, nanostructured, active)	Conductive fillers, nanoparticles, reactive/antimicrobial additives	Migration and toxicological profile of nanomaterials not yet fully standardised	Emerging nanomaterial-specific analytical protocols	Careful formulation optimisation, case-by-case toxicological assessment	[60,62,63]

MOSH (Mineral Oil Saturated Hydrocarbons), MOAH (Mineral Oil Aromatic Hydrocarbons).

Figure 15 schematically illustrates the principal performance metrics commonly used to evaluate adhesive systems in packaging applications, including mechanical integrity, peel behaviour, aging resistance, processing robustness, and interfacial compatibility.

Additional packaging-oriented performance indicators may include tack behaviour, thermal and hydrothermal resistance, flex-crack durability, barrier preservation capability, and compatibility with debonding or recycling strategies, depending on the intended packaging architecture and service conditions. These emerging parameters are becoming increasingly relevant as packaging systems evolve toward lightweight multilayer structures, recyclable mono-material concepts, and circular packaging strategies.

Reported adhesive performance values in the literature are frequently obtained using different testing geometries, substrate combinations, conditioning protocols, environmental conditions, and loading configurations. Consequently, direct quantitative comparison across adhesive families and independent studies should be interpreted with caution, particularly in flexible and multilayer packaging applications where interfacial behaviour strongly depends on substrate-specific interactions and converting conditions.

Representative industrial ranges of selected adhesive performance metrics commonly reported in packaging applications are summarized in Table 7. These values should be interpreted as indicative trends because adhesive behaviour strongly depends on substrate combinations, specimen geometry, environmental exposure, and testing methodology.

**Table 7:** Representative industrial ranges of selected adhesive performance metrics in packaging applications. The reported values represent indicative industrial ranges commonly encountered in packaging systems. Actual adhesive performance strongly depends on substrate combinations, testing geometry, curing conditions, environmental exposure, and processing methodology.

Performance Metric	Representative Industrial Range	Common Units	Main Influencing Variables	Representative Reference(s)
Bond strength	~2–20 (aggregate estimate, not attributed to a single study)	MPa	Adhesive chemistry, substrate type, curing conditions	[133,134,135]
Peel resistance	5.3–7.5(15 mm specimen); >6 (25 mm specimen)	N/15 mm; N/25 mm	Laminate structure, viscoelastic behaviour, peel angle	[17,19]
Open time	Seconds–tens of minutes	s/min	Formulation, viscosity, ambient conditions	[136,137]
Service temperature	~−20 to 121	°C	Packaging application and environmental exposure	[138,139]
VOC emissions	Low–high	Qualitative	Solvent content and formulation type	[140]
Moisture sensitivity	Limited–high	Qualitative	Polymer polarity and hydrothermal stability	[141,142]

*Note: Peel resistance values are anchored to representative studies and reported with their original specimen geometry (Ref.* [17]*: 15 mm wide T-peel specimens per GB/T 41168-2021; Ref.* [19]*: 25 mm wide peel specimens per ASTM D1876-08); the two values are not directly comparable due to differing specimen widths and substrate combinations.*

Once the principal adhesive performance metrics have been defined, the following discussion compares representative adhesive families currently used in packaging applications in terms of mechanical reliability, processability, durability, interfacial compatibility, and sustainability-related constraints. Rather than reproducing the complete chemical taxonomy discussed in Section 2, the following comparative assessment groups adhesive technologies into representative performance-oriented families sharing similar interfacial behaviour, processing characteristics, and durability trends commonly encountered in packaging applications.

#### 4.1.1. Polyurethane and Reactive Laminating Systems

Polyurethane-based laminating adhesives remain the industrial benchmark for high-performance flexible and multilayer packaging because they combine strong interfacial adhesion, broad processing windows, and high durability under demanding service conditions [14,16]. Their widespread use in food, pharmaceutical, and technical packaging applications derives from the possibility of tailoring molecular architecture, curing kinetics, flexibility, and polarity in order to bond chemically dissimilar substrates such as polyethylene, polypropylene, PET, polyamide, EVOH, aluminum foils, and paper-based materials.

Consistent with Section 2.1, polyurethane laminating systems generally provide superior cohesive durability and hydrothermal stability compared with waterborne or bio-based families—an advantage that is especially relevant where adhesive failure originates at the interlayer interface rather than within the substrate itself [17].

From a processing standpoint, solvent-based, solvent-free, and waterborne PU technologies accommodate high-speed converting operations with reproducible coating quality and comparatively wide curing tolerances, remaining less sensitive than emerging bio-based systems to fluctuations in humidity, temperature, or substrate variability [2,19]. This robustness, combined with the polarity and crosslink-density tunability already discussed in Section 2.1, explains why PU systems remain dominant in retortable, vacuum, and high-barrier flexible laminates.

As discussed in Section 2.1, isocyanate-related regulatory constraints [20] and VOC/migration concerns remain the principal environmental limitations of conventional PU systems. In multilayer laminates, these same strong and permanent bonds that ensure reliability during service also hinder selective delamination, reducing recyclability [4,5].

These transition strategies, together with reversible and debondable PU interfaces, are examined in detail in Section 5.

#### 4.1.2. Acrylic, Waterborne, and Pressure-Sensitive Systems

As introduced in Section 2.1, compared with polyurethane laminating systems, acrylic and waterborne adhesives generally prioritize processability, surface compatibility, and environmental profile over maximum mechanical durability under severe service conditions [2,18].

Their tack behaviour, optical clarity, and aging resistance, make pressure-sensitive acrylic systems particularly suited to high-speed labeling operations requiring rapid adhesion under low applied pressure [18].

Acrylic and waterborne systems exhibit lower hydrothermal stability and retort resistance than PU systems [26]. Their processing robustness and compatibility with recyclable paper-based architectures nonetheless make them the preferred choice for labels, paper laminates, and resealable systems, rather than for retortable multilayer barrier laminates.

Current development therefore focuses on low-migration, bio-based acrylic hybrids capable of improving durability while preserving the favourable environmental profile of these systems.

#### 4.1.3. Hot-Melt Adhesive Systems

Hot-melt adhesives (HMAs) combine rapid, solvent-free processing with compatibility with high-speed converting lines, bonding through thermoplastic melting and solidification rather than reactive curing [27,28].

HMA rheology is tailored through tackifiers, waxes, and stabilizing additives that govern wetting, open time, and cohesive integrity; this formulation flexibility explains their extensive use in carton sealing, labeling, case/tray assembly, and hygiene-related packaging [28].

Their viscoelastic dissipation behaviour allows for HMAs to accommodate local deformation and contributes to package integrity during transport and handling [29].

As noted in Section 2.1, the thermoplastic (non-crosslinked) nature of conventional HMAs limits their long-term hydrothermal stability compared with reactive polyurethane laminating adhesives [27].

Sustainability efforts for HMAs aim to preserve industrial robustness while improving circularity compatibility [28,31].

Compared with polyurethane laminating systems, HMAs generally provide superior processing speed and simpler application routes, but lower thermal resistance and reduced durability under severe service conditions. Their industrial relevance therefore derives primarily from process integration efficiency, production speed, and broad applicability across conventional packaging operations rather than from maximum structural or hydrothermal performance.

#### 4.1.4. Bio-Based and Compostable Adhesive Systems

Bio-based and compostable adhesives combine renewable origin and end-of-life compatibility, generally at the expense of long-term durability [32,34].

As discussed in Section 2.2.1, moisture sensitivity and limited thermal resistance translate into lower bond strength and hydrothermal stability relative to polyurethane laminating systems, restricting use in high-barrier or retortable applications [42,43].

Processing robustness also varies significantly among bio-based formulations. Many natural macromolecular systems exhibit narrower processing windows and higher sensitivity to humidity, temperature fluctuations, and microbial degradation than conventional synthetic adhesives. Viscosity control, rheological stability, and storage durability may therefore represent important industrial challenges. Current developments increasingly focus on crosslinking strategies, nanoparticle reinforcement, polyelectrolyte complexation, and hybridization approaches aimed at improving water resistance, cohesive integrity, and processability while preserving renewable content and compostability [34,39].

Sustainability assessment, however, requires considering industrial scalability, feedstock sourcing, and compatibility with existing recycling or composting infrastructures, not bio-based content alone. Compared with conventional synthetic adhesives, bio-based systems therefore offer a clear circularity advantage but still face important challenges in durability, hydrothermal resistance, and large-scale industrial implementation.

#### 4.1.5. Comparative Considerations and Performance Trade-Offs for Adhesives

The comparative analysis of the principal adhesive families used in packaging shows that no single adhesive technology can simultaneously maximize bonding performance, processing robustness, environmental resistance, substrate compatibility, regulatory compliance, and end-of-life compatibility. Adhesive selection therefore represents a multidimensional optimization process, in which mechanical durability, processability, interfacial adhesion, sustainability profile, and circularity constraints must be balanced according to the specific packaging architecture [4]. As detailed in the preceding sections, this balance shifts progressively from polyurethane systems, which maximize durability at the expense of circularity, to bio-based and compostable systems, which invert this trade-off by prioritizing renewable content and end-of-life compatibility over mechanical performance, with acrylic/waterborne and hot-melt systems occupying an intermediate position favouring processability and environmental profile.

These trends are summarized in Table 8, which provides a qualitative comparison of the main performance attributes of the representative adhesive families discussed in this section. The table is intended as a performance-oriented synthesis rather than a quantitative ranking, since adhesive properties strongly depend on substrate combinations, curing conditions, testing methods, environmental exposure, coating thickness, and converting parameters.

The comparative trends reported in Table 8 highlight the growing importance of interfacial compatibility and end-of-life management as design constraints in adhesive selection, providing the performance-oriented basis for the circular, reversible, and design-for-disassembly strategies examined in the following section.

### 4.2. Sealant Performance Metrics

Unlike adhesive layers, whose primary role is bonding between substrates, sealant layers must simultaneously ensure closure integrity, hermeticity, mechanical cohesion, leak prevention, and barrier preservation under highly dynamic industrial sealing conditions [7,66].

The performance of packaging sealants is therefore governed by a combination of thermal, mechanical, rheological, and barrier-related parameters that determine both processability during sealing and long-term package reliability. The most important metrics include seal strength, seal initiation temperature (SIT), hot-tack strength, sealing window, hermeticity, contamination tolerance, hydrothermal resistance, and compatibility with recycling or composting strategies. The principal sealant performance metrics discussed in this section are summarized in Table 9 and schematically illustrated in Figure 15, while representative industrial ranges of selected sealant properties commonly reported in packaging applications are provided in Table 11.

Seal strength represents the most widely used parameter for evaluating sealant performance and describes the mechanical resistance of the sealed region under tensile or peel loading conditions [66]. In flexible packaging, seal strength directly influences package integrity during filling, handling, stacking, transport, and opening operations. In addition, different failure modes—including adhesive failure, cohesive failure, tearing failure, or substrate deformation—may substantially influence the interpretation of seal-strength data [7].

Seal initiation temperature (SIT) is another critical industrial parameter and defines the minimum temperature at which a seal with acceptable mechanical integrity can be formed. Low SIT values are generally advantageous because they reduce energy consumption, increase packaging speed, and minimize thermal damage to heat-sensitive materials [7]. However, excessively low sealing temperatures may also compromise seal uniformity and long-term durability if insufficient polymer interdiffusion occurs across the interface [66].

Hot-tack strength describes the resistance of the seal immediately after formation, while the polymer interface is still partially molten and before complete cooling or crystallization occurs [143]. This parameter is particularly important in high-speed packaging operations, where filled packages may be subjected to mechanical stresses shortly after sealing. Insufficient hot-tack behaviour may lead to premature seal opening, leakage, or package deformation during industrial processing.

The sealing window represents the range of temperature, pressure, and dwell-time conditions under which reliable seals can be consistently produced. Broad sealing windows are generally associated with improved process robustness and reduced sensitivity to line variability, substrate thickness fluctuations, or contamination effects [66]. Narrow sealing windows, by contrast, may increase the risk of defective seals and reduce industrial productivity.

Beyond mechanical performance, hermeticity and barrier continuity are essential requirements in food and pharmaceutical packaging. Even when acceptable seal strength is achieved, localized defects, channel formation, incomplete wetting, or contamination within the seal area may compromise gas and moisture barrier performance, thereby reducing shelf life and product safety [7,144]. Seal quality must therefore be evaluated not only in terms of mechanical resistance, but also in relation to leakage prevention and barrier preservation.

Contamination tolerance additionally represents a critical industrial requirement because sealing operations frequently occur in the presence of powders, liquids, fats, condensates, or particulate residues originating from the packaged product itself. Sealants capable of maintaining acceptable seal integrity under contaminated conditions are therefore particularly valuable in food packaging applications involving complex filling operations [66].

Hydrothermal resistance is especially important in retortable, sterilizable, refrigerated, or high-humidity packaging systems. Exposure to heat, steam, moisture, or sterilization treatments may alter seal morphology, interfacial adhesion, crystallinity, and mechanical behaviour, leading to progressive weakening or seal failure [7]. These effects become particularly relevant in biodegradable or compostable sealant systems, which often exhibit greater moisture sensitivity than conventional polyolefin-based materials.

Figure 16 schematically illustrates the principal performance metrics commonly used to evaluate sealant systems in packaging applications, including seal strength, seal initiation temperature, hot-tack behaviour, sealing window, hermeticity, contamination tolerance, hydrothermal resistance, and circularity compatibility.

Finally, sealant evaluation increasingly includes circularity-related considerations associated with recyclability, mono-material packaging architectures, compostability, and compatibility with future design-for-recycling strategies [4,54]. Conventional sealant layers often improve package integrity but may complicate mechanical recycling because of material incompatibility or multilayer complexity. Consequently, growing attention is directed toward sealant systems capable of balancing sealing performance with end-of-life compatibility and sustainable packaging requirements.

As summarised in Table 10, food-contact and migration concerns also differ substantially across sealant families, mirroring the analysis presented for adhesives in Table 6.

Additional packaging-oriented performance indicators may include seal-through capability, puncture resistance near the seal region, optical appearance, machinability, anti-blocking behaviour, and compatibility with modified-atmosphere packaging (MAP) operations, depending on the intended packaging application and processing conditions.

Reported sealant performance values in the literature are strongly influenced by test methodology, specimen geometry, sealing equipment, dwell time, cooling conditions, and substrate structure. Consequently, direct quantitative comparison across sealant families and independent studies should be interpreted with caution, particularly in flexible multilayer packaging systems where sealing behaviour depends strongly on polymer composition and processing conditions.

Representative industrial ranges of selected sealant performance metrics commonly reported in packaging applications are summarized in Table 11. These values should be interpreted as indicative trends because sealant behaviour strongly depends on polymer composition, sealing equipment, dwell time, pressure, cooling conditions, substrate architecture, and testing methodology.

**Table 11:** Representative industrial ranges of selected sealant performance metrics in packaging applications, anchored to representative studies where possible. The reported values represent indicative industrial ranges commonly encountered in flexible and multilayer packaging systems. The retort/hydrothermal resistance value reflects a standard industrial reference condition rather than a single study. Actual sealant performance strongly depends on polymer chemistry, sealing temperature, dwell time, pressure, cooling conditions, substrate architecture, contamination level, and testing methodology.

Performance Metric	Representative Industrial Range	Common Units	Main Influencing Variables	Representative Reference(s)
Seal strength	~0.5–1.1	N/mm	Polymer chemistry, sealing temperature, dwell time, pressure	[7]
Seal initiation temperature (SIT)	~75–175	°C	Melting behaviour, crystallinity, sealant composition	[7]
Hot-tack strength	~0.4–0.65	N/mm	Melt strength, crystallization rate, cooling behaviour	[7]
Sealing window	Narrow (<50) to broad (>120)	°C	Polymer formulation, thermal tolerance, equipment stability	[7]
Retort/hydrothermal resistance	Up to ~121	°C	Multilayer architecture, sealant chemistry, exposure time	[138,139]
Contamination tolerance	Low–high	Qualitative	Product residues, sealant wetting, rheology	[7,66]
Circularity compatibility	Limited–excellent	Qualitative	Mono-material design, compostability, recyclability	[4,54]

Once the principal sealant performance metrics have been defined, the following discussion compares representative sealant families currently used in packaging applications in terms of sealing efficiency, hot-tack behaviour, mechanical resistance, barrier preservation, hydrothermal stability, and sustainability-related constraints. Rather than reproducing the complete material classification discussed in previous sections, the following comparative assessment groups sealant technologies into representative performance-oriented families characterized by similar sealing behaviour, processing characteristics, and durability trends commonly encountered in packaging applications.

#### 4.2.1. Polyolefin-Based Sealant Systems

Polyolefin-based sealants, particularly polyethylene (PE), low-density polyethylene (LDPE), linear low-density polyethylene (LLDPE), cast polypropylene (CPP), and related copolymers, remain the dominant industrial sealant materials for flexible packaging because they combine good sealability, broad processing windows, chemical resistance, and relatively low cost [7,66]. Their widespread adoption derives primarily from their ability to generate reliable hermetic seals under high-speed industrial converting conditions while maintaining adequate mechanical and barrier performance across a broad range of food and consumer packaging applications.

As discussed in Section 3.1, LDPE offers a low SIT and broad sealing tolerance, while PP requires higher sealing temperatures and tighter process control [7,66,143]. Within the polyethylene family, LLDPE further provides improved toughness and puncture resistance due to its higher degree of chain entanglement compared with conventional LDPE.

From a sealing-performance perspective, polyolefin systems are generally characterized by good seal strength and broad industrial processability. Their thermoplastic behaviour allows for rapid interdiffusion and entanglement formation across the seal interface, which is essential for achieving strong hermetic seals during short industrial sealing cycles. At the same time, seal performance remains strongly dependent on crystallinity, molecular weight distribution, cooling rate, and coextrusion architecture.

Nevertheless, conventional polyolefin sealants also present some important limitations. Their oxygen-barrier performance is generally limited, requiring integration with additional high-barrier layers such as EVOH or metallized structures in demanding food-packaging applications. In addition, excessive crystallinity or narrow melting transitions may reduce sealing robustness and increase sensitivity to thermal fluctuations during converting operations.

Hydrothermal resistance is generally satisfactory for conventional polyolefin sealants, particularly in polyethylene-based systems used for refrigerated and moisture-sensitive packaging. However, prolonged exposure to elevated temperatures, aggressive sterilization conditions, or repeated thermal cycling may progressively alter seal morphology and interfacial cohesion, especially in thinner multilayer structures.

Another major advantage of polyolefin-based sealants lies in their compatibility with mono-material flexible packaging concepts currently being developed to improve recyclability. Polyethylene-rich sealant layers are increasingly integrated into all-PE recyclable laminates, while polypropylene sealants are employed in recyclable PP-based structures designed to reduce incompatibility between multilayer components [4,54]. Current developments increasingly pursue this goal through advanced copolymer design, metallocene polyolefins, compatibilized mono-material structures, downgauging, and recycled-content incorporation, aiming to preserve the processability and robustness of polyolefin sealants while improving compatibility with circular packaging requirements.

#### 4.2.2. High-Barrier and Multilayer Sealant Structures

As discussed in Section 3.2, high-barrier multilayer sealant systems combine a heat-sealable inner layer (typically LDPE, LLDPE, PP, or ionomers) with barrier components such as EVOH, PVDC, or metallized films, allowing sealing performance and barrier performance to be optimized largely independently [7,66,73].

Multilayer sealant systems additionally require tie layers or compatibilizing adhesive systems to maintain structural integrity between chemically dissimilar materials, with differences in crystallization kinetics among adjacent layers further influencing sealing quality and long-term durability [145].

From a processing standpoint, multilayer sealants often exhibit narrower operational tolerances than conventional mono-material systems because sealing conditions must be optimized while preserving the integrity of adjacent barrier layers. Excessive sealing temperatures may damage barrier coatings or induce distortion, whereas insufficient thermal input may lead to incomplete sealing and reduced hermeticity.

As noted in Section 3.2, the material heterogeneity of these structures continues to hinder efficient mechanical recycling and selective layer separation [4,54,81]. Current developments therefore increasingly address this challenge through advanced coating technologies, plasma treatments, and nanostructured barrier layers designed to reduce dependence on highly heterogeneous multilayer constructions while preserving adequate sealing and barrier performance. Consequently, multilayer flexible packaging remains one of the most problematic packaging streams from a circularity perspective.

Current developments therefore increasingly address this recyclability challenge through advances in coating technologies, plasma treatments, nanostructured barrier layers, and delamination-friendly interface design, aiming to reduce dependence on highly heterogeneous multilayer constructions while preserving adequate sealing and barrier performance.

#### 4.2.3. Biodegradable and Compostable Sealant Systems

As discussed in Section 3.3, biodegradable sealants are commonly based on aliphatic polyesters (PLA, PBS, PBAT, PHBV) or starch-based blends, and generally exhibit narrower sealing windows and greater sensitivity to temperature, humidity, and processing variability than conventional polyolefin sealants [7,50]. Their crystallization behaviour, melt viscosity, and thermal stability may significantly affect seal formation and hot-tack performance, requiring careful optimization of sealing parameters for reliable industrial processability [50].

Consequently, optimization of sealing parameters becomes particularly important for achieving reliable industrial processability.

From a mechanical perspective, biodegradable sealants may provide acceptable seal strength for moderate-performance packaging applications, although hydrothermal resistance and long-term durability often remain lower than those of conventional polyethylene-based systems. Moisture sensitivity and thermo-mechanical instability may progressively reduce seal integrity under refrigerated, humid, or retortable conditions.

Hot-tack behaviour additionally represents a critical challenge in many compostable systems because slower crystallization kinetics and lower melt strength may increase the risk of premature seal opening during high-speed packaging operations [66]. For this reason, biodegradable sealants frequently require formulation optimization through copolymerization, plasticization, multilayer structuring, or incorporation of flexible biodegradable components such as PBAT.

Despite these limitations, biodegradable sealants offer important environmental advantages because they may improve compatibility with compostable packaging streams and reduce persistence in organic waste-management systems. In paper-based compostable packaging, these systems can additionally facilitate development of partially bio-based structures with improved end-of-life compatibility compared with conventional fossil-based laminates [106].

However, the environmental performance of compostable sealants depends strongly on waste-management infrastructure, composting conditions, contamination levels, and compatibility with existing recycling streams. In some cases, biodegradable sealants may complicate conventional mechanical recycling if improperly mixed with fossil-based polyolefin streams. Consequently, current research increasingly focuses on balancing compostability, sealing performance, industrial processability, and compatibility with realistic end-of-life scenarios.

Recent developments include bio-based hot-melt sealants, compostable multilayer systems, PBS- and PBAT-rich flexible sealants, reactive biodegradable interfaces, and mono-material biodegradable packaging concepts designed to improve seal reliability while maintaining compostability and industrial scalability.

#### 4.2.4. Comparative Considerations and Performance Trade-Offs for Sealants

The comparative analysis of the principal sealant families used in packaging highlights the strong interdependence between sealing performance, industrial processability, barrier preservation, and end-of-life compatibility. As observed for adhesive systems, no single sealant technology simultaneously maximizes all thermal, mechanical, barrier, and sustainability-related requirements.

Polyolefin-based sealants remain the industrial benchmark thanks to their broad sealing windows, low SIT, strong hot-tack behaviour, and compatibility with mono-material recycling strategies [7,66].

High-barrier multilayer structures provide the highest level of environmental protection and shelf-life extension but present the greatest recycling challenges due to their structural heterogeneity, while biodegradable and compostable sealants offer the most favourable end-of-life profile at the expense of hydrothermal resistance, sealing robustness, and processing stability [7,50]. Their successful implementation therefore depends strongly on balancing sustainability objectives with realistic packaging-performance requirements.

Contemporary sealant engineering must therefore simultaneously consider processability, package integrity, barrier continuity, recyclability, compostability, regulatory compliance, and compatibility with evolving circular packaging strategies.

Table 12 summarizes the principal performance trends associated with the representative sealant families discussed in this section. The comparison is qualitative and intended as a performance-oriented synthesis because reported sealing properties strongly depend on polymer composition, multilayer architecture, sealing equipment, test methodology, and processing conditions. Consequently, the trends summarized here should be interpreted as representative comparative tendencies rather than as universal quantitative rankings of sealant technologies.

As summarised in Table 12, technology readiness and regulatory maturity vary independently of sealing performance, particularly for biodegradable systems, which remain at an earlier industrial stage despite favourable circularity compatibility while Table 10 summarises the corresponding food-contact and migration considerations.

The comparative considerations discussed in this section provide the performance-oriented basis for the emerging recyclable, compostable, and design-for-disassembly sealing strategies examined in the following section.

**Table 12:** Comparative performance and maturity trends of the principal sealant families used in packaging applications, including technology readiness, scalability, and regulatory maturity alongside sealing performance metrics.

Sealant Family	Typical Application Area	Seal Strength	SIT/Sealing Efficiency	Hot-Tack Behaviour	Sealing Window	Hydrothermal Resistance	Barrier Compatibility	Circularity Compatibility	Technology Readiness	Scalability	Relative Cost	Regulatory Maturity	Representative References
Polyolefin-based sealants	Flexible food packaging, mono-material/recyclable PE- or PP-rich structures, general-purpose heat-sealed pouches	High	Excellent	High	Broad	High	Moderate	Good	Commercial/mature	High	Low	Established, standard food-contact frameworks	[7,66]
High-barrier multilayer sealants	Retort pouches, vacuum packaging, MAP structures, thermoformed trays, long-shelf-life food packaging	High	Moderate	Moderate–high	Moderate	High	Excellent	Limited	Commercial/mature	High	Moderate–high	Established performance; recyclability scrutiny increasing under PPWR	[4,54]
Biodegradable and compostable sealants	Compostable flexible films, sachets, mono-material compostable laminates, paper-based compostable packaging	Moderate	Moderate	Limited–moderate	Narrow–moderate	Limited	Moderate	Excellent	Pilot-to-emerging	Limited–moderate	Moderate–high	Certification frameworks exist (EN 13432 [9]/ASTM D6400 [10]) but whole-package compatibility remains case-specific	[7,50]

## 5. Circularity-Oriented Adhesive and Sealant Systems

The progressive transition toward circular packaging systems is fundamentally redefining the role of adhesive and sealant interfaces within modern packaging architectures. Although adhesives often represent only a minor fraction of the total package mass, their presence may strongly influence recyclability, compostability, material recovery, and compatibility with circular-economy strategies [4,54,119]. Historically optimized mainly to maximize bonding performance, sealing reliability, and long-term durability, interfacial technologies are now increasingly evaluated according to these end-of-life criteria as well.

Table 13 summarises the technology readiness, scalability, cost, and regulatory maturity of the main emerging non-thermal, debondable, and smart interfacial technologies discussed in this and the preceding section, distinguishing pilot-scale developments from proof-of-concept laboratory demonstrations.

This transition is particularly important in flexible multilayer packaging, where adhesive interlayers, sealant layers, coatings, and tie-layer systems frequently represent critical barriers to efficient recycling and material separation. Strong and permanent interfacial bonding, while beneficial for mechanical integrity and barrier preservation during service, may hinder selective delamination and reduce the quality and economic value of recovered material streams. Consequently, increasing attention is directed toward interfacial systems capable of balancing packaging reliability with end-of-life compatibility and design-for-recycling requirements. These developments reflect the broader transition from conventional permanent bonding approaches toward circularity-oriented interfacial engineering concepts in which recoverability and dismantling are integrated directly into adhesive design [119].

At the same time, conventional adhesive and sealant technologies remain indispensable for achieving the mechanical durability, sealing efficiency, barrier continuity, and processability required in industrial packaging operations. For this reason, current developments rarely involve complete replacement of established technologies; instead, most strategies focus on progressively adapting them to improve environmental compatibility and facilitate integration into circular material systems, while preserving functional performance and industrial scalability [119].

Within this framework, contemporary research increasingly explores solvent-free and waterborne systems, recyclable mono-material packaging architectures, low-migration formulations, non-isocyanate polyurethanes (NIPUs), recyclable multilayer structures, biodegradable interfaces, debond-on-demand systems, and design-for-disassembly approaches intended to facilitate material recovery and circular packaging integration [18,19,55]. Parallel developments additionally investigate smart and responsive interfacial systems capable of enabling selective delamination, controlled dismantling, traceability, or adaptive packaging functionality under specific service or recycling conditions [149,150].

Figure 17 schematically summarizes the principal transition pathways currently driving the evolution of adhesive and sealant technologies from conventional high-performance systems toward circularity-oriented and multifunctional packaging interfaces.

The following sections examine the principal technological strategies currently being developed to improve the compatibility of adhesive and sealant systems with recycling, compostability, and circular packaging requirements, with particular emphasis on transition pathways that preserve industrial processability and packaging performance while enabling more sustainable end-of-life management.

### 5.1. Transition Strategies for Conventional Adhesives and Sealants

Despite the growing interest in biodegradable and reversible interfacial systems, conventional synthetic adhesives and sealants continue to dominate industrial packaging because of their superior durability, broad processing windows, sealing reliability, and compatibility with high-speed converting operations [7,16]. Current transition strategies therefore focus on progressively reducing the environmental impact of these established systems rather than replacing them outright [151].

One of the most important transition pathways involves the progressive replacement of solvent-intensive technologies with solvent-free, waterborne, and low-VOC systems. Solvent-free polyurethane laminating adhesives are increasingly employed in flexible packaging because they reduce volatile organic compound emissions while maintaining high interfacial strength and compatibility with multilayer structures [19]. Waterborne acrylic and polyurethane dispersions additionally enable lower-emission converting operations and reduced handling risks, particularly in paper-based and flexible packaging applications. These systems are often associated with improved occupational safety and lower environmental impact during manufacturing, although challenges related to drying efficiency, moisture sensitivity, and long-term durability may still remain in demanding applications [16].

Another major transition strategy concerns the development of recyclable mono-material packaging architectures designed to reduce incompatibility between multilayer components. Conventional flexible packaging frequently combines polyethylene, polypropylene, PET, EVOH, aluminum, adhesives, and sealant layers within highly heterogeneous structures that are difficult to separate during recycling [4,54]. Current developments therefore increasingly focus on polyethylene-rich or polypropylene-rich mono-material laminates in which adhesive and sealant systems are optimized to preserve interfacial integrity while maintaining compatibility with existing mechanical recycling streams [151]. A complementary strategy consists of redesigning the interlayer adhesion itself so that multilayer structures can retain adequate performance during use while enabling layer separation during recycling. In this direction, ref. [147] investigated recyclable multilayer packaging concepts based on controlled interlayer adhesion, including localized surface-treatment patterns and water-soluble tie layers designed to facilitate separation during shredding and washing operations.

Within these architectures, tie-layer chemistry and interfacial compatibilization become particularly important. Modified polyolefins, maleic-anhydride-grafted materials, and compatibilized multilayer structures are increasingly employed to improve adhesion between barrier layers and recyclable polyolefin matrices while minimizing contamination of recycled streams [23,24]. At the same time, downgauging strategies and thinner interfacial layers are being explored to reduce overall material consumption and facilitate recyclability without compromising package integrity. Similar approaches are already being implemented industrially through adhesive-assisted pallet stabilization systems, multipack bonding technologies, paper-based tear tapes, and recyclable barrier-coated paper packaging solutions designed to reduce packaging waste and improve recyclability [151].

Growing attention is also directed toward low-migration and food-contact-safe formulations capable of reducing the release of volatile compounds, residual monomers, plasticizers, mineral oils, and NIAS into packaged products [5,30]. This aspect is especially important in food and pharmaceutical packaging, where regulatory compliance increasingly intersects with sustainability and circularity considerations.

Additional developments involve partially bio-attributed synthetic systems and NIPU technologies designed to reduce dependence on hazardous monomers and petrochemical feedstocks while preserving the mechanical and processing advantages of polyurethane chemistry [18,22]. Although many of these systems are still under industrial optimization, they represent an important transitional step between conventional fossil-based adhesive technologies and fully biodegradable or circular interfacial systems.

For sealants, similar transition strategies include development of recyclable polyolefin-rich sealing layers, compostable sealants compatible with biodegradable packaging, lower-temperature sealing systems aimed at reducing energy consumption, and advanced sealant formulations capable of preserving sealing reliability in downgauged or mono-material packaging structures [7,50].

### 5.2. Design-for-Disassembly and Debondable Interfaces

One of the most promising strategies for improving the circularity of packaging systems involves the development of adhesive and sealant interfaces specifically engineered to facilitate selective separation, controlled delamination, and material recovery at end of life. Recent research increasingly focuses on trigger-responsive multilayer adhesive systems capable of maintaining strong interfacial performance during service life while enabling on-demand debonding during recycling operations [55,148]. These approaches are closely associated with dismantlable or debond-on-demand adhesive concepts originally developed to support zero-waste manufacturing and circular material reintegration strategies [55,119], and more broadly with design-for-disassembly (DfD) principles, in which packaging structures are conceived not only for performance during service, but also for efficient dismantling and recycling after use [55,115]. In packaging-specific research, this concept has recently been translated into multilayer packaging designs based on controlled interlayer adhesion, where the interface is engineered either to reduce the adhered area or to introduce a removable tie layer without abandoning multilayer functionality [147].

To address the recyclability limitations of permanent bonding systems discussed above, debondable and reversible interfaces have been developed to maintain sufficient adhesion during service while enabling controlled weakening or selective delamination under predefined external stimuli.

Dismantlable adhesive systems are commonly classified according to the external stimulus responsible for debonding, including thermal, electrical, chemical, mechanical, and radiation-triggered mechanisms [119]. Among the most extensively investigated approaches are thermally reversible adhesive systems based on dynamic covalent chemistry, particularly Diels–Alder networks and vitrimer-like architectures [55,152]. In these systems, reversible covalent bonds allow interfacial strength to decrease under controlled thermal activation, enabling selective separation of packaging layers without severe substrate damage. Such approaches are particularly attractive for multilayer flexible packaging, where conventional recycling is often limited by the permanent integration of chemically incompatible materials.

Thermally debondable systems may additionally incorporate expandable particles, gas-releasing additives, or phase-transition mechanisms capable of generating interfacial stresses during heating, thereby promoting layer separation [115]. Other approaches rely on solvent-triggered, moisture-triggered, or pH-responsive interfacial weakening mechanisms that facilitate dismantling under controlled recycling conditions. Light-responsive adhesive systems have recently emerged as particularly attractive solutions for multilayer food packaging because they may allow clean delamination without solvents, aggressive chemicals, or high mechanical energy input. Ref. [148] developed a photosensitive thermoplastic tie layer based on coumarin-derived photolabile groups capable of preserving strong PE–PA adhesion during service while undergoing controlled molecular fragmentation under UV irradiation, thereby enabling residue-free layer separation. From a broader debonding-on-demand perspective, light-triggered nanocomposite adhesives have also been designed to operate under milder activation conditions. Ref. [153] developed an NIR-responsive PNIPAM/polydopamine nanocomposite adhesive in which localized photothermal heating induces volumetric shrinkage of the adhesive layer, loss of interfacial contact, and residue-free debonding under ambient conditions. Although not specifically developed for multilayer packaging, this approach is relevant as a mechanistic example of solvent-free and non-destructive optical debonding.

A particularly relevant packaging-oriented example is the use of a water-soluble PVOH tie layer in multilayer laminates. Ref. [147] showed that this strategy can provide adhesion levels comparable to commercial multilayer packaging while allowing layer separation after shredding and immersion in water. In their Design B configuration, the average peel force was 3.45 ± 0.3 N, corresponding to a peel strength of approximately 0.14 N/mm, and the layers separated after immersion, with complete separation observed after prolonged exposure.

Mechanically activated debonding systems have also attracted growing attention. Ref. [146], for example, developed microcapsule-containing adhesive systems in which mechanical compression ruptures embedded capsules and releases plasticizing agents capable of dramatically reducing adhesive stiffness and promoting interfacial separation. Unlike thermal activation strategies, mechanically triggered debonding may allow localized and highly controlled dismantling without requiring elevated temperatures or aggressive chemical treatments.

Another important development concerns removable and wash-off adhesive technologies already employed in label applications for PET bottles and reusable glass containers. These systems represent one of the earliest industrial examples of design-for-disassembly concepts in packaging because they enable separation of labels during washing and recycling operations while maintaining adequate adhesion during service [65]. Current research increasingly aims to extend similar principles to more complex multilayer flexible packaging systems.

Figure 18 schematically illustrates the principal debonding and design-for-disassembly mechanisms currently investigated for circular packaging applications, highlighting how external stimuli may enable selective layer separation and improved material recovery.

Despite their significant potential, debondable interfaces also present important technical challenges. A central difficulty lies in balancing operational robustness with selective disassembly capability. Adhesives that debond too easily may compromise package integrity during transport or storage, whereas excessively stable systems may require activation conditions incompatible with realistic industrial recycling operations [154]. Consequently, debonding triggers must be carefully aligned with practical end-of-life scenarios and existing recycling infrastructures rather than optimized exclusively under laboratory conditions.

Another challenge concerns compatibility with food-contact requirements, large-scale converting operations, and long-term environmental stability. Many dynamic or reversible chemistries remain more complex and costly than conventional industrial adhesive systems, and their large-scale integration into high-speed packaging processes is still under development.

Nevertheless, design-for-disassembly strategies increasingly represent one of the most important conceptual shifts in packaging interfacial engineering, integrating recoverability directly into interfacial design rather than treating end-of-life separation as an external recycling problem [119,148].

### 5.3. Smart and Functional Circular Interfaces

Beyond reversible and debondable systems, growing attention is also directed toward smart and multifunctional adhesive–sealant interfaces capable of combining conventional bonding or sealing functions with additional responsive, sensing, or adaptive properties relevant to circular packaging systems [149,150]. In these approaches, the interface is no longer considered a passive joining layer, but rather an active functional component capable of interacting with processing conditions, environmental stimuli, digital technologies, or recycling operations.

Many smart adhesive systems rely on dynamic bonding mechanisms, supramolecular interactions, or reversible covalent networks capable of modulating interfacial behaviour under external stimuli such as temperature, light, humidity, electric fields, magnetic fields, or chemical agents [118]. Depending on the formulation design, these mechanisms may enable reversible adhesion, adaptive sealing behaviour, controlled permeability, repeated bonding–debonding cycles, or selective dismantling during recycling operations.

In packaging applications, some of the most relevant developments concern smart labels, traceability systems, tamper-evident structures, and interfaces integrated with sensing technologies for freshness monitoring, product authentication, or supply-chain management [155,156]. Electrically conductive adhesive systems have additionally been investigated for emerging electronic and intelligent packaging concepts requiring low-temperature assembly of RFID components, printed electronics, sensors, or communication devices [157,158].

From a circularity perspective, these technologies may contribute indirectly to improved waste management and material recovery by enabling digital sorting, tracking of material composition, monitoring of package integrity, or facilitation of automated disassembly processes [149,150]. Smart interfaces may therefore support future integration between packaging materials, recycling infrastructure, and digital circular-economy frameworks.

At the same time, increasing multifunctionality inevitably introduces additional formulation complexity. Conductive fillers, nanoparticles, responsive additives, supramolecular motifs, and sensing components may influence viscosity, curing behaviour, sealing efficiency, migration characteristics, recyclability, and food-contact compatibility. Consequently, many smart adhesive and sealant systems currently operate within narrower mechanical, thermal, and regulatory windows than conventional industrial technologies.

For this reason, most smart and multifunctional interfaces remain associated with niche, high-value, or technologically specialized packaging sectors rather than large-volume commodity packaging. Their industrial implementation is still limited by challenges related to scalability, cost, process integration, long-term stability, and compatibility with existing recycling systems [154].

Figure 19 summarizes the functional evolution of packaging adhesive and sealant interfaces from conventional joining technologies toward circularity-oriented and multifunctional systems integrating recyclability, adaptive response, and intelligent packaging functionalities.

Taken together, these developments illustrate a broader transition in packaging interfacial engineering: adhesives and sealants are progressively evolving from purely structural joining technologies into adaptive interfaces integrating bonding, sealing, sensing, traceability, and disassembly functionalities. The transition strategies, debondable systems, and smart interfaces discussed throughout this section indicate that the future evolution of these technologies will depend not on a single universal solution, but on the progressive integration of performance, processability, recyclability, and circularity considerations into interfacial design itself.

## 6. Future Perspectives and Remaining Challenges

The evolution of adhesive and sealant technologies for packaging is increasingly driven by the need to reconcile high-performance interfacial functionality with circularity-oriented material management. Throughout this review, it has emerged that contemporary adhesive and sealant systems are no longer evaluated solely according to their bonding strength, sealing efficiency, or processing robustness, but also in relation to their compatibility with recycling, compostability, material recovery, and evolving regulatory requirements. Consequently, future developments in packaging interfacial engineering will likely depend on the ability to balance performance, sustainability, industrial scalability, and end-of-life compatibility within increasingly complex packaging architectures.

**Balancing Performance and Circularity**

One of the principal challenges facing next-generation adhesive and sealant systems concerns the intrinsic trade-off between interfacial durability during service and selective separability at end of life. Conventional high-performance adhesives and sealants are typically designed to maximize cohesive integrity, hydrothermal resistance, and long-term stability, but these same characteristics frequently hinder material separation and reduce the recyclability of multilayer packaging structures [4,54]. This conflict is especially evident in flexible multilayer packaging, where permanent adhesive interlayers and heterogeneous sealant architectures complicate selective delamination and recovery of high-purity material streams: interfaces designed for easier disassembly may in turn exhibit reduced hydrothermal stability, narrower processing windows, or lower long-term durability.

A similar trade-off applies to compostable systems, whose end-of-life advantages come at the cost of the moisture sensitivity, thermal stability, and mechanical durability limitations already discussed in Section 3.3 and Section 4 [7,50]. Consequently, future research will increasingly require integrated optimization approaches capable of simultaneously considering sealing efficiency, barrier preservation, recyclability, compostability, and processing robustness rather than focusing on isolated material properties.

The progressive transition toward mono-material packaging architectures additionally introduces new interfacial constraints. While mono-material laminates may facilitate recycling, they often require highly optimized compatibilization strategies to maintain adequate adhesion, barrier continuity, and sealing reliability without relying on highly heterogeneous multilayer structures [23,54]. In this context, the future role of adhesive and sealant systems will increasingly depend on their ability to support packaging simplification without compromising functionality during service.

**Standardization and Testing Challenges**

Another important limitation concerns the lack of harmonized methodologies for evaluating adhesive and sealant performance within circular packaging systems, compounding the substrate-, geometry-, and condition-dependent variability already noted in Section 4 and Section 5. This issue becomes even more critical for emerging circularity-oriented systems such as debondable adhesives, dynamic interfaces, recyclable multilayer structures, and compostable sealants, for which standardized testing methodologies are still limited or absent. For example, no universally accepted protocols currently exist for evaluating selective delamination efficiency, debonding reliability under realistic recycling conditions, or the combined influence of recyclability and interfacial durability within multilayer packaging systems.

For debondable multilayer packaging, T-peel testing combined with adhesive fracture energy analysis has been proposed as a useful approach to distinguish simple peel force from the actual interfacial work of fracture, especially when flexible peel arms undergo bending and plastic deformation [147]. As a result, apparent differences in reported performance may partially reflect methodological variability rather than intrinsic material behaviour.

The increasing complexity of smart, multifunctional, and circularity-oriented interfaces further amplifies these challenges because future packaging systems may require simultaneous evaluation of mechanical performance, barrier preservation, recyclability, migration behaviour, digital traceability, and environmental compatibility. Consequently, future standardization efforts will likely play a central role in enabling reliable comparison, industrial validation, and regulatory acceptance of emerging adhesive and sealant technologies.

**Industrial Scalability and Infrastructure Constraints**

Although many advanced adhesive and sealant concepts demonstrate promising laboratory-scale performance, their large-scale industrial implementation remains constrained by economic, technological, and infrastructural limitations, since packaging manufacturing is optimized for high-speed converting operations with narrow production tolerances and low processing costs [7,16]. This is particularly true for the debondable, stimuli-responsive, and multifunctional interfaces summarised in Table 13, many of which involve complex chemistries or specialized activation mechanisms whose compatibility with coating, lamination, sealing equipment, food-contact regulations, and long-term storage stability has not yet been established at scale.

Infrastructure limitations also strongly influence the practical effectiveness of circular adhesive and sealant systems: recyclable or compostable structures can only deliver real environmental benefits where adequate collection, sorting, and processing infrastructures exist, and—as discussed in Section 3.3.4—this is rarely guaranteed outside industrial composting or advanced sorting facilities [4,54,159]. Future interfacial technologies must therefore be designed in relation to realistic waste-management infrastructures rather than idealized end-of-life scenarios alone.

Cost also remains a decisive factor: conventional polyurethane laminating systems and polyolefin sealants remain dominant not only for performance reasons but also for their relatively low cost and compatibility with existing manufacturing infrastructure, reinforcing the gradual (rather than disruptive) integration pathway already discussed in Section 5.1.

**Regulatory Evolution and Circular Packaging Policies**

Regulatory frameworks are increasingly becoming one of the principal driving forces shaping the future evolution of packaging adhesive and sealant technologies. It is important to distinguish among the different regulatory and normative instruments referenced throughout this review, as they carry different legal weight and serve different functions. Binding legislation—such as Regulation (EC) No. 1935/2004 [12], Regulation (EC) No. 2023/2006 [13], and the Packaging and Packaging Waste Regulation (PPWR) [11]—establishes mandatory safety and design requirements enforceable across the EU. Voluntary technical standards—such as EN 13432 [9] and ASTM D6400 [10]—define test methods and performance thresholds (e.g., for compostability) but are not legally binding per se, although compliance with them may be referenced or required by downstream legislation or contractual specifications. Certification schemes built upon these standards (e.g., EN 13432-based industrial compostability certification) provide third-party verification that a specific product meets the corresponding standard’s criteria. Finally, industry guidelines and classification protocols—such as those issued by CEFLEX or RecyClass—represent voluntary, sector-driven recommendations that support design-for-recycling practices but do not constitute regulatory or certification requirements. Failure to distinguish these categories can lead to overstated compliance claims in both scientific literature and industrial practice.

A further critical point, often overlooked, is that the regulatory or certification status of an individual adhesive or sealant component does not automatically extend to the finished packaging article. Food-contact compliance under Regulation (EC) No. 1935/2004 [12], for instance, must be demonstrated for the complete packaging system under its intended conditions of use, since migration behaviour, layer interactions, and processing history can substantially affect the safety profile of the assembled structure even when each individual material has been separately assessed as compliant. Similarly, compostability certification under EN 13432 [9] applies to the packaging article as a whole rather than to individual layers; a compostable adhesive combined with a non-compostable substrate, printing ink, or barrier coating does not yield a certifiable compostable package. This distinction has direct implications for the interpretation of sustainability and compliance claims discussed throughout this review.

In addition to traditional food-contact and migration requirements, contemporary regulations progressively incorporate recyclability, circularity, extended producer responsibility (EPR), and eco-design considerations into packaging development strategies.

Within the European framework, increasing emphasis is placed on recyclable packaging architectures, reduction of problematic multilayer structures, minimization of hazardous substances, and improved compatibility with circular material flows. Consequently, adhesive and sealant systems may increasingly be evaluated not only according to their immediate functional performance, but also according to their influence on sorting efficiency, recyclability, material contamination, and recovery of secondary raw materials.

At the same time, migration behaviour, NIAS, residual monomers, additives, and degradation products remain critical issues for food-contact packaging applications [5,6]. Future formulations will therefore require increasingly careful balancing between environmental compatibility, chemical safety, processing stability, and regulatory compliance.

Another emerging regulatory challenge concerns verification of compostability and biodegradability claims. Many biodegradable interfacial systems may behave differently depending on industrial composting conditions, marine exposure, soil environments, or recycling contamination scenarios. Consequently, harmonized certification methodologies and more realistic environmental assessment protocols will likely become increasingly important for future packaging systems.

**Emerging Research Directions**

Future research on packaging adhesive and sealant systems will likely increasingly emphasize integrated interfacial engineering approaches in which performance optimization, circularity compatibility, and intelligent functionality are simultaneously addressed at the system level—considering substrates, coatings, barrier layers, adhesives, sealants, and end-of-life management as interconnected design variables rather than isolated material properties.

Particular attention is expected to remain directed toward recyclable mono-material packaging systems, dynamic and reversible interfaces, biodegradable multilayer structures, low-migration formulations, and compatibilized high-barrier architectures capable of balancing shelf-life requirements with recycling compatibility. Dynamic covalent networks, vitrimers, reversible supramolecular systems, and controlled debonding technologies may additionally provide new opportunities for selective disassembly and material recovery in future circular packaging systems.

Smart and digitally integrated interfaces may also become increasingly relevant as packaging technologies progressively incorporate traceability, sensing, authentication, and automated sorting functionalities. In this context, adhesive and sealant layers may evolve from passive joining materials toward multifunctional interfacial platforms capable of simultaneously supporting structural integrity, sealing reliability, information management, and circularity-oriented material flows.

## 7. Conclusions

This two-part review examined adhesives and sealants as system-level technologies that govern the performance and circularity of modern packaging. Part I established the classification framework; Part II extended it to advanced and emerging systems.

Three groups of technologies emerge from this analysis.

Conventional polyurethane laminating adhesives, hot-melt EVA/acrylic systems, and polyolefin-based sealants are the most industrially mature. They offer broad processing windows, established supply chains, and well-defined regulatory pathways.

Bio-based and compostable adhesives, mono-material recyclable architectures, and PBAT-based hot-melts are the most promising emerging technologies. They already show encouraging performance data, but remain limited by moisture sensitivity, cost, and a lack of industrial-scale validation.

Debond-on-demand and stimuli-responsive interfaces represent an early but important shift toward packaging designed for selective separation. Most remain at the laboratory or pilot scale.

Six research gaps must be closed to move these technologies toward industrial adoption: harmonized testing protocols; long-term aging data under realistic conditions; migration studies under real-use conditions; industrial-scale recycling trials; scaled validation of debonding technologies; and life-cycle assessment and scale-up cost data.

Closing these gaps will require coordinated progress across materials chemistry, packaging engineering, recycling infrastructure, and regulation—not materials development alone.

Ultimately, adhesives and sealants are design-critical materials. Their future role depends on their capacity to preserve material value and support the transition to circular packaging systems.

## Figures and Tables

**Table 1:** Performance- and maturity-oriented comparison of adhesive families discussed in Section 2. Qualitative comparison of adhesion performance, resistance, processing compatibility, circularity profile, industrial maturity, scalability, relative cost, and regulatory maturity for the main adhesive families considered in this section.

Adhesive Family	Main Strengths	Main Limitations	Typical Application Area	Processing Compatibility	Circularity Profile	Technology Readiness	Scalability	Relative Cost	Regulatory Maturity
Synthetic PU adhesives	Very high adhesion; high thermal, moisture, and chemical resistance; excellent multilayer performance	Limited recyclability; isocyanate-related concerns; possible migration issues depending on formulation	Retortable and high-barrier flexible laminates; food and pharmaceutical multilayer packaging	Excellent	Limited–moderate	Mature	High	Moderate–high	Established, but under increasing scrutiny (REACH diisocyanate training requirements since 2023)
Modified polyolefin tie layers	Excellent compatibility between otherwise incompatible multilayer substrates; high barrier-continuity support	Difficult layer separation; possible recycling constraints in mixed-material structures	Co-extruded multilayer barrier films (EVOH/PA-based structures); flexible food packaging	Excellent	Moderate	Mature	High	Low–moderate	Established; no dedicated positive-list beyond general polyolefin food-contact frameworks
Acrylic and waterborne adhesives	Low VOC emissions; good transparency; suitable for labels, coatings, and paper-based packaging	Lower thermal and moisture resistance than reactive PU systems	Labels, tapes, resealable packaging, coated flexible substrates, paper-based packaging	Good	Moderate–high	Mature	High	Low–moderate	Established; favoured by low-VOC regulatory trends
Conventional hot-melt adhesives	Fast setting; solvent-free processing; high-speed packaging compatibility	Temperature sensitivity; possible migration of tackifiers, waxes, or additives	Carton sealing, case/tray assembly, labeling, high-speed flexible packaging lines	Excellent	Moderate	Mature	High	Low	Established, but subject to growing NIAS/migration scrutiny (e.g., MOAH)
Protein-based adhesives	Renewable origin; good adhesion to polar and cellulosic substrates; useful in labeling and paper packaging	Moisture sensitivity; limited long-term durability; biological variability	Labeling (reusable glass/PET), corrugated board, paper laminates	Moderate	High	Emerging–intermediate	Moderate (feedstock/batch variability)	Moderate	Generally favourable for food contact; no dedicated approval pathway
Polysaccharide-based adhesives	Low cost; renewable and biodegradable; good compatibility with paper and board	Poor adhesion to hydrophobic films; low moisture and thermal resistance	Corrugated board, paper and fibre-based packaging	Moderate	High	Emerging	Moderate–high	Low	Favourable; compostability certification (EN 13432 [9]/ASTM D6400 [10]) required at package level
Modified/reinforced bio-based systems	Improved water resistance, cohesive strength, and multifunctionality compared with native biopolymers	Higher formulation complexity; variable scalability and storage stability	Paper-based and biodegradable film packaging requiring improved water resistance/multifunctionality	Moderate–good	High, formulation-dependent	Emerging	Limited–moderate (variable scalability, storage stability)	Moderate–high	Case-by-case; nanofillers/crosslinkers require dedicated migration assessment
Compostable hot-melt adhesives	Good processability; moderate–high adhesion; compatibility with compostable substrates	Narrower thermal window; formulation-dependent durability and food-contact compliance	Carton sealing, biodegradable laminates, compostable/mono-material flexible packaging	Good	High	Intermediate	Moderate	Moderate	Requires whole-package alignment with EN 13432 [9]/ASTM D6400 [10]
Bio-based PU/NIPU systems	Improved renewable content; potential reduction in hazardous components; good adhesion potential	NIPU systems still limited by slow curing and performance gaps; bio-PU may still rely on isocyanates	Flexible multilayer packaging requiring reduced isocyanate content or renewable polyol content	Good	Moderate–high	Emerging–intermediate	Limited (NIPU: slow curing kinetics)	High	NIPU favourable (avoids isocyanate handling rules) but not yet industrially validated at scale
Functional and hybrid adhesives	Multifunctionality; improved interface control; potential for smart packaging, labeling, and circularity-oriented design	Higher formulation complexity; regulatory, migration, and recycling constraints	Smart/electronic packaging, active labeling, recyclable multilayer tie-layer applications	Good	Moderate–high	Transitional	Low (niche/high-value sectors only)	High	Case-by-case; conductive/reactive additives require dedicated migration evaluation

*Note: The comparison is qualitative and refers to typical behaviour reported for representative adhesive families in packaging applications. Ratings should not be interpreted as absolute rankings, since performance depends strongly on formulation, substrate type, layer architecture, processing conditions, and test method. “Circularity profile” considers recyclability, compostability, removability, and compatibility with end-of-life routes rather than renewable content alone.*

**Table 2:** Biodegradable and bio-based sealants. Overview of the main sealant families investigated for sustainable packaging, summarising their advantages, limitations, and trade-offs in sealability, processability, barrier performance, durability, moisture sensitivity, and compostability.

Category	Main Examples	Main Advantages	Key Limitations
Aliphatic polyester-based sealants	PLA, PBS, PBAT and blends	Renewable origin Industrial compostability	Narrower processing window Sensitive to moisture
Polysaccharide-based sealants	Starch, cellulose, chitosan and derivatives	Abundant and low cost Good biodegradability and compostability	High sensitivity to moisture Limited mechanical strength
Protein-based sealants	Gelatin, casein, whey, soy proteins and blends	Edible and safe for food contact Good barrier performance	Very sensitive to moisture Limited mechanical durability
Advanced and multifunctional bio-sealants	Nanocomposites, coatings, self-healing and active systems	Enhanced mechanical and barrier performance Improved processability and seal strength	Higher formulation complexity and cost Compatibility issues in multilayer structures
Emerging hybrid and coating systems	Bio-based hybrids, sol–gel coatings, hybrids with conventional polymers	Balanced properties and processability Versatile design options	Complex formulation and optimization Compostability depends on total system design

**Table 3:** General characteristics of major aliphatic polyester-based sealants used in biodegradable packaging systems. Comparative overview of PLA, PBS, PBAT, and related blends, summarising their advantages, limitations, and functional roles in biodegradable sealing structures.

Material/System	Main Advantages	Main Limitations	Typical Packaging Role
PLA	Industrial compostability, transparency, stiffness, good printability	Brittleness, narrow sealing window, hydrolytic sensitivity, limited hot-tack robustness	Compostable rigid and semi-rigid films, thermoformed trays, multilayer compostable structures
PBS	Flexibility, lower sealing temperature, broader sealing window, improved hot-tack behaviour	Lower stiffness and moderate thermal resistance	Flexible sealant layers, compostable films, blend component for improved sealability
PBAT	High ductility, toughness, good processability, resistance to brittle failure	Lower stiffness, partial fossil-derived content, limited structural rigidity	Soft phase in compostable multilayer films, flexible packaging, sealing blends
PLA/PBS blends	Improved flexibility and sealability compared with neat PLA	Morphological instability and phase compatibility issues	Compostable flexible packaging systems requiring balanced stiffness and flexibility
PLA/PBAT blends	Reduced seal initiation temperature, improved ductility and hot-tack behaviour	Need for compatibilisation and morphology control	Compostable flexible films and multilayer sealant systems
Compatibilised polyester blends	Enhanced interfacial adhesion, improved mechanical stability and processability	Increased formulation complexity and processing sensitivity	Advanced biodegradable sealing systems for industrial packaging applications

**Table 4:** Standardized and industry-referenced test methods for the principal performance and end-of-life metrics discussed in this review. The table summarises representative standards for peel strength, bond strength, seal strength, seal initiation temperature, hot-tack behaviour, leakage/seal integrity, migration, compostability, and recyclability, providing methodological grounding for the quantitative comparisons presented in Section 4 and Section 5.

Performance Metric	Standard Test Method(s)	Brief Description	Typical Applicable Systems
Peel strength/peel resistance	[121,122,123]	Force required to progressively separate a bonded or sealed interface at a defined angle and rate	Laminates, flexible packaging seals, labels
Bond strength (shear/tensile)	[124,125]	Maximum load sustained by a bonded joint before failure under shear or tensile loading	Structural and laminating adhesives
Seal strength	[121,123]	Maximum force sustained by a heat-sealed joint before failure, typically reported per unit width	Thermoplastic and multilayer sealants
Seal initiation temperature (SIT)	[126]	Minimum sealing temperature at which a defined minimum seal strength is achieved	Thermoplastic and biodegradable sealants
Hot-tack strength	[127]	Resistance of a seal to separation immediately after formation, before complete cooling	High-speed form–fill–seal applications
Leakage/seal integrity (hermeticity)	[128,129]	Detection of seal defects or channels compromising barrier continuity	Multilayer and barrier packaging seals
Migration into food simulants	[130,131]	Quantification of substances transferring from packaging materials into food or food simulants under defined conditions	Food-contact adhesives and sealants
Compostability	[9,10]	Certification scheme evaluating disintegration, biodegradation, and ecotoxicity of packaging under industrial composting conditions	Biodegradable/compostable adhesives and sealants
Recyclability	No single harmonised standard; industry protocols (e.g., CEFLEX guidelines, RecyClass methodology)	Assessment of material separability, purity, and compatibility with existing mechanical recycling streams	Multilayer and mono-material packaging structures

*Note: The reported values throughout this review derive from heterogeneous test protocols differing in specimen geometry, conditioning, and loading rate; direct numerical comparison across studies should therefore be interpreted with caution.*

**Table 5:** Principal performance metrics used for packaging adhesives and their relevance in packaging applications. The table summarizes the most important mechanical, interfacial, processing, and environmental parameters used to evaluate adhesive systems for multilayer, flexible, and paper-based packaging structures.

Performance Metric	Description	Main Influencing Factors	Relevance in Packaging
Bond strength	Ability to transfer loads across substrates	Surface chemistry, curing conditions, adhesive thickness, wettability	Structural integrity of multilayer laminates
Peel resistance	Resistance to progressive interfacial delamination	Viscoelasticity, interface quality, strain localization	Flexible packaging durability and opening behaviour
Cohesive durability	Resistance to creep, fatigue, and environmental degradation	Crosslink density, humidity, thermal exposure	Long-term laminate stability
Aging stability	Resistance to environmental and hydrothermal exposure	Oxidation, UV, sterilization, moisture	Retortable and refrigerated packaging
Processing robustness	Stability during converting and application operations	Viscosity, open time, curing kinetics	High-speed industrial processability
Interfacial compatibility	Adhesion between chemically dissimilar substrates	Surface energy, polarity mismatch, wetting	Multilayer packaging integration
Environmental and regulatory compatibility	Compliance with safety and circularity requirements	VOCs, migration, NIAS, recyclability	Food-contact and sustainable packaging

**Table 8:** Performance-oriented comparison of representative adhesive families used in packaging applications. Qualitative comparison of bond strength, peel resistance, cohesive durability, aging stability, processing robustness, interfacial compatibility, and circularity compatibility.

Adhesive Family	Bond Strength	Peel Resistance	Cohesive Durability	Aging Stability	Processing Robustness	Interfacial Compatibility	Circularity Compatibility	Representative References
Polyurethane and reactive laminating systems	High	High	High	High	High	Excellent	Limited	[16,19]
Acrylic, waterborne, and PSA systems	Moderate	Moderate	Moderate	Moderate	High	Moderate	Moderate–good	[18,26]
Hot-melt adhesive systems	Moderate	Good	Moderate	Limited–moderate	Excellent	Moderate	Moderate	[27,28]
Bio-based and compostable adhesive systems	Limited–moderate	Moderate	Limited	Limited	Moderate	Limited–moderate	Excellent	[32,34]

**Table 9:** Principal performance metrics used for packaging sealants and their relevance in packaging applications. The table summarizes the most important thermal, mechanical, rheological, barrier-related, and environmental parameters commonly used to evaluate sealant systems for flexible, multilayer, and food-packaging structures.

Performance Metric	Description	Main Influencing Factors	Relevance in Packaging	Representative References
Seal strength	Mechanical resistance of the sealed interface under loading	Polymer cohesion, sealing temperature, dwell time, pressure	Package integrity during handling and transport	[66,143]
Seal initiation temperature (SIT)	Minimum temperature at which an acceptable seal is formed	Polymer melting behaviour, crystallinity, sealing conditions	Energy efficiency and processability	[7,144]
Hot-tack strength	Resistance of the seal immediately after sealing while still hot	Melt strength, crystallization rate, cooling behaviour	High-speed packaging operations	[143]
Sealing window	Range of processing conditions yielding acceptable seals	Temperature tolerance, dwell time, pressure stability	Industrial robustness and converting flexibility	[66]
Hermeticity/barrier continuity	Ability to maintain continuous barrier properties without leakage	Seal uniformity, defects, channel formation	Food preservation and barrier reliability	[7]
Contamination tolerance	Ability to seal in the presence of contaminants	Sealant rheology, wetting capability, substrate condition	Food-packaging reliability under real conditions	[66]
Hydrothermal resistance	Retention of seal integrity after humidity or thermal exposure	Polymer stability, additive migration, moisture sensitivity	Retortable and refrigerated packaging	[7]
Circularity compatibility	Compatibility with recycling or compostability routes	Mono-material integration, delamination behaviour	Sustainable and recyclable packaging	[4,54]

**Table 10:** Food-contact safety and migration considerations across the main sealant families discussed in this review. The table links each adhesive family to representative potential migrants and NIAS sources, associated regulatory or safety concerns, typical analytical detection approaches, and mitigation strategies discussed in the literature, distinguishing general regulatory issues from chemistry-specific risks.

Sealant Family	Main Potential Migrants/NIAS Sources	Representative Regulatory/Safety Concern	Typical Analytical Detection Approach	Mitigation Strategies	Reference(s)
Polyolefin-based (LDPE/PP/ionomers)	Oligomers, antioxidants, slip/antiblock additives	Generally low-risk, established food-contact history	GC-MS screening of oligomers/additives	Optimised additive package, compliance with polyolefin positive lists	[7,66]
High-barrier multilayer (EVOH/PVDC/tie layers)	PVDC-related chlorinated by-products, tie-layer grafting agents, coating residues	Chlorine-containing structures under increasing end-of-life scrutiny; layer-interaction effects on migration	Simulant migration testing on the full multilayer structure, GC-MS	PVDC phase-out, EVOH/tie-layer optimisation, whole-structure migration testing	[23,76]
Biodegradable/compostable (PLA/PBS/PBAT, starch/protein-based)	Residual monomers/oligomers (e.g., lactide), plasticisers, biomass-derived additives	Bio-based origin does not guarantee low migration; case-by-case assessment needed	Chromatographic/mass-spectrometric screening	Compostability certification at package level, controlled residual monomer content	[6,32,89]

**Table 13:** Technology readiness and system-level maturity of emerging non-thermal, debondable, and smart sealing/adhesive technologies discussed in Section 3.4 and Section 5.2. The table summarises technology readiness, scalability, relative cost, regulatory maturity, and the main barrier currently limiting industrial adoption for each technology.

Technology	Representative Reference(s)	Technology Readiness	Scalability	Relative Cost	Regulatory Maturity	Main Barrier to Industrial Adoption
Conventional ultrasonic sealing	[113]	Pilot-to-commercial	Moderate	Moderate–high (capital equipment)	Established (uses conventional polymers)	Equipment cost, process re-engineering
HIFU sealing	[114]	Laboratory/pilot	Low (~3 m/min throughput)	High	Not yet standardized	Limited sealing speed
Reversible photocurable/CAN adhesives	[116]	Laboratory/proof-of-concept	Low	High	Not yet assessed for food contact	Photoinitiator replenishment; food-contact approval
Thermoreversible Diels–Alder/NIR-triggered systems	[117]	Laboratory/proof-of-concept	Low	High	Not yet assessed	Complex synthesis; nanoparticle migration unknowns
Mechanically triggered (microcapsule) debonding	[146]	Laboratory	Low	High	Not yet assessed	Encapsulation cost; reproducibility at scale
Water-soluble PVOH tie layer	[147]	Pilot-scale	Moderate	Moderate	Compatible with existing recycling infrastructure	Requires shredding/immersion step; not universally applicable
Light-responsive coumarin tie layer	[148]	Laboratory	Low	High	Not yet assessed	UV equipment integration; migration unknowns
Smart/electronic functional adhesives	[57,59]	Niche-commercial to laboratory	Low (specialized sectors only)	High	Case-by-case; largely unaddressed for food contact	Cost; conductive filler migration; narrow scope

## Data Availability

No new data were created or analyzed in this study. Data sharing is not applicable to this article.
